# Feasibility of FreeSurfer Processing for T1-Weighted Brain Images of 5-Year-Olds: Semiautomated Protocol of FinnBrain Neuroimaging Lab

**DOI:** 10.3389/fnins.2022.874062

**Published:** 2022-05-02

**Authors:** Elmo P. Pulli, Eero Silver, Venla Kumpulainen, Anni Copeland, Harri Merisaari, Jani Saunavaara, Riitta Parkkola, Tuire Lähdesmäki, Ekaterina Saukko, Saara Nolvi, Eeva-Leena Kataja, Riikka Korja, Linnea Karlsson, Hasse Karlsson, Jetro J. Tuulari

**Affiliations:** ^1^Turku Brain and Mind Center, Department of Clinical Medicine, University of Turku, Turku, Finland; ^2^Department of Psychiatry, Turku University Hospital, University of Turku, Turku, Finland; ^3^Department of Radiology, University of Turku, Turku, Finland; ^4^Department of Medical Physics, Turku University Hospital, Turku, Finland; ^5^Department of Radiology, Turku University Hospital, Turku, Finland; ^6^Department of Pediatrics and Adolescent Medicine, Turku University Hospital, University of Turku, Turku, Finland; ^7^Turku Institute for Advanced Studies, University of Turku, Turku, Finland; ^8^Department of Psychology, University of Turku, Turku, Finland; ^9^Centre for Population Health Research, Turku University Hospital, University of Turku, Turku, Finland; ^10^Turku Collegium for Science, Medicine and Technology, University of Turku, Turku, Finland; ^11^Department of Psychiatry, University of Oxford, Oxford, United Kingdom

**Keywords:** brain, child, neuroimaging, brain growth and development, magnetic resonance imaging

## Abstract

Pediatric neuroimaging is a quickly developing field that still faces important methodological challenges. Pediatric images usually have more motion artifact than adult images. The artifact can cause visible errors in brain segmentation, and one way to address it is to manually edit the segmented images. Variability in editing and quality control protocols may complicate comparisons between studies. In this article, we describe in detail the semiautomated segmentation and quality control protocol of structural brain images that was used in FinnBrain Birth Cohort Study and relies on the well-established FreeSurfer v6.0 and ENIGMA (Enhancing Neuro Imaging Genetics through Meta Analysis) consortium tools. The participants were typically developing 5-year-olds [*n* = 134, 5.34 (SD 0.06) years, 62 girls]. Following a dichotomous quality rating scale for inclusion and exclusion of images, we explored the quality on a region of interest level to exclude all regions with major segmentation errors. The effects of manual edits on cortical thickness values were relatively minor: less than 2% in all regions. Supplementary Material cover registration and additional edit options in FreeSurfer and comparison to the computational anatomy toolbox (CAT12). Overall, we conclude that despite minor imperfections FreeSurfer can be reliably used to segment cortical metrics from T1-weighted images of 5-year-old children with appropriate quality assessment in place. However, custom templates may be needed to optimize the results for the subcortical areas. Through visual assessment on a level of individual regions of interest, our semiautomated segmentation protocol is hopefully helpful for investigators working with similar data sets, and for ensuring high quality pediatric neuroimaging data.

## Introduction

There are multiple methodological challenges in pediatric neuroimaging studies that may affect quality of data and comparisons between studies. Magnetic resonance imaging (MRI) requires the subject to lie still while awake, which is more of a challenge with children than with adults (Blumenthal et al., 2002; Poldrack et al., 2002; Theys et al., 2014). This can lead to increased motion artifact. One study, Blumenthal et al. (2002) found that mild, moderate, and severe motion artifact were associated with 4, 7, and 27% loss of total gray matter (GM) volume in segmentation, respectively. Furthermore, subtle motion can cause bias even when a visible artifact is absent (Alexander-Bloch et al., 2016). Another core challenge is the variation in preprocessing and segmentation techniques (Phan et al., 2018b), due to a lack of a “gold standard” processing pipeline for pediatric brain images. Therefore, some studies rightfully emphasize the importance of a validated quality control protocol (Schoemaker et al., 2016).

FreeSurfer^1^ is an open source software suite for processing brain MRI images that is commonly used in pediatric neuroimaging (Ghosh et al., 2010; Black et al., 2012; Ranger et al., 2013; Clark et al., 2014; Roos et al., 2014; El Marroun et al., 2016; Lee et al., 2017; Garnett et al., 2018; Nwosu et al., 2018; Phan et al., 2018b; Al Harrach et al., 2019; Barnes-Davis et al., 2020; Boutzoukas et al., 2020; Wedderburn et al., 2020). The automated FreeSurfer segmentation protocol utilizes surface-based parcellation of cortical regions based on cortical folding patterns and *a priori* knowledge of anatomical structures (further technical information in Dale et al., 1999; Fischl et al., 1999a). The FreeSurfer instructions recommend to visually check and, when necessary, manually edit the data. The manual edits can fix errors in the automated segmentation such as skull-stripping, white matter (WM), or pial errors (errors in the outer border of cortical GM). The FreeSurfer instructions suggest that this process takes approximately 30 min. However, in our experience, this timeframe seems far too short for careful quality assessment and editing.

The time requirement is perhaps the most important practical challenge in manual editing of brain images. Another one is the fact that the edits may lead to inter- and intra-rater bias. Nevertheless, effects of motion artifact must be considered in the segmentation process (Blumenthal et al., 2002), as some systematic errors in pial border, subcortical structures, and the cerebellum have been observed in structural brain images of 5-year-olds without manual edits (Phan et al., 2018b). While a visual check for major errors has obvious benefits, the benefits of manual edits are not as clear in children (Beelen et al., 2020), adolescents (Ross et al., 2021), or adults (McCarthy et al., 2015; Guenette et al., 2018; Waters et al., 2019) as errors that can be manually edited are often small and therefore only have minor effects on cortical thickness (CT), surface area (SA), or volume values. Consequently, they do not necessarily affect the significant findings in group comparisons (McCarthy et al., 2015; Ross et al., 2021) or brain–behavior relationships (Waters et al., 2019). However, we argue that systematic manual edits of the segmented images can help with quality control as they simultaneously maximize the chance to find segmentation errors that can be subsequently fixed.

Quality control is often done by applying a dichotomous pass or fail scale: either by simply excluding the cases with excessive motion artifact (Ranger et al., 2013; Yang et al., 2015; Yang et al., 2016; Garnett et al., 2018; Vanderauwera et al., 2018; Boutzoukas et al., 2020), excluding issues related to pathologies (Ranger et al., 2013; Al Harrach et al., 2019), excluding extreme outlier cases (Nwosu et al., 2018), or it is simply noting that all images were considered to be of sufficient quality without a more detailed description of the criteria (Barnes-Davis et al., 2020). Another approach is to rate the image on a Likert scale from excellent or no motion artifact to unusable (Blumenthal et al., 2002; White et al., 2018). One key challenge with this approach is that the exact borders between categories are very difficult to describe accurately in writing, and terms such as “subtle” and “significant” concentric bands or motion artifact are frequently used to draw the borders (Blumenthal et al., 2002; Shaw et al., 2007). Consequently, even if good intra- and inter-rater reliability can be reached within a study (Shaw et al., 2007), there can be large differences in how different studies define the categories. In many cases, the line of exclusion is drawn between moderate and severe (Lyall et al., 2015) or mild and moderate artifact (Shaw et al., 2007), and either way this fundamentally results in two categories: images with acceptable quality and images with unacceptable quality. Instead of a further quality classification via a Likert scale based on the amount of visible artifact, it might be beneficial to quality check all regions of interest (ROI) separately to verify high quality of the data. Especially considering the fact that the developing brain undergoes multiple non-linear growth patterns (Wilke et al., 2003; Phan et al., 2018b), which may cause issues when utilizing an adult template (Muzik et al., 2000; Yoon et al., 2009; Phan et al., 2018a), and local errors related to this challenge may be missed if quality check is based solely on the severity of visible motion artifact.

In this article, we propose a dichotomous rating scale for inclusion and exclusion of the images segmented with FreeSurfer, combined with a post-processing quality control protocol to visually confirm high quality data on a ROI level. For the automated segmentation tool in this protocol, we chose FreeSurfer based on the following practical advantages: (1) FreeSurfer has been validated for use in children between ages 4 and 11 years (Ghosh et al., 2010), and multiple studies have used FreeSurfer to find brain associations between brain structure and risk factors or cognitive differences in children (Black et al., 2012; Clark et al., 2014; Wedderburn et al., 2020); (2) FreeSurfer provides a method to accurately assess image quality and to fix certain types of errors via Freeview; and (3) Rigorous quality control protocols, such as the one provided by the ENIGMA consortium (Enhancing Neuro Imaging Genetics through Meta Analysis^2^), already exist for FreeSurfer to make final quality assessment on such a level that allows the researchers to exclude single ROIs with imperfect segmentation. We decided to use the ENIGMA quality control protocol, as it is widely used and accepted (Thompson et al., 2020), and has been successfully implemented for both adults (Thompson et al., 2020) and children (Boedhoe et al., 2018; Hoogman et al., 2019). The manual edits instructed by FreeSurfer and rigorous ENIGMA quality control protocol were combined to form the semiautomated segmentation protocol used in the FinnBrain Neuroimaging Lab.

In the current study, we used a subsample of circa 5-year-olds that participated in MRI brain scans as part of the FinnBrain Birth Cohort Study. We give a detailed description of our manual editing and quality control protocol for T1-weighted MRI images in the FreeSurfer software suite. We used the ENIGMA quality control protocol and compare the findings to our protocol. This article aims to make our protocol very explicit and provide some guidelines on how one might assess image quality in a systematic manner across the sample (similar to Griffanti et al., 2017). Furthermore, in a complementary analysis, we compared automated segmentation results between FreeSurfer and the statistical parametric mapping (SPM^3^) based computational anatomy toolbox (CAT12^4^) to assess to the level of agreement. Finally, we compared the standard recon-all to other optional flags in FreeSurfer.

## Materials and Methods

This study was conducted in accordance with the Declaration of Helsinki, and it was approved by the Joint Ethics Committee of the University of Turku and the Hospital District of Southwest Finland (07.08.2018) §330, ETMK: 31/180/2011.

### Participants

The participants are part of the FinnBrain Birth Cohort Study^5^ (Karlsson et al., 2018), where 5-year-olds were invited to neuropsychological, logopedic, neuroimaging, and pediatric study visits. For the neuroimaging visit, we primarily recruited participants that had a prior visit to neuropsychological measurements at circa 5 years of age (*n* = 141/146). However, there were a few exceptions: three participants were included without a neuropsychological visit, as they had an exposure to maternal prenatal synthetic glucocorticoid treatment (recruited separately for a nested case–control sub-study). The data additionally includes two participants that were enrolled for pilot scans. We aimed to scan all subjects between the ages 5 years 3 months and 5 years 5 months, and 135/146 (92%) of the participants attended the visit within this timeframe (reasons to scan outside the timeframe include, for example, the family moving the visit to a later date). The exclusion criteria for this study were: (1) born before gestational week 35 (before gestational week 32 for those with exposure to maternal prenatal synthetic glucocorticoid treatment), (2) developmental anomaly or abnormalities in senses or communication (e.g., blindness, deafness, and congenital heart disease), (3) known long-term medical diagnosis (e.g., epilepsy and autism), (4) ongoing medical examinations or clinical follow up in a hospital (meaning there has been a referral from primary care setting to special health care), (5) child use of continuous, daily medication (including per oral medications, topical creams, and inhalants. One exception to this was desmopressin (^®^Minirin) medication, which was allowed), (6) history of head trauma (defined as concussion necessitating clinical follow up in a health care setting or worse), (7) metallic (golden) ear tubes (to assure good-quality scans), and routine MRI contraindications.

**FIGURE 1:**
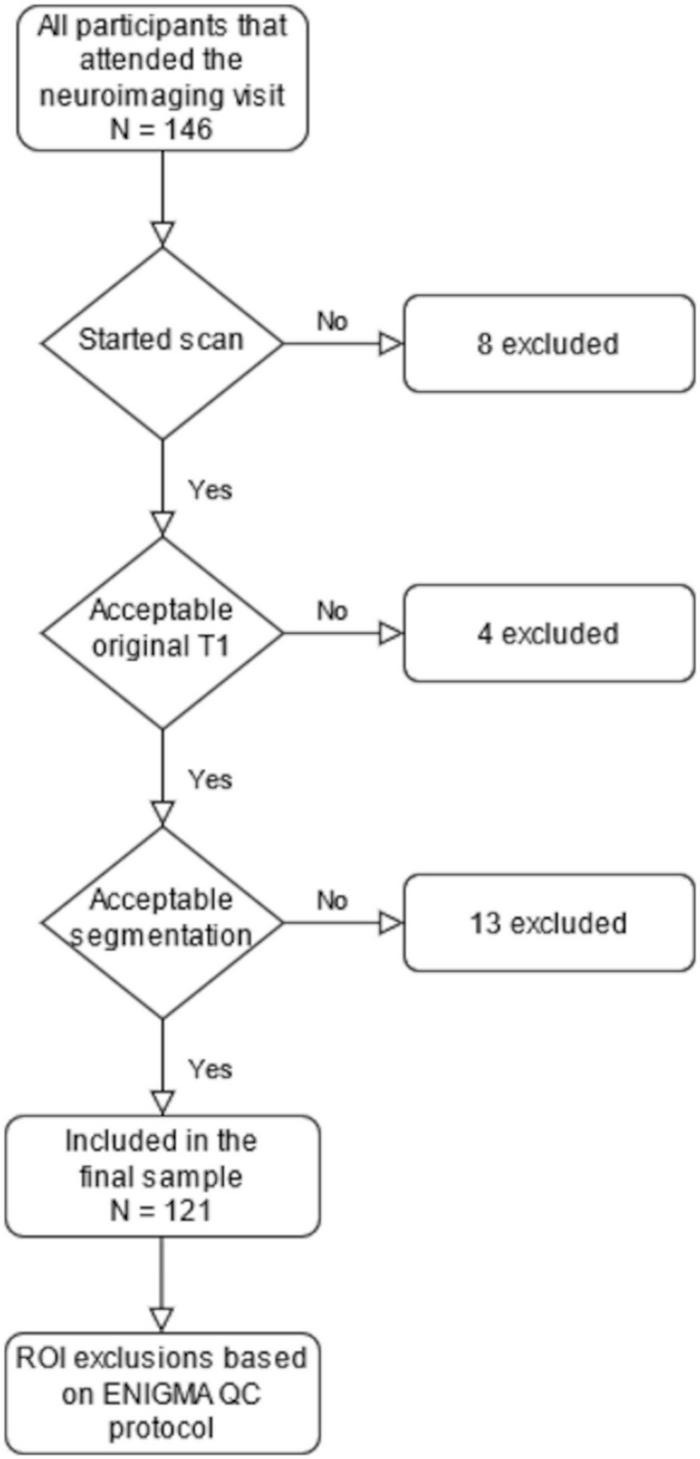
A flowchart depicting the steps leading to our final sample size of 121. The region of interest (ROI) exclusions are presented in Supplementary Table 2.

In the current study, we used a subsample (approximately two thirds of the full sample) that consists of the participants that were scanned before a temporary stop to visits due to the restrictions caused by the coronavirus disease 2019 (COVID-19) pandemic. The scans were performed between 29 October, 2017 and 1 March, 2020. We contacted 415 families and reached 363 (87%) of them. In total, 146 (40% of the reached families) participants attended imaging visits (one pair of twins, one participant attended twice, and only the latter scan was included). Eight of them did not start the scan, and four were excluded due to excess motion artifact in the T1-image. Thereafter, 134 T1 images (mean age 5.34 years, SD 0.06 years, range 5.08–5.22 years, 72 boys, 62 girls) entered the processing pipelines. Supplementary Table 1 presents the demographic data as recommended in our earlier review (Pulli et al., 2019). A flowchart depicting the formation of the final sample through the different exclusion steps is presented in Figure 1.

### The Study Visits

All MRI scans were performed for research purposes by the research staff (one research nurse, four Ph.D. students, and two MR technologists). Before the visit, each family was personally contacted and recruited via telephone calls by a research staff member. The scan preparations started with the recruitment and at home training. We introduced the image acquisition process to the parents and advised them to explain the process to their children and confirm child assent before the follow up phone call that was used to confirm the willingness to participate. Thereafter, we advised the parents to use at home familiarization methods such as showing a video describing the visit, playing audio of scanner sounds, encouraging the child to lie still like a statue (“statue game”), and practicing with a homemade mock scanner, e.g., a cardboard box with a hole to view a movie through. The visit was marketed to the participants as a “space adventure,” which is in principle similar to the previously described “submarine protocol” (Theys et al., 2014) but the child was allowed to come up with other settings as well. A member of the research staff made a home visit before the scan to deliver earplugs and headphones, to give more detailed information about the visit, and to answer any remaining questions. An added benefit of the home visit was the chance to meet the participating child and that way start the familiarization with the research staff.

At the start of the visit, we familiarized the participant with the research team (research nurse and a medically trained Ph.D. student) and acquired written informed consent from both parents. This first portion of the visit included a practice session using a non-commercial mock scanner consisting of a toy tunnel and a homemade wooden head coil. Inexpensive non-commercial mock scanners have been shown to be as effective as commercial ones (Barnea-Goraly et al., 2014). The participants brought at least one of their toys that would undergo a mock scan (e.g., an MRI compatible stuffed animal they could also bring with them into the real scanner). The researcher played scanner sounds on their cell phone during the mock scan and the child could take pictures of the toy lying still and of the toy being moved by the researcher to demonstrate the importance of lying still during the scan. Communication during the scan was practiced. Overall, these preparations at the scan site were highly variable as we did our best to accommodate to befit the child characteristics (e.g., taking into account the physical activity and anxiety) in cooperation with the family. Finally, we served a light meal of the participant’s choice before the scan.

The participants were scanned awake or during natural sleep. One member of the research staff and parent(s) stayed in the scanner room throughout the whole scan. During the scan, participants wore earplugs and headphones. Through the headphones, they were able to listen to the movie or TV show of their choice while watching it with the help of mirrors fitted into the head coil (the TV was located at the foot of the bed of the scanner). Some foam padding was applied to help the head stay still and assure comfortable position. Participants were given a “signal ball” to throw in case they needed or wanted to stop or pause the scan (e.g., to visit the toilet). If the research staff member noticed movement, they gently reminded the participant to stay still by lightly touching their foot. This method of communication was agreed on earlier in the visit and was planned to convey a clear signal of presence while minimizing the tactile stimulation. Many of the methods used to reduce anxiety and motion during the scan have been described in earlier studies (Epstein et al., 2007; Greene et al., 2016).

All images were viewed by one neuroradiologist (RP) who then consulted a pediatric neurologist (TL) when necessary. There were four (out of 146, 2.7%) cases with an incidental finding that required consultation. All four cases initially entered the FreeSurfer processing pipeline and three were included in the final ROI based analyses. The protocol with incidental findings has been described in our earlier work (Kumpulainen et al., 2020), and a separate report of their incidence is in preparation for the eventual full data set.

### Magnetic Resonance Imaging Data Acquisition

Participants were scanned using a Siemens Magnetom Skyra fit 3T with a 20-element head/neck matrix coil. We used Generalized Autocalibrating Partially Parallel Acquisition (GRAPPA) technique to accelerate image acquisition [parallel acquisition technique (PAT) factor of 2 was used]. The MRI data was acquired as a part of max. 60-min scan protocol. The scans included a high resolution T1 magnetization prepared rapid gradient echo (MPRAGE), a T2 turbo spin echo (TSE), a 7-min resting state functional MRI, and a 96-direction single shell (b = 1,000 s/mm^2^) diffusion tensor imaging (DTI) sequence (Merisaari et al., 2019) as well as a 31-direction with b = 650 s/mm^2^ and a 80-direction with b = 2,000 s/mm^2^. For the purposes of the current study, we acquired high resolution T1-weighted images with the following sequence parameters: repetition time (TR) = 1,900 ms, echo time (TE) = 3.26 ms, inversion time (TI) = 900 ms, flip angle = 9 degrees, voxel size = 1.0 × 1.0 × 1.0 mm^3^, and field of view (FOV) 256 × 256 mm^2^. The scans were planned as per recommendations of the FreeSurfer developers.^6^

### Data Processing

#### FreeSurfer

Cortical reconstruction and volumetric segmentation for all 134 images were performed with the FreeSurfer software suite, version 6.0.0.^7^ We selected the T1 image with the least motion artifact (in case there were several attempts due to visible motion during scan) and then applied the “recon-all” processing stream with default parameters. It begins with transformation to Talaraich space, intensity inhomogeneity correction, bias field correction (Sled et al., 1998), and skull-stripping (Ségonne et al., 2004). Thereafter, WM is separated from GM and other tissues and the volume within the created WM–GM boundary is filled. After this, the surface is tessellated and smoothed. After these preprocessing steps are completed, the surface is inflated (Fischl et al., 1999a) and registered to a spherical atlas. This method adapts to the folding pattern of each individual brain, utilizing consistent folding patterns such as the central sulcus and the sylvian fissure as landmarks, allowing for high localization accuracy (Fischl et al., 1999b). FreeSurfer uses probabilistic approach based on Markov random fields for automated labeling of brain regions. Cortical thickness is calculated as the average distance between the WM–GM boundary and the pial surface on the tessellated surface (Fischl and Dale, 2000). The cortical thickness measurement technique has been validated against post-mortem histological (Rosas et al., 2002) and manual measurements (Kuperberg et al., 2003; Salat, 2004).

#### FreeSurfer Manual Edits and the Freeview Quality Control Protocol

We used Freeview to view and edit the images using the standard command recommended by the FreeSurfer instructions with the addition of the Desikan–Killiany atlas that allowed us to correctly identify the ROIs where errors were found. Images with excess motion artifact or large unsegmented regions (extending over multiple gyri, examples provided in Supplementary Figure 1) were excluded. There were 13 participants that were excluded due to erroneous segmentation. The images that passed the initial quality check were then manually edited (the time required for manual editing ranged from 45 min in high quality images to over 3 h in images with a lot of artifact, taking approximately 2 h on average). All images were examined in all three directions one hemisphere at a time and the edits were made for every slice regardless of the ROI in question. Subsequently, we ran the automated segmentation process again as suggested by FreeSurfer instructions. The images were then inspected again for errors, and the ROIs with errors that affect WM–GM or pial borders were excluded in the Freeview quality control protocol. The Freeview protocol presented in this study was adapted locally for the FinnBrain Neuroimaging Lab as a method to assess errors in a slice-by-slice view from the official quality control procedure provided in the FreeSurfer instructions.^8^ We also provide a practical application manual in Supplementary Material (Data Sheet 2, pages 3–9, FreeSurfer editing) that we give to new researchers when they start practicing the FinnBrain manual editing and quality control protocol.

##### Errors in Borders

**FIGURE 2:**
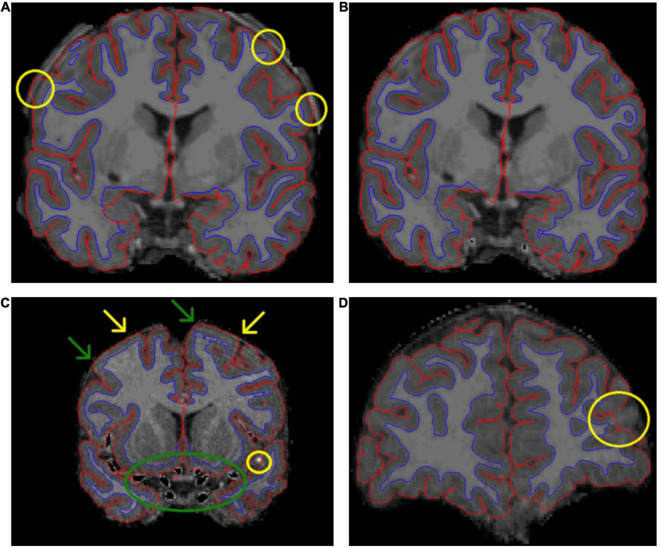
A presentation of some common errors and fixes related to the pial border and non-brain tissues. **(A)** Demonstrates how skull fragments can cause errors in pial border (yellow circles). **(B)** Presents the same subject with skull fragments removed. In panel **(C)**, arteries were removed (green circle). We removed voxels with an intensity between 130 and 190, and therefore some parts of arteries were not removed (yellow circle). **(C)** Also demonstrates the challenges with artifact, meninges, and the pial border. In some areas, the pial border may extend into the meninges (yellow arrows). Meanwhile, at the other end of the same gyrus, the border may seem correct (green arrows). It is difficult to fix these errors manually. Additionally, the visible motion artifact adds further challenges to manual edits of the pial border. In panel **(D)**, the pial border cuts through a gyrus.

The automatically segmented images generated by FreeSurfer software suite were visually inspected and the found errors were either manually corrected or the ROI with the error was simply excluded depending on the type of error. Excess parts of the skull were removed where the pial border was affected by them (Figures 2A,B). Arteries were removed to avoid segmentation errors between arteries and WM (especially relevant for anterior temporal areas and the insulae). This was done by setting the eraser to only delete voxels with intensity between 130 and 190 in the brainmask volume. The arteries were removed throughout the image with no regard to whether they caused issues in the segmentation on that specific slice. An example can be seen in Figure 2C. In cases where an error appeared in a junction between ROIs, all adjoining ROIs were excluded.

One typical error was that parts of the superior sagittal sinus (SSS) were included within the pial border. We stopped editing the SSS after an interim assessment as it was an arduous task with little effect on final results. All information regarding SSS edits is presented in Supplementary Material (Data Sheet 2, pages 10–14, Superior sagittal sinus).

In addition, there were errors that could not be fixed easily. In some cases, the pial border may cut through the cortex (Figure 2D shows an error in the left rostral middle frontal region). In these cases, the remaining GM mask is too small, and this error cannot be easily fixed in Freeview. Manual segmentation of a T1 image is labor intensive and hard to conduct reliably with 1 mm^3^ resolution even when the edits would cover small areas. Moreover, the FreeSurfer instructions do not recommend this approach. Additionally, the WM mask edits recommended in FreeSurfer instructions would not fix all cases where the cortical segmentation is too thin, as the WM mask often seemed adequate in these areas (an example presented in Supplementary Figure 2). Therefore, we simply had to exclude the ROI(s) in question.

**FIGURE 3:**
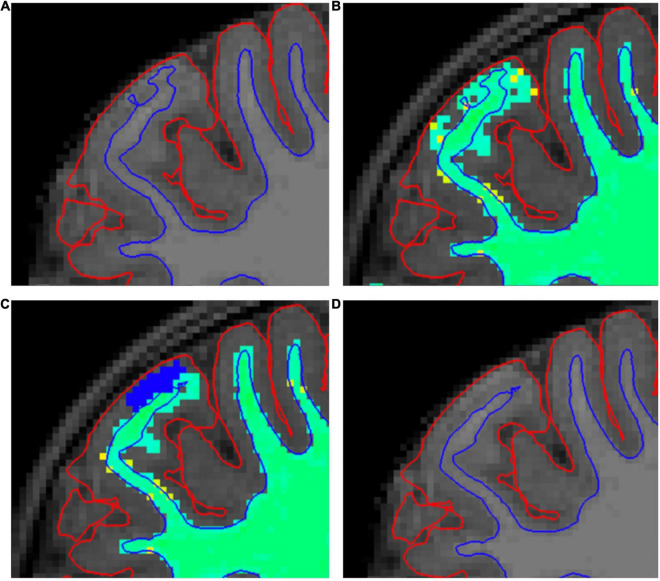
A demonstration of our white matter (WM) mask editing protocol. **(A)** Shows a typical error in the border between white and gray matter (WM–GM border), where it extends too close to the pial border. Errors such as this are searched for in the “brainmask” volume **(A,D)**. **(B)** Shows the same error in “wm” volume with “Jet” colormap **(B,C)**. **(C)** Shows how we fixed these errors by erasing the erroneous WM mask (blue voxels). **(D)** Shows the final result after the second recon-all.

**FIGURE 4:**
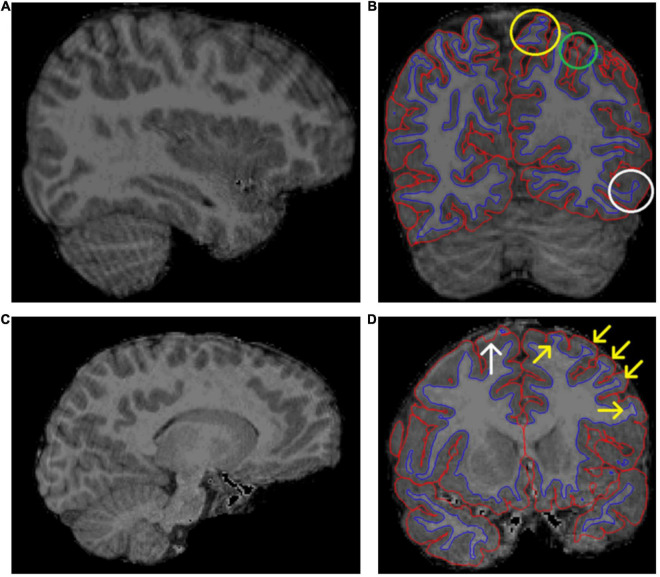
Two examples of excluded brain images. **(A)** Shows “waves” throughout the image, marking motion artifact. **(B)** Shows the same subject as in panel **(A)** in a coronal view and borders visible. This image shows motion artifact related errors in the border between white and gray matter (WM–GM border), denoted by the yellow circle. Additionally, there is potential unsegmented area due to motion artifact (green circle) and poor contrast between WM and GM (white circle). **(C,D)** Show another excluded subject. The motion artifact in panel **(C)** is not as pronounced as in panel **(A)**. However, **(D)** still shows some typical errors for images with much artifact. There is a clear pial error (white arrow). Additionally, the yellow arrows show typical cases, where the “ringing” causes the WM mask to “widen” where the actual WM meets the ringing motion artifact.

Small errors of the WM–GM border were prevalent throughout the brain. The corrections were made by erasing excess WM mask. This process is demonstrated in Figure 3. WM–GM border was inspected after the manual edits. A continuous error of at least ten slices in the coronal view led to exclusion of all the ROIs directly impacted by the error. Furthermore, ubiquitous errors in the WM–GM border, as markers of motion artifact, led to exclusion of the whole brain (as in Figure 4).

Furthermore, there are some error types that cannot be easily fixed but also do not warrant exclusion. One such problem is that the pial border often extends into the cerebrospinal fluid or meninges around the brain (Supplementary Figure 3). The issue with this type of error is that sometimes the real border between GM and the surrounding meninges cannot be denoted visually and therefore the error cannot be reliably fixed. This problem is further complicated by the fact that motion artifact may mimic the border between GM and meninges making the visual quality control challenging (Figure 2C and Supplementary Figure 4). In addition to motion, fat shift can also cause this type of artifact. The amount of fat shift in images is dependent on the imaging protocol, more specifically the bandwidth of the acquisition.

There were some minor incongruities in multiple images. A common example can be seen in Supplementary Figure 5, where there seems to be a potential error in the pial border. Areas like this look normal in other planes. A less common example is shown in Supplementary Figure 6, where there is an apparent discontinuation in the WM–GM border. Similarly, there was no discontinuation in other planes. Both these minor incongruities were considered partial volume effects related to the presentation of a 3D surface in 2D slices. Therefore, both cases were included.

##### Errors in Cortical Labeling

**FIGURE 5:**
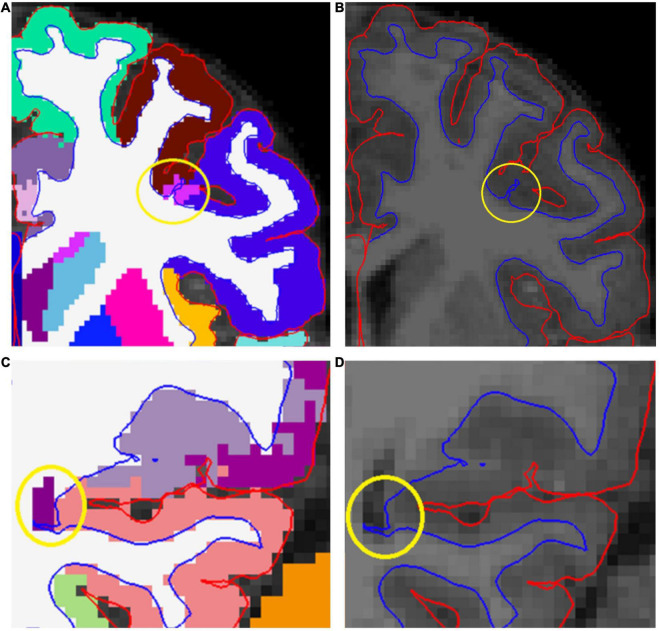
**(A,B)** Show a white matter (WM) hypointensity that affects the border between white and gray matter (WM–GM border), denoted by a yellow circle. **(C,D)** Show how the posterior part of the lateral ventricle causes distortion to the WM–GM border (yellow circle). If the error was not successfully fixed, all regions adjoining the error were excluded.

A common issue was the presence of WM hypointensities in the segmented images. They sometimes erroneously appeared in the cortex. These errors were typically small and did not cause errors in pial or WM–GM borders (Supplementary Figure 7), and in those cases did not require exclusion. The hypointensities themselves were rarely successfully fixed by editing the WM mask and therefore were left unedited unless they caused errors in the GM–WM border. In those cases, removing the WM mask fairly often fixed the error in the border, although frequently the incorrect hypointensity label still remained in the WM segmentation. We tried to fix the errors in the WM–GM border and when unsuccessful, we simply excluded the ROI in question from analyses (Figures 5A,B). Of note, these errors can only be seen with the anatomical labels as overlays, unless they affect the WM–GM border.

One typical error occurred at the posterior end of the lateral ventricles, where it may cause segmentation errors in the adjacent cortical regions, typically the precuneus and the lingual gyrus. These regions were excluded from analyses when there was a distortion in the GM–WM border (Figures 5C,D), and included when there was no distortion in the border (Supplementary Figure 8). Unfortunately, hypointensities often appeared in ROI junctions, leading to exclusion of multiple regions due to one error (Supplementary Figure 9). Similar errors were seen in the ENIGMA protocol as well (Supplementary Figure 10).

##### Errors in Subcortical Labeling

Putamen was often mislabeled by FreeSurfer in our sample. Errors were addressed by adding control points, but the edits were largely unsuccessful. Consequently, we are currently working on separately validating subcortical segmentation procedures for our data (Lidauer et al., 2021). All information regarding the subcortical labeling is presented in Supplementary Material (Data Sheet 2, pages 15–16, Subcortex).

#### ENIGMA Quality Control Protocol

After the quality control that entailed manual edits, we conducted a quality check with the ENIGMA Cortical Quality Control Protocol 2.0 (April 2017).^9^ Therein, the FreeSurfer cortical surface measures were extracted and screened for statistical outliers using R^10^ and visualized via Matlab (Mathworks) and bash scripts. Visual representations of the external 3D surface and internal 2D slices were generated and visually inspected according to the instructions provided by ENIGMA in https://drive.google.com/file/d/0Bw8Acd03pdRSU1pNR05kdEVWeXM/view (at the time of writing). The ENIGMA Cortical quality check instructions remark how certain areas have a lot of anatomical variation and therefore they note the possibility to be more or less stringent in their quality control. Considering this and the fact that the example images provided in the ENIGMA instructions are limited in number and as such cannot show every variation, we deemed necessary to describe how we implemented these instructions in our sample.

##### The External View

We started by viewing the external image. The pre- and postcentral gyri were assessed for meninge overestimations, which can manifest as “spikes” (Supplementary Figure 11A) or flat areas (Supplementary Figure 11B). These error types were rare in our sample. These cases were excluded as instructed.

**FIGURE 6:**
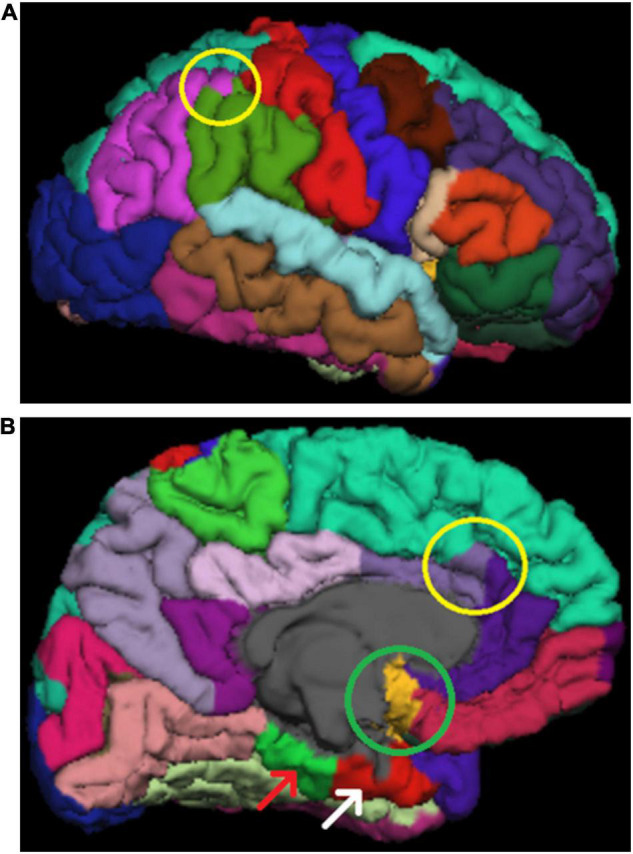
**(A)** Shows an error (yellow circle) where the inferior parietal area (purple) cuts through a whole gyrus in the supramarginal region (green). This area has a lot of variation and only clear errors led to exclusion in our ENIGMA quality control protocol. **(B)** Shows insula overestimation in the midline (green circle). Furthermore, the poor image quality can be seen the areas adjacent to the base of the skull, such as parahippocampal (green area denoted by a red arrow) and entorhinal (red area denoted be a white arrow). Additionally, there is an error in the border between superior frontal and caudal anterior cingulate. This border should follow the sulcal line. The rostral anterior cingulate was not considered erroneous in these cases.

The supramarginal gyrus has a lot of anatomical variability and when quality checking it, we decided to be lenient as suggested by the ENIGMA instructions. We only excluded cases where the border between supramarginal and inferior parietal regions cuts through a gyrus, leading to discontinuous segments in one of the regions (Figure 6A). In some rare cases, this type of error also happened with the postcentral gyrus (Supplementary Figure 12), and these cases were also excluded. Similarly, in cases with supramarginal gyrus overestimation into the superior temporal gyrus, we only excluded clear errors (examples presented in Supplementary Figure 13).

One commonly seen error is insula overestimation into the midline (Figure 6B). In these cases, we exclude insula and the region(s) adjacent to it in the midline (e.g., the medial orbitofrontal region in the case of Figure 6B).

The border between the superior frontal region and the cingulate cortex (Figure 6B and Supplementary Figure 14) is one typical place for errors. A prominent paracingulate sulcus, that is more common on the left than on the right hemisphere, may cause underestimation of the cingulate cortex and consequently overestimation of the superior frontal region. This was typically seen on the left caudal anterior cingulate (Figure 6B), where we excluded the cases where the border did not follow sulcal lines anteriorly (as was demonstrated in the image examples in the instructions). In rare cases the border between posterior cingulate and superior frontal region was affected (Supplementary Figure 14), and these were also excluded.

The pericalcarine region was overestimated in some cases. According to the instructions cases where the segmentation is confined to the calcarine sulcus should be accepted. Therefore, we excluded cases where the pericalcarine region extended over a whole gyrus into the lingual gyrus or the cuneus. An example can be seen in Supplementary Figure 15.

Cases of superior parietal overestimation were excluded as instructed. These errors were rare in our sample. Similarly, errors in the banks of the superior temporal sulcus were excluded as instructed.

The border between the middle and inferior temporal gyrus was not assessed, as the instructions suggested that most irregularities seen there are normal variants or relate to the viewing angle.

Similarly, we did not quality check the entorhinal/parahippocampal regions in the external view, as there is a lot of variation in the area. The ENIGMA instructions describe underestimations in 70–80% of cases. Furthermore, this region looks poor in practically all images (e.g., in Figure 6B) as do all the regions adjacent to the base of the skull and therefore, in our opinion, the quality assessment in those regions requires additional procedures, that are beyond the scope of the current study, to confirm their usability in statistical analyses.

##### The Internal View

In the internal view, regions with unsegmented GM were excluded. These errors often reflect WM hypointensities seen in Freeview (Supplementary Figure 10). Interestingly, even quite large hypointensities do not necessarily equate to errors in the borders set by FreeSurfer and therefore do not always have an adverse effect on CT calculations.

Temporal pole underestimations were sometimes seen. However, the cases were rarely as clear as presented in the instructions. Therefore, we had to use both coronal and axial views to assess the situation and make exclusions when both views supported an error in segmentation.

**FIGURE 7:**
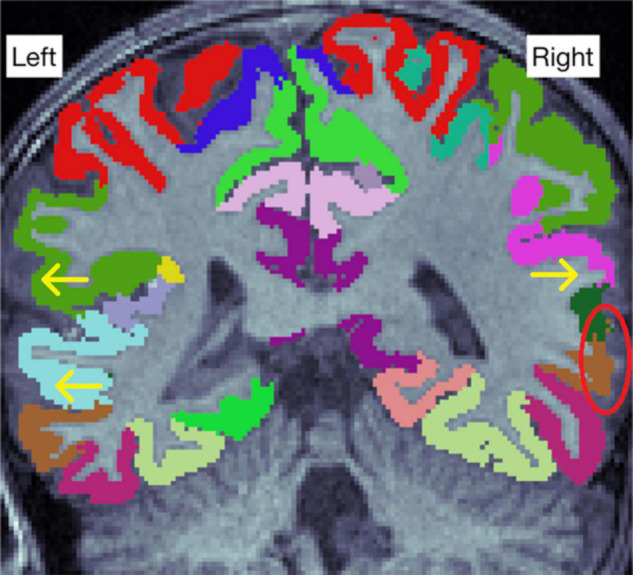
There are some visible errors in the lateral parts of the image (arrows). An especially clear error is denoted by the red circle, where some white matter is seen outside the cortical segmentation.

One of the errors commonly seen in our sample was the erroneous pial surface delineation in the lateral parts of the brain. This was particularly prevalent in the middle temporal gyri (Supplementary Table 2). Notably, it is possible to attempt fixing these types of topological errors, e.g., by using control points or brainmask edits. Some previous studies (e.g., Ross et al., 2021) have done this. They reported average editing time of 9, 5 h, approximately quadruple our editing time, and concluded that the edits did not affect conclusions. Therefore, this type of edit was omitted as too time-consuming and challenging compared to the expected effect on results. The ROIs affected by these errors were excluded from analyses. This error was assessed from 2D slices, wherein what seems to be an error may be caused by partial volume effects. For example, in Supplementary Figure 16A, there seems to be a possible error on the right middle temporal region. If we look at the same image in Freeview, the same position seems to be segmented normally, especially when confirmed in the axial view (Supplementary Figures 16B,C). Consequently, we only made exclusion when clear errors were seen in two adjacent slices. Particularly clear example of this can be seen in Figure 7, where the WM extends outside the segmentation. The error is also visible in the external view, where these regions do not appear as smooth as normally (Supplementary Figure 17), however the decisions to exclude a ROI were always made based on the internal view. This kind of error was significantly harder to recognize in Freeview and represents the most striking difference in results between the ENIGMA and Freeview quality control protocols.

##### Statistical Outliers

After the systematic viewing of all the problem regions, we inspected the statistical outliers. This rarely led to new exclusions, as many of the statistical outliers were among the excluded subjects or the outliers were ROIs where the instructions did not give any tools to assess whether they were correct. Therefore, we had to simply double check the internal view to rule out segmentation errors.

##### Enhancing Neuro Imaging Genetics Through Meta Analysis Exclusion Differences Between Edited and Unedited Images

We performed the full ENIGMA quality control protocol for all edited images that were included in the ROI based analyses (*n* = 121). To assess how manual edits affect the number of excluded regions, we also performed the ENIGMA quality control protocol on a half sample (*n* = 61) of unedited images. In borderline cases (mostly regarding the borders between the supramarginal and superior temporal gyri as well as between the caudal anterior cingulate and superior frontal gyri) we consulted the ENIGMA quality control protocol of the edited images, to make the same ruling if the error was similar. Likewise, in the cases where the edited image passed the internal or external view without any ROI exclusions, but did not pass in the unedited version, the images were directly compared to each other to ensure the reason for not passing is an objective difference, as opposed to a human error or a different ruling in a borderline case.

#### Exclusions

We decided to use a dichotomous rating scale: pass or fail. The amount of motion artifact (marked by “concentric rings” or “waves”) and the clarity of the WM–GM border were assessed from the original T1 image. In borderline cases, we ran the standard recon-all and made new assessment based on the segmented image. Massive segmentation errors such as large missing areas or ubiquitous errors in WM–GM border were reasons for exclusion. Additionally, ENIGMA exclusion criteria were implemented as instructed. In some borderline cases, another expert rater assessed the image quality and agreement was reached to either include or exclude the image. Some images that were considered for inclusion but excluded after the first recon-all can be seen in Figure 4. These images had significantly more artifact than other images in our sample, although arguably they could have been included since the amount of artifact could be described as “moderate.” However, we decided to implement strict exclusion criteria to ensure high quality of data.

#### Alternate Processing: Optional Registration Flags in FreeSurfer

We compared the FreeSurfer default recon all to recon-all with the “-mprage” and “-schwartzya3t-atlas” optional flags. All information regarding optional flags analyses is presented in Supplementary Material (Data Sheet 2, pages 17–18, Optional flags).

#### Alternate Processing: CAT12

A previous study conducted in the elderly demonstrated good agreement between FreeSurfer and CAT12 estimates of CT (*R*^2^ = 0.83), although CAT12 produced systematically higher values than FreeSurfer (Seiger et al., 2018). Therefore, we decided to explore the agreement between the two software in a pediatric population. All information regarding CAT12 analyses is presented in Supplementary Material (Data Sheet 2, pages 19–25, CAT12).

### Alternate Quality Control: Qoala-T

Qoala-T is a supervised learning tool for quality control of automated labeling processed in FreeSurfer, and it is particularly intended for use in analysis of pediatric datasets (Klapwijk et al., 2019). We compared Qoala-T scores from all 134 participants that entered the FreeSurfer segmentation protocol, and the results are reported in Supplementary Material (Data Sheet 2, pages 26–29, Qoala-T).

### Statistics

Statistical analyses were conducted using the IBM SPSS Statistics for Windows, version 25.0 (IBM Corp., Armonk, NY, United States). The ROI data was confirmed to be normally distributed using JMP Pro 15 (SAS Institute Inc., Cary, NC, United States) based on visual assessment and the similarity of mean and median values.

To compare the differences between the included (the participants that were included in ROI based analyses, *n* = 121) and excluded (all participants that lacked usable T1 data, *n* = 25) groups, we performed independent samples *t*-tests for age from birth at scan, gestational age at scan, gestational age at birth, birthweight, maternal age at term, and maternal body mass index (BMI) before pregnancy. In addition, we conducted Chi-Square tests for child gender, maternal education level (three classes: 1 = Upper secondary school or vocational school or lower, 2 = University of applied sciences, and 3 = University), maternal monthly income estimate after taxes (in euros, four classes: 1 = 1,500 or less, 2 = 1,501–2,500, 3 = 2,501–3,500, and 4 = 3,501 or more), maternal alcohol use during pregnancy (1 = yes, continued to some degree after learning about the pregnancy, 2 = yes, stopped after learning about the pregnancy, and 3 = no), maternal tobacco smoking during pregnancy (1 = yes, continued to some degree after learning about the pregnancy, 2 = yes, stopped after learning about the pregnancy, and 3 = no), maternal history of disease (allergies, depression, asthma, anxiety disorder, eating disorder, chronic urinary tract infection, autoimmune disorder, hypercholesterolemia, and hypertension), and maternal medication use at gestational week 14 (non-steroidal anti-inflammatory drugs, thyroxin, selective serotonin reuptake inhibitor [SSRI] or serotonin–norepinephrine reuptake inhibitor [SNRI], and corticosteroids), or at gestational week 34 (thyroxin, SSRI or SNRI, corticosteroids, and blood pressure medications). The categories in history of disease and medication during pregnancy were only included in statistical analyses, when there were at least four participants that had history of the disease or used the medication (to limit the chance of false positives).

To compare the exclusion rates between Freeview and ENIGMA quality control protocols, as well as between ENIGMA quality control protocols of edited and unedited images, we conducted Chi-Square tests (among all datapoints, single ROIs, and internal/external view passes in ENIGMA).

The inclusion criterion for the ROI based comparisons was passing the ENIGMA quality control protocol. To compare edited FreeSurfer to unedited FreeSurfer, we conducted a paired samples *t*-test. We calculated the absolute values of the change in CT between unedited and edited images for each ROI separately using the following formula: (C_D_/C_U_) * 100%, where C_D_ is the absolute value of the difference in mean CT between edited and unedited images and C_U_ is the mean CT in the unedited images. Furthermore, we conducted a paired samples *t*-test with the mean CT values from all ROIs to measure the change between edited and unedited images. The same analyses were performed for WM SA and GM volume.

To assess the effects of manual editing and quality control on group comparison and brain structural asymmetry results, we conducted independent samples *t*-tests for sex differences in CT, SA, and volume measurements between a sample without quality control (*n* = 121 for every ROI) and the quality-controlled sample (maximum *n* = 121, where number of included ROIs varies). Using these same samples, we also conducted paired samples *t*-tests for the 34 ROIs in both hemispheres to examine structural asymmetry. Supplementary Material, Data Sheet 3 output was created using JASP 0.16.1 (JASP Team, 2022).^11^

All significances were calculated 2-tailed (α = 0.05). To adjust for multiple comparisons in ROI-based analyses, we conducted the Bonferroni correction by setting the *p* value to 0.05 divided by the number of comparisons (=the number of ROIs = 68), resulting in *p* = 0.000735. We notify that the *p* value cut off for the current study is somewhat arbitrary and thus we also report the raw *p* values in the tables.

## Results

### Demographics

There were no significant differences between the included and excluded subjects’ age from birth at scan, gestational age at scan, gestational age at birth, birth weight, maternal age at term, maternal education level, maternal monthly income, maternal history of disease, maternal alcohol use during pregnancy, or maternal tobacco smoking during pregnancy. There was a significant difference in maternal BMI before pregnancy (*p* = 0.03). In the included group, mean maternal BMI was 23.9 (*n* = 121) vs. 26.0 in the excluded group (*n* = 24, information from one participant missing). Two types of medication were more common in the excluded group: SSRI or SNRI medication at 14 gestational weeks (*p* = 0.03; included group 109 no, 3 yes; excluded group 20 no, 3 yes) and blood pressure medication at 34 gestational weeks (*p* = 0.03; included group 113 no, 3 yes; excluded group 21 no, 3 yes). In addition, there was a marginally significant difference in SSRI/SNRI use at 34 gestational weeks (*p* = 0.06; included group 112 no, 4 yes; excluded group 21 no, 3 yes). Of note, these results are not optimal to determine whether the listed early exposures are associated with poorer image quality as such but such comparisons may be useful to conduct before final analyses in any data set (and are also included for descriptive purposes) (please see related articles: A. Rodriguez et al., 2008; Alina Rodriguez, 2010; Buss et al., 2012; Chen et al., 2014; Tanda and Salsberry, 2014; Edlow, 2017; Morales et al., 2018).

### Comparison Between Unedited and Manually Edited FreeSurfer Segmentations

#### Cortical Thickness

The difference in CT was not significant after Bonferroni correction in 57/68 (83.8%) regions. Unedited images had significantly larger CT values in 2/68 (2.9%) regions: the right rostral anterior cingulate and right superior temporal regions. Edited images had significantly larger CT values in 9/68 (13.2%) regions: the left and right caudal middle frontal, left and right inferior temporal, left and right superior parietal, right precentral, right superior frontal, and right supramarginal regions. The smallest (both absolute and relative) change was observed in the left rostral middle frontal (0.0003 mm, 0.011%) and the largest (both absolute and relative) in the right caudal middle frontal (0.0526 mm, 1.857%) region. The CT changes and raw *p*-values for all ROIs are presented in Supplementary Table 3.

The mean change in absolute CT values between the unedited and edited images was 0.0129 mm (0.441%). When we include the direction of the change in the analysis, edited images had higher CT values (mean 0.00264 mm, 0.0901%), although the difference was not statistically significant (*p* = 0.217).

Pearson correlations between edited and unedited images were calculated by ROI, they all were positive and ranged from 0.725 in the left insula to 0.984 in the left banks of the superior temporal sulcus region. All remained statistically significant after Bonferroni correction. The correlations are displayed in Supplementary Table 4.

#### Surface Area

The difference in SA was not significant after Bonferroni correction in 57/68 (83.8%) regions. Unedited images had significantly larger SA in 11/68 (16.2%) regions: the left and right postcentral, left and right precentral, left and right superior parietal, left and right insula, left caudal middle frontal, left superior frontal, and right inferior temporal regions. There were no areas where edited images had significantly larger SA values. The smallest absolute change was observed in the right pars orbitalis (0.26 mm^2^, 0.028%) and the smallest relative change was seen in the right middle temporal gyrus (0.53 mm^2^, 0.015%). The largest absolute change was observed in the right superior parietal region (161.05 mm^2^, 2.55%) and the largest relative change was observed in the right insula (66,41 mm^2^, 2.81%). The SA changes and raw *p*-values for all ROIs are presented in Supplementary Table 5.

The mean change in absolute SA values between the unedited and edited images was 21.21 mm^2^ (0.778%). When we include the direction of the change in the analysis, edited images had lower SA values than unedited images (mean 17.52 mm^2^, 0.643%) and the difference was statistically significant (*p* = 0.000044).

Pearson correlations between edited and unedited images were calculated by ROI, they all were positive and ranged from 0.669 in the left frontal pole to 0.995 in the left supramarginal region. All remained statistically significant after Bonferroni correction. The correlations are presented in Supplementary Table 6.

#### Volume

The difference in volume was not significant after Bonferroni correction in 66/68 (97.1%) regions. Unedited images had significantly larger volumes in 2/68 (2.9%) regions: the left and right insulae. There were no areas where edited images had significantly larger volume values. The smallest absolute change was observed in the left precuneus (0.83 mm^3^, 0.020%) and the smallest relative change was seen in the right superior parietal region (3.58 mm^3^, 0.019%). The largest (both absolute and relative) change was observed in the left insula (189.56 mm^3^, 2.400%). The SA changes and raw *p*-values for all ROIs are presented in Supplementary Table 7.

The mean change in absolute volume values between the unedited and edited images was 31.53 mm^3^ (0.345%). When we include the direction of the change in the analysis, edited images had lower volume values than unedited images (mean 7.98 mm^3^, 0.087%), although the difference was not statistically significant (*p* = 0.175).

Pearson correlations between edited and unedited images were calculated by ROI, they all were positive and ranged from 0.744 in the right frontal pole to 0.995 in the left supramarginal region. All remained statistically significant after Bonferroni correction. The correlations are presented in Supplementary Table 8.

### The ENIGMA and Freeview Quality Control Protocols

Overall, the Freeview quality control protocol was more permissive than the ENIGMA protocol with 7,824 accepted datapoints compared to ENIGMA’s 7,208, out of possible 8,228 (*p* < 0.0001). The largest differences in both directions between Freeview and ENIGMA quality control protocols were found in the left middle temporal gyrus (Freeview 119; ENIGMA 77; difference 42, *p* < 0.0001) and the left precuneus (Freeview 91; ENIGMA 110; difference 19, *p* = 0.0011). The worst quality areas (measured by total datapoints across both protocols) were the right postcentral gyrus and the right middle temporal gyrus with 187 and 188 (out of possible 242) valid datapoints, respectively. The number of included datapoints per ROI is presented in Supplementary Table 2. The number of subjects that passed the protocols with no ROI exclusions was relatively low: three for the Freeview volumetric protocol, 22 for the Freeview CT protocol, and three for the ENIGMA protocol (15 passes for the external and 25 passes for the internal view; notably, the internal was rated as “pass” if it did not result in additional exclusions when viewed after the external view, and therefore the number of passes is overestimated).

### ENIGMA Exclusion Differences Between Edited and Unedited Images

The sample size for this analysis was 61 participants, in total 4,148 ROIs per hemisphere. In the left hemisphere, 238 edited and 318 unedited ROIs were excluded (*p* = 0.0003). In the right hemisphere, 215 edited and 319 unedited ROIs were excluded (*p* < 0.0001). In total, 453 edited and 637 unedited ROIs were excluded (*p* < 0.0001).

Among the edited images, there were 10 that passed the external view without any ROI exclusions (unedited 5, *p* = 0.17), and 13 that passed the internal view (unedited 3, *p* = 0.0073).

**FIGURE 8:**
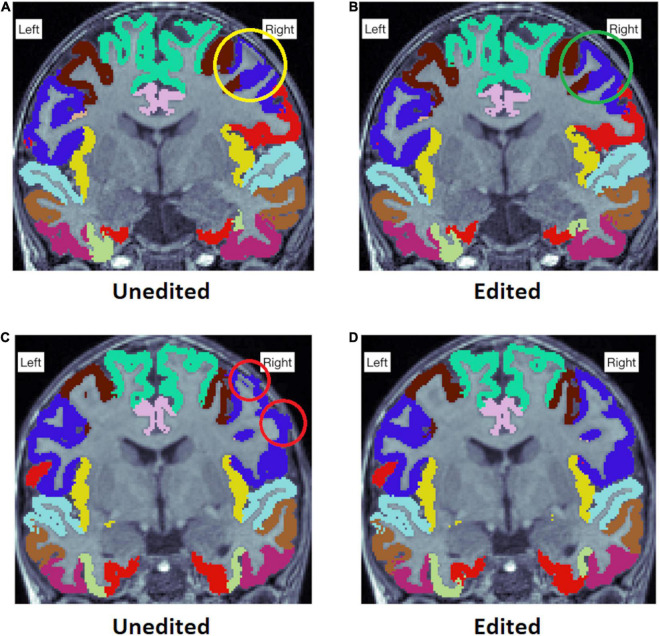
**(A)** Shows an error in the right precentral gyrus, where the cortex is too thin (yellow circle). **(B)** is the edited image of the same participant, and the error is no longer visible in the region (green circle). In addition, **(C)** Shows the right precentral gyrus extending into the skull. **(D)** Shows the edited image of the same participant, where this error is no longer present. Notable, the right precentral gyrus is a region where significant differences between edited and unedited images were observed in cortical thickness and surface area values.

Some typical examples of the differences between edited and unedited images in the ENIGMA internal view are presented in Figure 8.

### Sex Differences

More extensive results are presented in Supplementary Material, Data Sheet 3.

#### Cortical Thickness

In the quality-controlled sample, there were 16/68 ROIs with significant differences (*p* < 0.05) between girls and boys (28/68 in the sample with no quality control). For all regions with significant differences, girls had higher CT values than boys (in both samples).

Regions where a difference was found only in the quality-controlled sample: the right inferior parietal region.

Regions where a difference was found only in the sample with no quality control: the left cuneus, left inferior temporal, left lingual, left postcentral, left rostral anterior cingulate, left superior frontal, left superior temporal, left supramarginal, right cuneus, right lingual, right superior frontal, right superior parietal, and right superior temporal regions.

#### Surface Area

In the quality-controlled sample, there were 57/68 ROIs with significant differences (*p* < 0.05) between girls and boys (61/68 in the sample with no quality control). For all regions with significant differences, boys had higher SA values than girls (in both samples).

There were no regions where a difference was found only in the quality-controlled sample.

Regions where a difference was found only in the sample with no quality control: the left caudal middle frontal, left paracentral, right caudal anterior cingulate, and right superior temporal regions.

#### Volume

In the quality-controlled sample, there were 42/68 ROIs with significant differences (*p* < 0.05) between girls and boys (39/68 in the sample with no quality control). For all regions with significant differences, boys had higher volume values than girls (in both samples).

Regions where a difference was found only in the quality-controlled sample: the left fusiform, left inferior temporal, left middle temporal, left pars opercularis, and right lingual regions.

Regions where a difference was found only in the sample with no quality control: the left posterior cingulate and left superior parietal regions.

### Structural Asymmetry

More extensive results are presented in Supplementary Material, Data Sheet 3.

#### Cortical Thickness

In the quality-controlled sample, there were 18/34 ROIs with significant differences (*p* < 0.05) between the two hemispheres (left thicker in 8, right thicker in 10). In the sample with no quality control, there were 19/34 ROIs with significant differences between the two hemispheres (left thicker in 8, right thicker in 11).

Regions where a difference was found only in the quality-controlled sample: the paracentral, precuneus, and frontal pole regions.

Regions where a difference was found only in the sample with no quality control: the inferior parietal, inferior temporal, middle temporal, and transverse temporal regions.

#### Surface Area

In the quality-controlled sample, there were 28/34 ROIs with significant differences (*p* < 0.05) between the two hemispheres (left larger in 14, right larger in 14). In the sample with no quality control, there were 30/34 ROIs with significant differences between the two hemispheres (left larger in 15, right larger in 15).

Regions where a difference was found only in the quality-controlled sample: the superior parietal region.

Regions where a difference was found only in the sample with no quality control: the medial orbitofrontal, postcentral, and temporal pole regions.

#### Volume

In the quality-controlled sample, there were 30/34 ROIs with significant differences (*p* < 0.05) between the two hemispheres (left larger in 15, right larger in 15). In the sample with no quality control, there were 32/34 ROIs with significant differences between the two hemispheres (left larger in 17, right larger in 15).

There were no regions where a difference was found only in the quality-controlled sample.

Regions where a difference was found only in the sample with no quality control: the entorhinal and inferior temporal regions.

## Discussion

In this article, we described the semiautomated segmentation procedure we used for image processing in detail. While this work relied heavily on existing guidelines by FreeSurfer and the ENIGMA consortium, we believe this article is of help for investigators that are new to pediatric neuroimaging. We add to the existing literature by assessing the effects of our manual edits on CT values, reporting the agreement between FreeSurfer and CAT12, and comparing the FreeSurfer’s standard recon-all to other optional flags.

The manual edits had relatively minor effects on the CT values, less than 2% in all regions [comparable with earlier results by McCarthy et al. (2015)], however it should be noted that the larger effects (such as 0.05 mm in the right caudal middle frontal) are bigger than the yearly change in CT in children [as estimated from figures in Walhovd et al. (2016) and Botdorf and Riggins (2018)]. Importantly, consistent bias in the absolute values may not be an issue when examining longitudinal data, as the values can be scaled to only account for the relative value compared to group average. However, as this change represents measurement error due to artifact in the image as opposed to real difference in cortical thickness, reducing this variability should bring the results closer to the true values, whether scaled or absolute. Edited images had larger CT values in most cases where significant differences were seen. This is not surprising, as most of the editing time is spent correcting small errors in the WM–GM border, and fixing these errors typically “thickens” the cortex (as can be seen in Figure 3). Errors where pia extends too far into dura or cerebrospinal fluid also exist, but naturally only occur in areas next those tissue types. Therefore, they occur repeatedly in the same regions, canceling out some of the bias caused be them. These errors are typically located in the superior parts of frontal and parietal lobes, in regions such as the rostral and caudal middle frontal, superior frontal, superior parietal, precentral, and postcentral gyri. These are the same regions where most of the errors in the WM–GM border are seen. Furthermore, the errors are quite reliably approximately one to two voxels in thickness (e.g., Supplementary Figure 3), while WM–GM border errors can be greater in magnitude and occur anywhere in the brain (e.g., Figure 5). Furthermore, it is crucial to note that the errors in pial surface mainly affect CT estimates SA is often measured from WM–GM border and is therefore unaffected by the pial errors (Winkler et al., 2012), and volumetric segmentation needs to be assessed separately from surfaces. Thinner cortex in edited images was seen in only two regions. In case of the right rostral anterior cingulate, there are some arteries adjacent to it, and erasing them may have had a thinning effect on cortical thickness (Supplementary Figure 18). However, the reason for the apparent thinning of the superior temporal region is unclear.

Similarly, the effects of edits on SA values were relatively minor, less than 3% in all areas and less than 1% on average. A previous study found no significant differences in SA between edited and unedited images (McCarthy et al., 2015). Our results are similar in the sense that differences were not seen in most regions. However, there were a few exceptions, notably including some of the areas with more motion artifact and subsequently more edits, such as the pre- and postcentral gyri as well as the superior parietal region. Where differences were seen, edited images always had smaller SA values. This was to be expected, considering the nature of our edits. FreeSurfer measures the surface area from the WM–GM border, a structure that most of our edits affect. Figure 3 depicts a typical edit made to the WM mask, which corrects an erroneous “fold” in the WM–GM border, thus making SA in that region smaller. The effects for volume were minor, and previous research suggests that the effects are small enough to not affect results when examining correlations between brain volume and neurocognitive tasks (Waters et al., 2019).

The manual editing procedures in many of the previous studies focusing on manual edits (McCarthy et al., 2015; Beelen et al., 2020; Ross et al., 2021) all roughly resemble the FreeSurfer instructions for manual editing and quality control. Ross et al. (2021) focused on volumes in certain ROIs, the amygdala, the hippocampus, the anterior cingulate cortex, and the temporal lobe, in a sample aged 11–17 years. Average manual editing time was 9.5 h, which is a very long time compared to our visual quality control and editing protocol of circa 2 h. They did edit pial errors (such as the one presented in Figure 2C and Supplementary Figure 4), which could explain a large part of this difference in the time requirement. McCarthy et al. (2015) examined the effects of manual edits on CT, SA, and WM volumes in a sample of young adults. They also included pial and control point edits in addition to WM mask edits. In the 3 Tesla images, there were no differences between edited and unedited groups in SA or WM volume. There were a few areas with differences in CT, and the areas involved in both our and their studies (including those that approached significance in their study) were the inferior temporal, superior frontal, precentral, and caudal middle frontal regions. Waters et al. (2019) focused on effects of pial error correction on volumes in a large sample of adults. Beelen et al. (2020) studied the effects of manual editing on six bilateral ROIs (the fusiform, pars opercularis, inferior parietal as well as inferior, middle, and superior temporal regions) in 5–6-years-old children (*n* = 56). Edited images had higher SA and lower CT, but the difference was consistent, and therefore the group comparison results were similar with either data set. Although at a glance these results seem to be directly opposed to ours, the choice of 12 ROIs (when looking at hemispheres separately) should be considered. In our data, edited images had lower CT in 7/12 regions (right superior temporal statistically significant, edited lower CT than unedited, both inferior temporal gyri were significant in the opposite direction), and higher SA in 2/12 (only the right inferior temporal gyrus statistically significant, edited lower SA than unedited). Overall, the inferior temporal gyrus was the main difference between our results, being significant in the opposite direction than expected based on the results by Beelen et al. Notably, the lack of pial edits in our protocol and an emphasis on fixing errors where the WM–GM border extends too far into the cortex could explain why the CT results differ. The reason why our edits make SA smaller rather than larger was discussed earlier, while the reason for the opposite finding might be related to abundant use of control points, however this is speculative. In all these studies, the main conclusion (regarding the manual editing) was that it did not significantly affect results/conclusions, even if there were significant differences in the CT, SA, or volume values. In our study, we cannot assess the effects of these edits on the results of some non-neuroimaging test. However, we highlight a benefit rarely discussed in earlier literature. The manual edits improved image quality, allowing for more ROIs in more participants to pass the visual quality assessment, in effect rising the number of usable datapoints within the sample.

We also examined the effects of our manual editing and quality control protocol on the results regarding sex differences and structural asymmetry. We observed differences in the number of regions with sex differences, wherein there were more significant findings without quality control (especially with CT). Notably, the quality control protocol leads to exclusion of some ROIs in some participants, and therefore some of this effect may be due to decreased sample size. However, this effect was also seen in regions with few exclusions, such as the left caudal middle frontal, left paracentral, and left posterior cingulate regions, suggesting that it is not the only cause for this difference, and our results imply that the lack of quality control may lead to some false positive findings. Furthermore, we found some regions that only showed sex differences after the quality control protocol. These differences were mostly seen in volumes. Notably, these regions include the left inferior and middle temporal gyri, regions that were quite often excluded due to topological errors. This suggests that the errors in automated segmentation in this area may be large enough to cause false negative findings, unless addressed either by manually fixing the errors, which can be an arduous and time-consuming process, or excluding the erroneous cases from analyses. In conclusion, manual editing and quality control can affect the results in group comparisons or examinations of structural asymmetry of brain structure.

Our conclusion seems to differ from earlier research, that suggests that additional manual editing is not necessary (McCarthy et al., 2015; Waters et al., 2019; Beelen et al., 2020; Ross et al., 2021). Notably, most of these studies were done on adolescents (Ross et al., 2021) or adults (McCarthy et al., 2015; Waters et al., 2019). Older participants move less during the scan, leading to less errors in segmentation. Understandably, editing has less utility when images are already of higher quality. Beelen et al. (2020) had a similar age group to our study. They examined CT and SA in 12 ROIs, and found two cases with differences between fully automated and edited versions (SA in right inferior temporal gyrus and CT in the pars opercularis). Two out of 24 ROIs (12 CT, 12 SA) is 8.3%, compared to our 13.2% (18/136, only CT and SA measurements of sex differences), suggesting that our findings are not radically different from earlier research. Of course, this comparison cannot be made directly, considering the differences group comparison and editing protocols.

For inclusion and exclusion criteria of images, we propose that there are two major approaches: micro and macro scale approach. In the micro scale assessment, we could find the errors as described in the methods section and score the image based on their number and size. However, this approach has multiple challenges. Firstly, there are many errors that do not warrant exclusion of the ROI in question, e.g., small errors in the WM–GM border (demonstrated in Figure 3A). In some cases, these types of errors were abundant despite rarely meeting the exclusion criteria. How should the number of these errors be calculated and what weight should they be given compared to larger errors? Secondly, in many cases it is not obvious whether there is an error in the slice or not (one typical case is an image with poor WM–GM contrast). If we were to count errors by the number of slices with a certain type of error, differences between raters could lead to large differences in these cases. These could be viewed by multiple expert raters and discussed, however that would be very time consuming and arduous, while one of the main goals of semiautomated segmentation programs is to make the process as fast and easy as possible. Thirdly, quality control protocols are often described on a general level in scientific studies (El Marroun et al., 2016; Kamson et al., 2016; Barnes-Davis et al., 2020; Boutzoukas et al., 2020), and therefore there is no commonly accepted way to assess all the errors in the automated segmentation.

In contrast, in the macro scale assessment, the rater can quickly look at the brain image, and assess the amount of motion artifact (i.e., motion as marked by “waves” or “concentric rings” in the typical MPRAGE image) and the clarity of the WM–GM border. In borderline cases, the image can be segmented and then assessed for major segmentation errors, such as ubiquitous errors in the WM–GM border or large unsegmented areas. One key challenge with this approach is the lack of objective criteria, as these types of errors are very difficult to quantify or to describe in articles or instructions. However, the expert rater makes this same assessment for all images and can learn to quickly exclude the images that are of significantly poorer quality than others, and therefore a high internal reliability should be attainable. Furthermore, as this type of assessment can be made quickly, unclear cases can be verified by other raters with little additional time commitment. Considering the pros and cons of both approaches, we decided to use macro scale assessment for exclusion of whole images. Furthermore, we decided to apply it on a dichotomous pass or fail scale and skip further quality classification. One possible downside is the loss of subcategories in the accepted sample, since image quality can be included in regression analyses (Shaw et al., 2007). However, in our study, we perform a rigorous quality control protocol that rates image quality on a level of single ROIs, and therefore all datapoints in the final sample are of high quality. Consequently, we believe a further categorization based on overall image quality would not add significant value in this case.

We decided to apply the widely used and accepted ENIGMA quality control protocol (Thompson et al., 2020) to support decisions on inclusion and exclusion of ROIs. It has previously been implemented for both adults (Thompson et al., 2020) and children (Boedhoe et al., 2018; Hoogman et al., 2019). The internal view of ENIGMA protocol gives 16 slices with color coded segmented ROIs. This gives a good overall view of the brain, but it does not allow for exploration of unclear cases, and some errors can be completely missed if they are not located in the slices presented by ENIGMA. To explore this issue, we presented our own Freeview quality control protocol, and as a result of using Freeview for slice-by-slice assessment of the brain (e.g., the errors seen in Figure 5) it was more sensitive to certain types of small errors than the ENIGMA protocol. However, this protocol was not implemented for the final analyses due to the challenges discussed earlier in this article, such as the large number of minor errors and the lack of consensus on how to treat them. For example, the areas that were the most commonly excluded from the volumetric analyses in the Freeview protocol were the left lingual gyrus and left precuneus (Supplementary Table 4). Both regions are adjacent to the posterior tip of the lateral ventricle and were therefore often excluded due to a few mislabeled voxels. Overall, the Freeview protocol was more permissive than the ENIGMA protocol. One major reason for this is that it lacks the external view that ENIGMA has and therefore cannot assess errors in borders between ROIs. Therefore, even if the Freeview quality control protocol were implemented, it would have to be implemented together with the ENIGMA protocol. However, future studies should explore the utility of slice-by-slice assessment of the Freeview image, as some of the errors found via that method may be large enough to warrant exclusion from statistical analyses.

The key practical benefit in our manual edits protocol is the relative ease of application. Errors caused by remaining parts of the skull are very clear and easy to fix (Figure 2A). Fixing arteries by erasing all voxels between certain intensity values requires practically no decision-making during execution. Edits in the WM–GM border take the most time and require the most expertise. However, as the edits are followed by another automated recon-all protocol, that considers the human input in calculations, the editor cannot decide the exact delineation between WM and GM, and therefore cannot make errors that would mandate editing the image again from scratch. Such errors were possible while editing the SSS (please see Supplementary Material, Data Sheet 2, pages 10–14), however SSS edits were stopped after an interim analysis. While it could be argued that the effect on CT values is not worth the time that manual edits require, we believe that systematic manual edits protocol has an additional benefit: It maximizes the chance to find and fix errors that would lead to exclusion of the ROI in question, therefore increasing the number of valid datapoints in the final sample.

There is an increased need for manual edits and diligent quality control in pediatric imaging. Children move more than adults during scans (Blumenthal et al., 2002; Poldrack et al., 2002; Theys et al., 2014), and therefore there is an increase in ringing and blurring artifacts in images. The artifact can lead to unreliable cortical parcellations, and the errors must be noted and fixed when possible. While previous studies suggest that the effects of edits on brain metrics may be small enough to not affect group comparisons (McCarthy et al., 2015; Waters et al., 2019), we observed an increased inclusion rate in the ENIGMA quality control protocol, which effectively increased our potentially available sample size. Furthermore, the choice of automated segmentation tool can be very influential. For example, in adults, FreeSurfer and CAT12 have shown good agreement (Seiger et al., 2018; Masouleh et al., 2020), however in our sample the agreement was relatively poor. CAT12 often overestimated CT compared to FreeSurfer, but the opposite was also true a significant number of cases, showing that the disagreement was not systematic. Therefore, the results cannot be reliably compared in this population. Please see Supplementary Material (Data Sheet 2, pages 19–25, CAT12) for more discussion. Furthermore, a child’s brain undergoes non-linear regional developments through its development, which means it cannot simply be considered a slightly smaller adult brain (Wilke et al., 2003; Phan et al., 2018b).

Certain typical errors can appear in unedited pediatric FreeSurfer images when applying adult templates, as presented in a review by Phan et al. (2018b). The review presents the errors in pial border that were often seen in the temporal regions in our sample. On the other hand, cerebellum was mislabeled only once in our final sample (Supplementary Figure 19). Additionally, we did observe erroneous automatic segmentation in the subcortical regions, and we are preparing an article regarding the manual segmentation of these areas (Lidauer et al., 2021). Similarly, pediatric atlases have their own challenges. One key challenge with age specific atlases is that the required specificity regarding the age range is unclear. Multiple age specific atlases, some of them freely available, have been created for neonates and infants (Kuklisova-Murgasova et al., 2011; Shi et al., 2011), and they have shown good agreement with the “gold standard” manual segmentation (Oishi et al., 2011; Serag et al., 2012). The age ranges in these atlases may be very specific, e.g., covering the preterm neonates aged between 29 and 44 gestational weeks (Kuklisova-Murgasova et al., 2011). In comparison, in older pediatric populations, the age ranges may be a few years or even more than 10 years (Wilke et al., 2003; Fonov et al., 2011). In addition, pediatric atlases have the challenges that atlases have in general, such as the specificity of the group (e.g., a certain disease) and ROIs. Considering the multitude of different options in pediatric atlases, their use may complicate comparisons between studies. Therefore, we decided to use the standard adult atlas with appropriate quality control measures to counter the challenges this approach has. We were generally satisfied with the cortical segmentation results, but it remains an important venue to develop and validate implemented in mainstream software such as FreeSurfer (de Macedo Rodrigues et al., 2015; Zöllei et al., 2020). In FreeSurfer, adult template is used for creating the volumetric segmentation (aseg.presurf.mgz). The aseg is also partially used when initializing the surfaces; after that, the surfaces are placed based on following intensity gradients which are independent of any atlas.

One of the key limitations in our study is the reliance on visual assessment in the quality control. Considering the inherent arbitrariness of the visual assessment of motion artifact, there is interest in developing automated quality assessment algorithms (White et al., 2018). An automated, objective estimate of the severity of the motion might allow us to set universal standards on the different categories of motion severity. There are some challenging key questions that would need to be resolved before the creation of a system to correct for motion artifact: (1) how much different levels of motion affect different aspects of brain morphology (Blumenthal et al. provide estimates of the decrease in volume in a seemingly non-linear manner, as the change from moderate to severe artifact causes a major drop in volumes compared to the other classifications); and (2) are the effects similar throughout the brain or are there significant regional differences. Considering these challenges, more research is needed before the effects of motion artifact can be accounted for automatically. Another approach is to lessen motion artifact by adding prospective motion correction (PMC) to the T1-weighted imaging sequence (Ai et al., 2021). The benefit is clearest in images with a lot of motion artifact, while the cost is poorer performance in some quality control measures such as signal to noise ratio compared to a MPRAGE sequence without PMC (Ai et al., 2021). While implementation of PMC could improve the quality of our data, it would not remove the need a quality control protocol such as the one we presented in this article, and therefore the existence of this alternative imaging sequence does not impact our main findings. Although we opted for a quality control protocol that performs visual quality control on a level of individual ROIs, investigators may additionally benefit from using custom software to detect potentially low quality data (Klapwijk et al., 2019).

## Conclusion

There is no single “gold standard” processing method for pediatric images, and thus there is methodological variation between different studies. Pediatric images are inherently more susceptible for segmentation errors than adult images. This highlights the need for rigorous quality control to ensure high quality data. We believe that detailed method descriptions are crucial for maximal transparency that helps comparisons between studies.

In this article we have described in detail the semiautomated segmentation protocol used in the FinnBrain Neuroimaging Lab, including manual edits and the implementation of the ENIGMA quality control protocol. We decided to use the standard recon-all without optional registration flags, as they did not provide additional benefits. Furthermore, we observed a surprisingly poor agreement between FreeSurfer and CAT12 output. Our semiautomated segmentation protocol provides means to assure the high quality of pediatric neuroimaging data and could help investigators working with similar data sets.

## Data Availability Statement

The datasets presented in this article are not readily available because research data cannot be shared publicly. The ethics committee decision and local legislation do not allow the open sharing of neuroimaging data. Requests to access the datasets should be directed to EP, (elmo.p.pulli@utu.fi), and are subject to Finnish legislation and formal local procedures for data sharing.

## Ethics Statement

The studies involving human participants were reviewed and approved by Joint Ethics Committee of the University of Turku and the Hospital District of Southwest Finland. Written informed consent to participate in this study was provided by the participants’ legal guardian/next of kin.

## Author Contributions

EP, ESi, and JT conceptualized the work and drafted initial version of the manuscript. EP, ESi, VK, AC, and ESa collected the data. EP and ESi manually edited the data. EP performed the final quality check on the data and was supervised by JT. HK and LK started the FinnBrain Birth Cohort and provided the infrastructure for the studies. All authors critically revised the manuscript and accepted it in its final form.

## Conflict of Interest

The authors declare that the research was conducted in the absence of any commercial or financial relationships that could be construed as a potential conflict of interest.

## Publisher’s Note

All claims expressed in this article are solely those of the authors and do not necessarily represent those of their affiliated organizations, or those of the publisher, the editors and the reviewers. Any product that may be evaluated in this article, or claim that may be made by its manufacturer, is not guaranteed or endorsed by the publisher.

## Footnotes

1
http://surfer.nmr.mgh.harvard.edu/

2
http://enigma.ini.usc.edu/

3
https://fil.ion.ucl.ac.uk/spm

4
http://www.neuro.uni-jena.de/cat/

5
www.finnbrain.fi

6 https://surfer.nmr.mgh.harvard.edu/fswiki/FreeSurferWiki?action=AttachFile&do=get&target=FreeSurfer_Suggested_Morphometry_Protocols.pdf, at the time of writing

7
http://surfer.nmr.mgh.harvard.edu/

8
https://surfer.nmr.mgh.harvard.edu/fswiki/FsTutorial/

9
http://enigma.ini.usc.edu

10
https://www.r-project.org/

11
https://jasp-stats.org/
