# Supplementary material for: Feasibility of FreeSurfer Processing for T1-Weighted Brain Images of 5-Year-Olds: Semiautomated Protocol of FinnBrain Neuroimaging Lab
Source: Front Neurosci. 2022 May 2;16:874062. doi: 10.3389/fnins.2022.874062 (PMC9108497; doi:10.3389/fnins.2022.874062)
Supplement: Supplementary file 1 [file Data_Sheet_1.docx]

Feasibility of FreeSurfer processing for T1-weighted brain images of 5-year-olds: semiautomated protocol of FinnBrain Neuroimaging Lab

Supplementary Figures


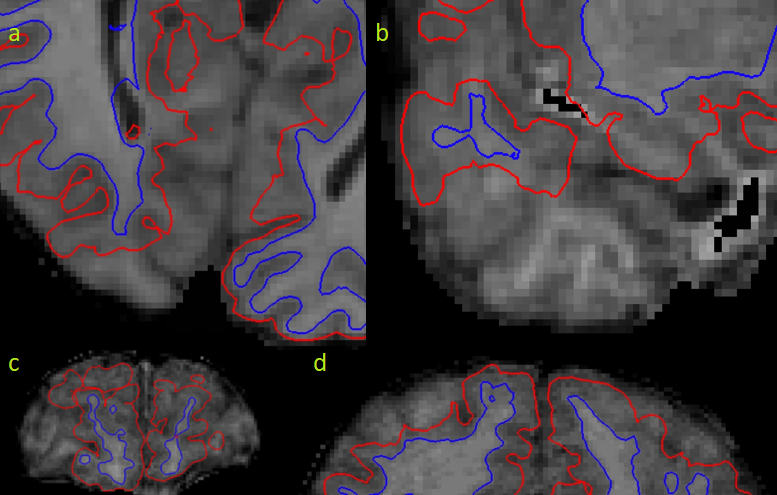


**Supplementary Figure 1**. The figure demonstrates large unsegmented areas in occipital **(A)**, temporal **(B)**, and frontal **(C)** and **(D)** regions. These errors that leave areas over multiple gyri unsegmented lead to exclusion of the whole image.


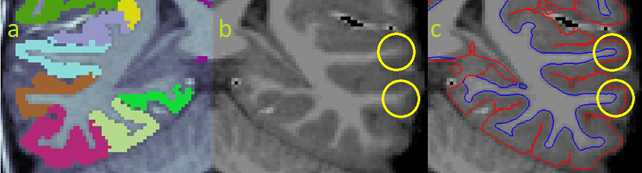


**Supplementary Figure 2.** A presentation of the same slice of the temporal region in the left hemisphere in ENIGMA internal view **(A)** and Freeview without borders **(B)** and with borders **(C)**. There is unsegmented gray matter on the side of the pial border, especially in middle temporal gyrus (brown area), and to a lesser extent in the superior temporal gyrus (light blue area). Meanwhile in Freeview, the border between white and gray matter (blue line) seems accurate based on voxel intensity changes.


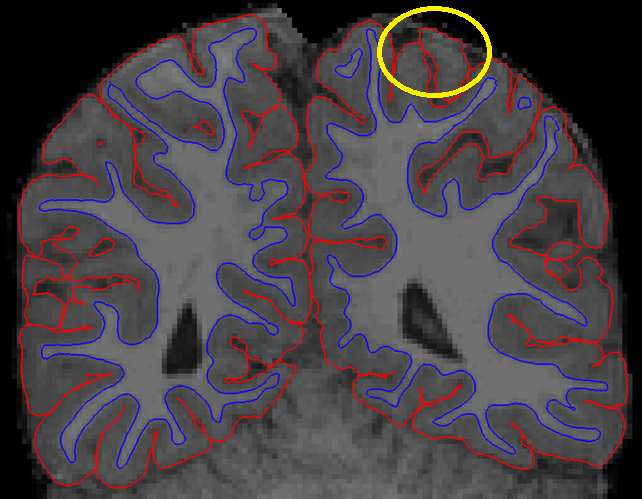


**Supplementary Figure 3.** The pial border extends into meninges (yellow circle). The outmost part of the gyrus seems continuous with the meninges seen on both sides of it. Furthermore, a lower intensity layer can be seen between this area and the rest of the gyrus.


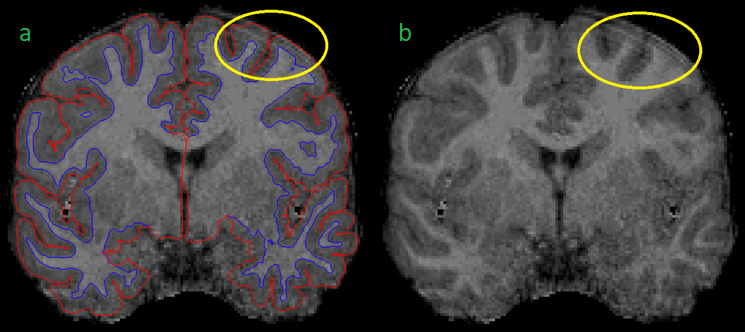


**Supplementary Figure 4. (A)** and **(B)** portray the same slice with **(A)** and without **(B)** visible borders. Especially on the left hemisphere (yellow circles), motion artefact makes it difficult to demarcate the location of the correct pial border.


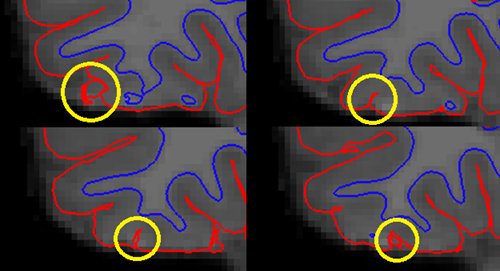


**Supplementary Figure 5.** The figure presents a minor incongruity in the pial border. Four adjacent slices are presented. Although these seem like potential errors in this view, this is simply a result of a 3D surface being presented in a 2D slice. The border seems normal in other planes.


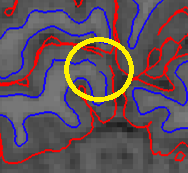


**Supplementary Figure 6.** There is an apparent discontinuation in the border between white and gray matter. We found a few cases like this, and the border was always continuous in other planes. These were considered errors related to 2D presentation of a 3D image and therefore included.


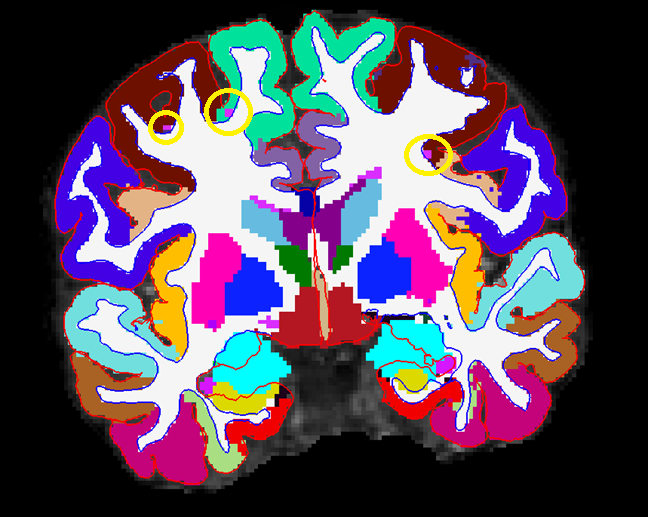


**Supplementary Figure 7.** There are multiple small white matter (WM) hypointensities (purple areas noted by yellow circles). In this case, none affected the border between white and gray matter.


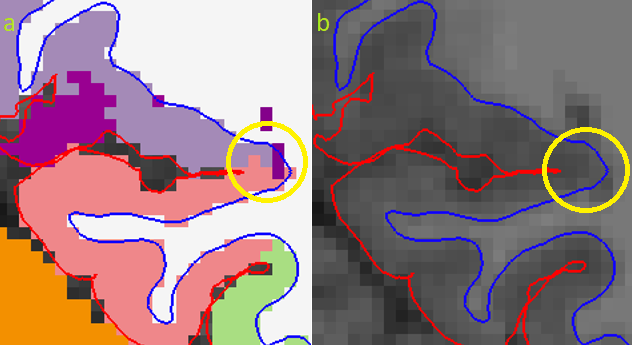


**Supplementary Figure 8. (A)** and **(B)** show a small part of the lateral ventricle between the WM–GM border and the pial border [yellow circle in **(A)**]. However, there is no clear border distortion in the image [yellow circle in **(B)**].


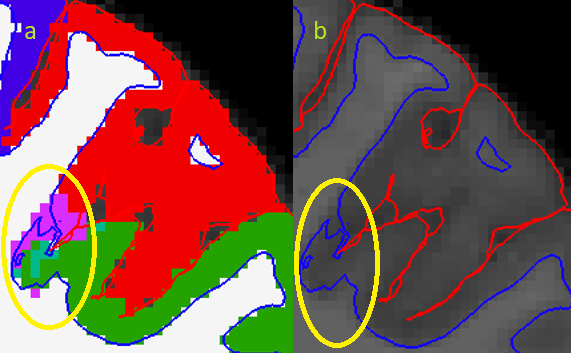


**Supplementary Figure 9. (A)** and **(B)** show a white matter (WM) hypointensity (purple area denoted by the yellow circle) that affects the border between white and gray matter (WM–GM border). As is unfortunately common, it is located at the junction of three different regions of interest (postcentral – red, supramarginal – green, and superior parietal – light blue, in this case), leading to the exclusion of all three in the Freeview quality control protocol.


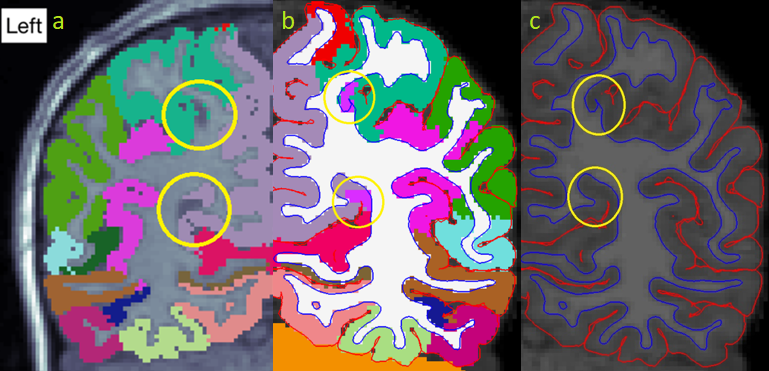


**Supplementary Figure 10.** There is missing cortical segmentation in the ENIGMA internal view [**(A)**, yellow circles]. White matter (WM) hypointensities are seen in the same places in Freeview [**(B)**, yellow circles], while **(C)** shows that these hypointensities do not necessarily equate to errors in the border between white and gray matter. Note that left and right hemispheres swap sides between ENIGMA internal view and Freeview.


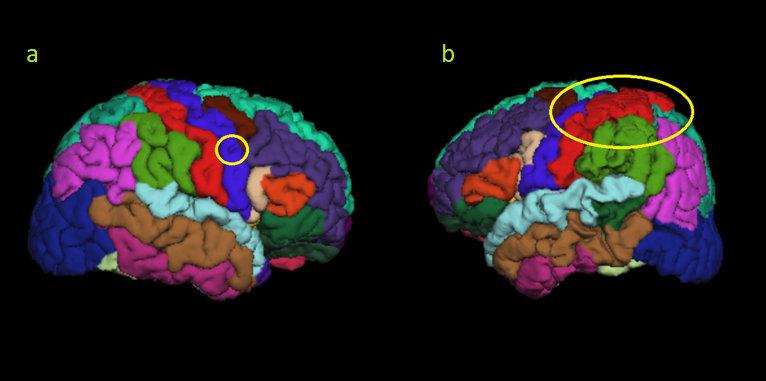


**Supplementary Figure 11.** A presentation of meninge overestimations. “Spikes” in **(A)** and flat areas in **(B)**. According to the ENIGMA quality check protocol instructions, these are typical errors in pre- and postcentral areas. However, these errors were rare in our sample.


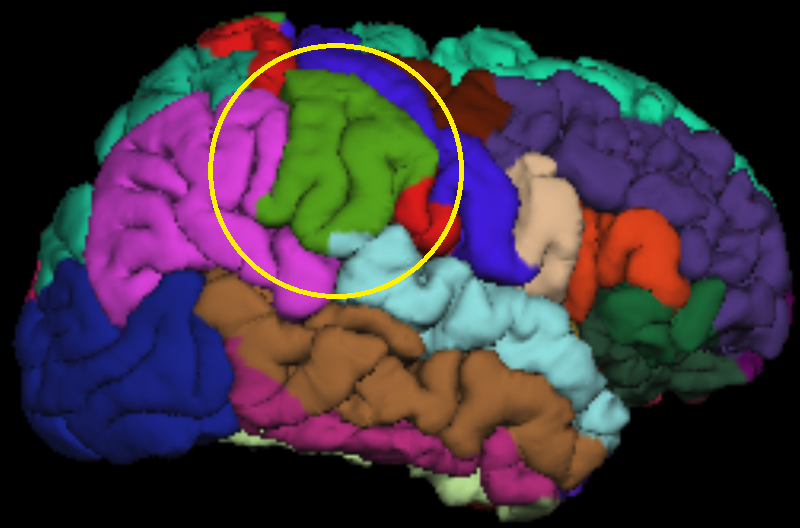


**Supplementary Figure 12.** Supramarginal gyrus overestimation into the postcentral gyrus region.


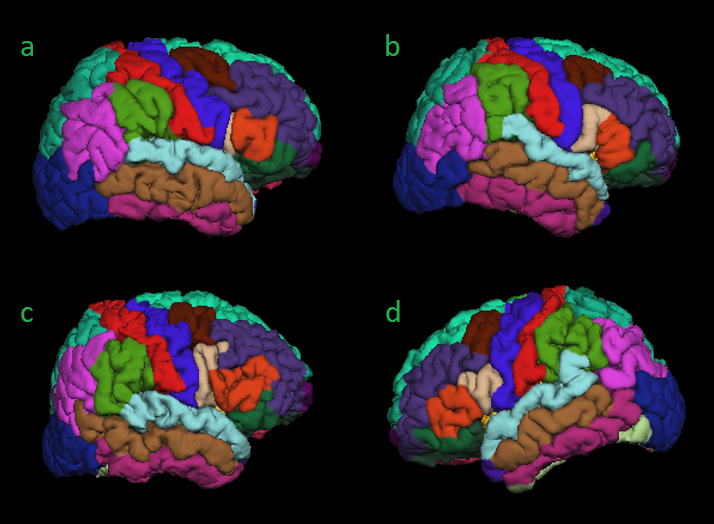


**Supplementary Figure 13.** Sometimes there were errors in the border between the superior temporal gyrus (light blue) and the supramarginal gyrus (green), where the border does not follow sulcal lines **(A)** and **(B)**, the supramarginal gyrus is clearly overestimated in the inferior direction **(C)**, or the supramarginal gyrus is clearly overestimated in the superior direction **(D)**.


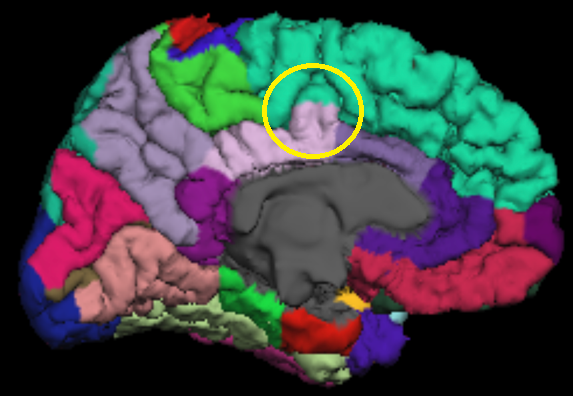


**Supplementary Figure 14.** There is an atypical an error in the border between posterior cingulate and superior frontal.


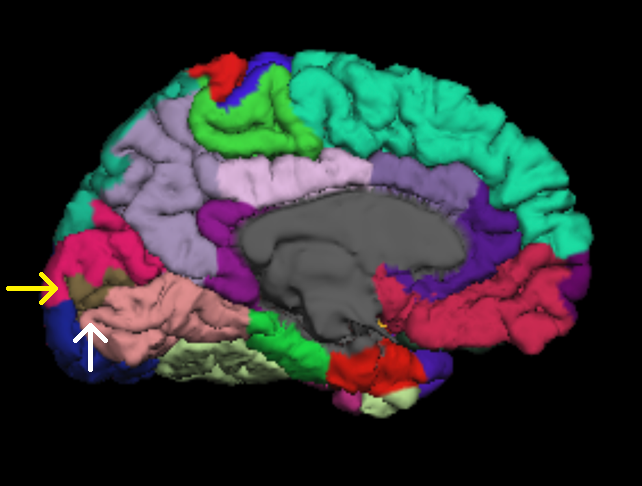


**Supplementary Figure 15.** There is pericalcarine region overestimation into both cuneus (yellow arrow) and lingual (white arrow). All three regions were excluded.


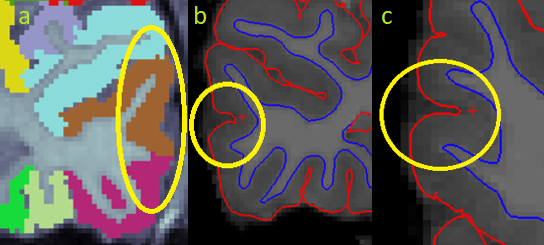


**Supplementary Figure 16. (A)** shows an internal view in ENIGMA, where there might be unsegmented gray matter in the outer border of the right middle temporal region (brown label). **(B)** shows the same area in coronal view in Freeview. **(C)** shows the same region in axial direction [the red cursor is in the same coordinates as in **(B)**]. The border seems normal in the region.


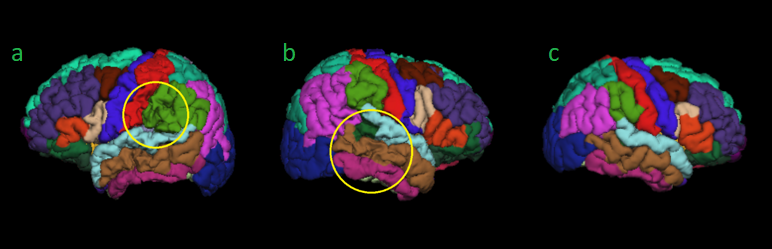


**Supplementary Figure 17. (A)** and **(B)** show areas with erroneous lateral cortical segmentation, that is more reliably observed in the internal view (Figure 4d). **(C)** shows a good quality image for comparison.


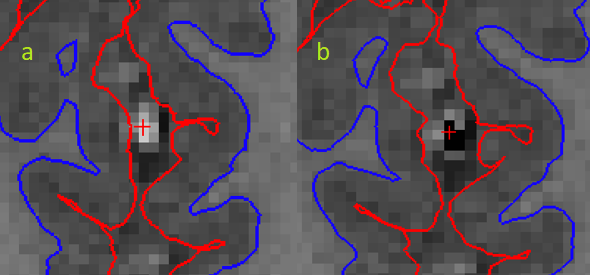


**Supplementary Figure 18.** The rostral anterior cingulate region before **(A)** and after **(B)** edits. The red cursor is in the same voxel in both images. The cortex to the left of the cursor (right hemisphere) appears slightly thinner after edits.


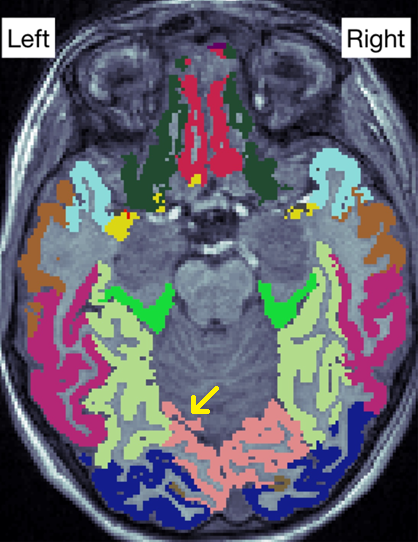


**Supplementary Figure 19.** In some rare cases, parts of the cerebellum were mislabeled as cortex (yellow arrow). This was the only included image with this type of error. Cerebellum edits were not included in our manual edits protocol. Therefore, the left lingual region was excluded in this case.
