# Supplementary material for: Feasibility of FreeSurfer Processing for T1-Weighted Brain Images of 5-Year-Olds: Semiautomated Protocol of FinnBrain Neuroimaging Lab
Source: Front Neurosci. 2022 May 2;16:874062. doi: 10.3389/fnins.2022.874062 (PMC9108497; doi:10.3389/fnins.2022.874062)
Supplement: Supplementary file 2 [file Data_Sheet_2.docx]

# Feasibility of FreeSurfer processing for T1-weighted brain images of 5-year-olds: semiautomated protocol of FinnBrain Neuroimaging Lab

# Supplementary materials

Supplementary Figures, Supplementary Tables, and Supplementary results regarding sex differences and structural asymmetry are in separate files. For other supplementary materials in order of appearance in the manuscript, please see table of contents below.

# Table of contents

[Feasibility of FreeSurfer processing for T1-weighted brain images of 5-year-olds: semiautomated protocol of FinnBrain Neuroimaging Lab 1](#_Toc101268495)

[Supplementary materials 1](#_Toc101268496)

[Table of contents 1](#_Toc101268497)

[FreeSurfer editing 3](#_Toc101268498)

[1. The FreeSurfer ouput in Freeview. 4](#_Toc101268499)

[Remarks about Freeview 4](#_Toc101268500)

[2. Brain mask errors – erase excess voxels from brain mask 6](#_Toc101268501)

[3. White matter mask too large – Erase and fill where appropriate 7](#_Toc101268502)

[Workflow 7](#_Toc101268503)

[4. Arteries mess things up 8](#_Toc101268504)

[5. Restart the recon-all 9](#_Toc101268505)

[Superior sagittal sinus 10](#_Toc101268506)

[Methods 10](#_Toc101268507)

[Manual edits 10](#_Toc101268508)

[Statistics 10](#_Toc101268509)

[Results 10](#_Toc101268510)

[Discussion 10](#_Toc101268511)

[Figures 12](#_Toc101268512)

[Table 14](#_Toc101268513)

[Subcortex 15](#_Toc101268514)

[Methods 15](#_Toc101268515)

[Results 15](#_Toc101268516)

[Figure 16](#_Toc101268517)

[Optional flags 17](#_Toc101268518)

[Methods 17](#_Toc101268519)

[Image processing 17](#_Toc101268520)

[Statistics 17](#_Toc101268521)

[Results 17](#_Toc101268522)

[Table 18](#_Toc101268523)

[CAT12 19](#_Toc101268524)

[Methods 19](#_Toc101268525)

[CAT12 processing 19](#_Toc101268526)

[Statistics 19](#_Toc101268527)

[Results 19](#_Toc101268528)

[Discussion 20](#_Toc101268529)

[Figure 21](#_Toc101268530)

[Tables 22](#_Toc101268531)

[Qoala-T 26](#_Toc101268532)

[Methods 26](#_Toc101268533)

[Statistics 26](#_Toc101268534)

[Results 26](#_Toc101268535)

[Discussion 26](#_Toc101268536)

[Figures 28](#_Toc101268537)

[References 30](#_Toc101268538)

# FreeSurfer editing

These instructions are based on the instructions provided in the FreeSurfer manual (<https://surfer.nmr.mgh.harvard.edu/fswiki/FsTutorial/>).

The instructions were modified by Elmo P. Pulli and Jetro J. Tuulari for use in the FinnBrain Neuroimaging lab and then for the purposes of this article.

## 1. The FreeSurfer ouput in Freeview.

**
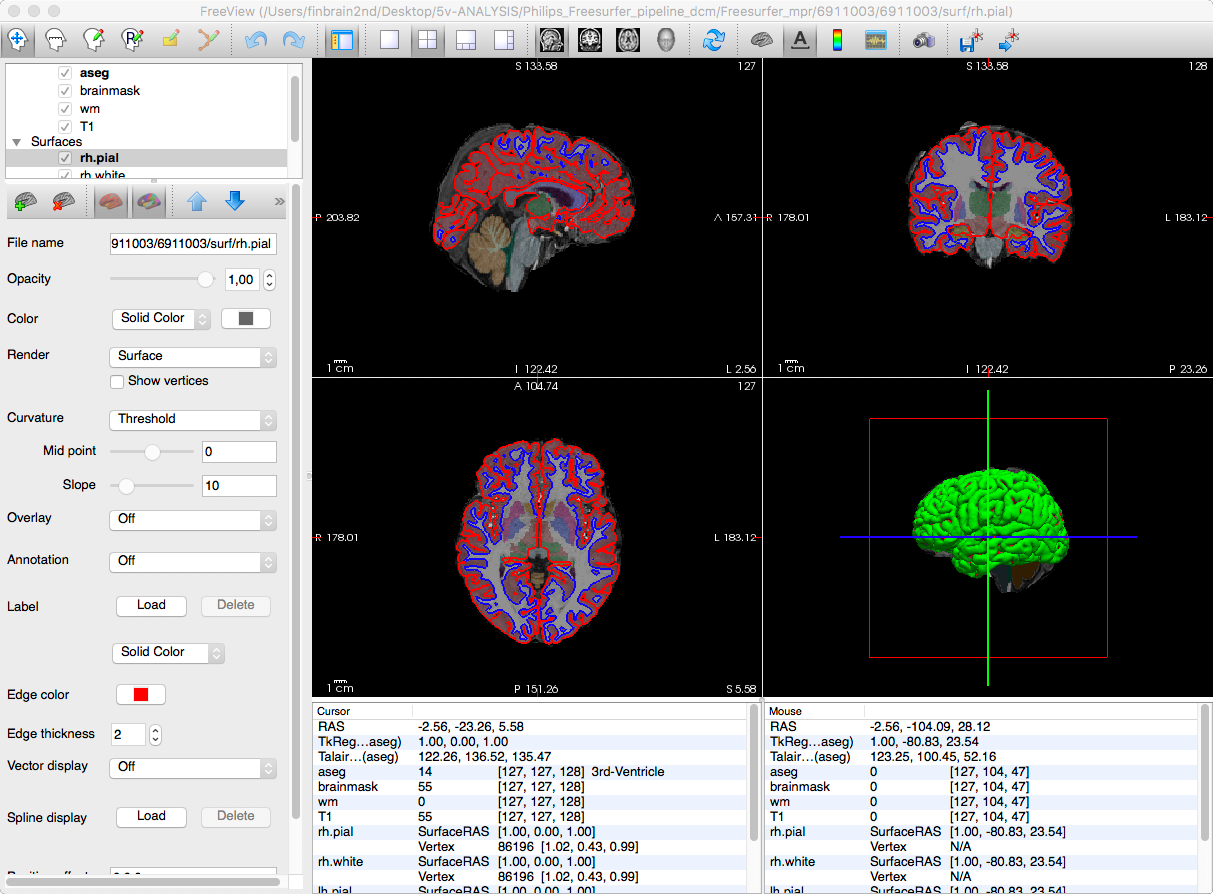
**

**Figure S1.** An example of FreeSurfer output in Freeview.

### Remarks about Freeview

**Keyboard Shortcuts**:

- Alt+a and Alt+s will change the opacity of the layer that is currently highlighted.
- Right click plus mouse movement will zoom in and out.
- Middle mouse button plus movement will let you change the location of the image.
- Fn+Command+Up/Down will let you move through the slices of the selected view.
- Shift+Right click plus mouse movement will let you adjust the contrast of the images.

**There are regions where the surfaces are not intended to be accurate, and this is fine:**

- Areas around the hippocampus and amygdala. The surfaces will not completely include or exclude certain subcortical regions. These inaccuracies can be ignored as subcortical regions are excluded from the cortical measures and subcortical volume is measured by the aseg, not the surfaces.
- Along the midline cut (at the level and below corpus callosum), it is possible to see some overlapping of the surfaces from one hemisphere to another. The medial wall is not included in the cortical measures so this can generally be ignored.

There are some mistakes that appear in most outputs and they often need to be manually corrected for. The following chapters will cover these in depth.

## 2. Brain mask errors – erase excess voxels from brain mask

For these edits, make sure the brainmask is highlighted in the top left menu.

Note: remember to save frequently, unsaved work is lost if the software crashes.

FreeSurfer sometimes leaves small parts of the skull to remain within the brain mask. Similarly, the frontotemporal parts may contain pieces of bone and dura.

The skull fragments that cause segmentation errors should be deleted. Open the reckon editing module.

- Left mouse button will let you paint.
- Shift + Left mouse button will let you erase.

**Note**: You should be extremely careful with deleting the voxels. No brain voxels are to be deleted!

**Note**: FreeSurfer grey matter segmentations and surfaces appear right in most places, but the manual cleaning of the brain mask is a necessity as the cleaned images are used in other software of the sMRI pipeline set.

## 3. White matter mask too large – Erase and fill where appropriate

For these edits, make sure that “wm” (WM = white matter) is highlighted in the top left menu.

Go through the slices systematically and look for areas where the white matter surface has failed.

Then deselect aseg and brainmask from the tick boxes.

Change the color map to Jet from the left panel of Freeview.

What you see now are the surfaces as before, but only the WM volume mask is displayed. This is the surface that FreeSurfer uses to initialize the segmentation and surface reconstruction.

These should be modified to fix the errors. Open the reckon editing module.

- Left mouse button will let you paint.
- Shift + Left mouse button will let you erase.

Note, that you will see the same errors that you might have fixed when you scroll through the data from different views.

### Workflow

1. Look for excess white WM, Go through the coronal, axial, and sagittal slices (preferably in that order).
2. When you find extra WM enter the color code “Jet”, see above, and fix the “wm” surface.
3. Remember to think 3D, all the mistakes you see in one spot most likely start from few slices to either direction, i.e., always look for more than 1 slice to correct.

## 4. Arteries mess things up

Arteries can be easily seen in T1 volumes. They have high contrast values and thus appear as white. It is not uncommon to find that arteries are segmented as a part of white matter (anterior temporal areas) and / or make the estimates of pial surface inaccurate (insular regions).

Getting rid of the arteries.

- Have brainmask highlighted. Go to reckon edit view.
- Select Advanced erasing tab.
- Click “Only erase voxels in the range of”.
- Input range: 130-190.

Go through the arteries by painting over them. They will turn black, meaning that they will have low intensity in the subsequent processing.

## 5. Restart the recon-all

After the edits, the recon-all is run again, this time accounting for the edits. Note that following manual edits, the “-i” flag for input volume is not used: <https://surfer.nmr.mgh.harvard.edu/fswiki/FsTutorial/TroubleshootingData>

# Superior sagittal sinus

## Methods

### Manual edits

One typical error was that parts of the superior sagittal sinus (SSS) were included within the pial border (Figure S2; Figure S3 shows the same subject in ENIGMA external). These were originally corrected based on visual assessment, erroring to the side of leaving excess sinus. However, due to partial volume effects, a voxel that is clearly SSS in one plane can be partially brain tissue in another, which means it is easy to make errors that necessitate editing the image again from the beginning. Furthermore, the border between cerebrospinal fluid (CSF) and gray matter (GM) is sometimes very poorly visible, and therefore this step includes a high risk of human error and presumably has a lot of inter- and intra-rater variability. Considering these challenges, SSS editing was eventually stopped after an interim assessment as it was an arduous task with little effect on final results (Table S1).

### Statistics

To assess the effect of SSS edits, we divided the sample into two groups: those with 1) SSS corrected, and 2) SSS not corrected. For both groups separately, we calculated the change in cortical thickness (CT) in the regions adjacent to the SSS. These were the superior frontal, precentral, postcentral, paracentral, superior parietal, lateral occipital, precuneus, cuneus, pericalcarine, and lingual regions. Subsequently, we ran an independent samples t-test to compare the difference in CT changes between the two groups.

## Results

We divided the sample into two groups: 1) those with sinus corrected (n = 95), and 2) those with sinus not corrected (n = 26). Subsequently, we compared the changes in CT values between the unedited and edited images. The only significant difference between the groups was observed in the left cuneus (p=0.009), where in group 1, edited images had lower CT values (mean change -0.0132mm, SD 0.0504), and in group 2, edited images had higher CT values (mean change 0.0111mm, SD 0.0288). This difference did not remain statistically significant after Bonferroni correction. All differences can be seen in Table S1.

## Discussion

Editing the SSS was an arduous and time-consuming process. Erasing a brain voxel from the brainmask volume means that the voxel is permanently lost. Due to partial volume effects, a voxel that is clearly SSS in one plane can be partially brain tissue in another, which means it is easy to make errors that necessitate editing the image again from the beginning. Furthermore, in many cases there was a clear error in an area adjacent to the SSS, but the correct border was impossible to demarcate and therefore the error could not be reliable fixed. The editor had to carefully consider, which errors could be safely fixed, and as a result this step was very time consuming and prone to human error. Additionally, we noticed that SSS edits had no significant effects on CT values (Table S1), meaning it was inefficient in terms of used time. Furthermore, while the automated segmentation is imperfect, it is reliably repeatable and not susceptible to rater dependent biases. As a result of these complications, we decided to stop editing the SSS area. We acknowledge that the small size of the group without sinus corrections may affect the reliability of these results.

## Figures


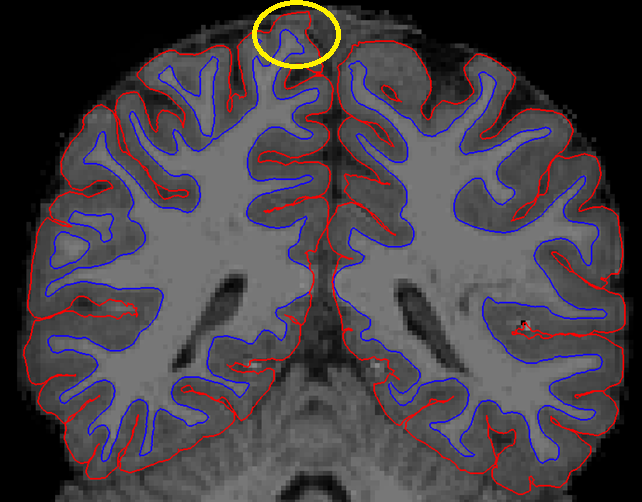


**Figure S2.** The pial border may extend into the superior sagittal sinus (SSS).


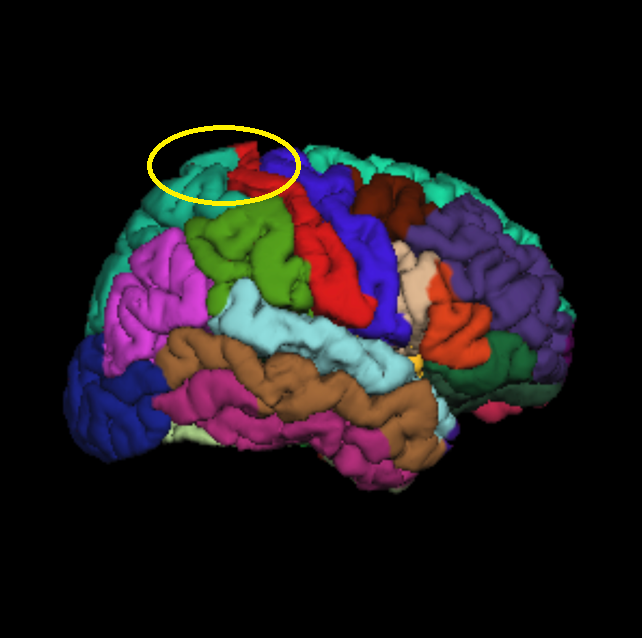


**Figure S3.** The external ENIGMA view of the same subject as in Figure S2. The areas adjacent to the superior sagittal sinus (SSS) protrude outward more than other areas in the image.

## Table

**Table S1**

Effects of superior sagittal sinus (SSS) corrections on cortical thickness (CT) values.

| ROI | SSS not corrected, mean (SD) | SSS corrected, mean (SD) |
| --- | --- | --- |
| Left cuneus | 0.011 (0.029) | -0.013 (0.050) |
| Right cuneus | -0.015 (0.045) | -0.003 (0.042) |
| Left lateral occipital | -0.000 (0.063) | 0.009 (0.077) |
| Right lateral occipital | -0.002 (0.024) | 0.001 (0.024) |
| Left lingual | 0.005 (0.023) | -0.003 (0.042) |
| Right lingual | -0.003 (0.029) | -0.004 (0.039) |
| Left paracentral | 0.020 (0.061) | 0.005 (0.047) |
| Right paracentral | 0.015 (0.053) | 0.003 (0.040) |
| Left pericalcarine | 0.005 (0.038) | 0.000 (0.047) |
| Right pericalcarine | -0.011 (0.035) | 0.005 (0.042) |
| Left postcentral | 0.020 (0.049) | 0.005 (0.033) |
| Right postcentral | 0.030 (0.065) | 0.008 (0.038) |
| Left precentral | 0.014 (0.048) | 0.014 (0.049) |
| Right precentral | 0.024 (0.058) | 0.033 (0.066) |
| Left precuneus | 0.005 (0.028) | -0.000 (0.035) |
| Right precuneus | -0.006 (0.026) | -0.004 (0.034) |
| Left superior frontal | 0.031 (0.062) | 0.017 (0.061) |
| Right superior frontal | 0.039 (0.085) | 0.029 (0.075) |
| Left superior parietal | 0.037 (0.039) | 0.025 (0.055) |
| Right superior parietal | 0.039 (0.060) | 0.036 (0.057) |

Abbreviations: SSS = the superior sagittal sinus, SD = standard deviation. Unit is mm for all values. The values indicate the change in cortical thickness between the unedited and edited images. A positive value indicates that the edited image had a higher CT value. SSS unedited (n = 26), SSS edited (n = 95), however the number of valid datapoints varies by ROI in all methods depending on how many of them passed the ENIGMA quality check. Only areas adjacent to the SSS were included in this analysis. There were no statistically significant differences between groups after Bonferroni correction.

# Subcortex

## Methods

One typical error occurred at the inferior and lateral borders of the putamen, as parts of the white matter (WM) were labeled as putamen. Sometimes the putamen extended past the border between white and gray matter (WM–GM border), and these cases were reported whenever there was any visible putamen on the wrong side of the border (even when the area was vastly smaller than a voxel). An example of this can be seen in Figure S4. This is common problem, since most acquisition protocols lack the resolution to reliably distinguish the structures in this area. These were addressed by adding control points to the mislabeled WM, but the edits were largely unsuccessful. Consequently, we are currently working on separately validating subcortical segmentation procedures for our data.

## Results

Errors with putamen were common in the aseg volume. We attempted to fix these using control points on the brainmask volume, however the edits were largely unsuccessful. After the edits of all 121 images, putamen extended into the left insula in 92 (76.0%) cases, right insula in 55 (45.5%) cases, left lateral orbitofrontal in 33 (27.3%) cases, right lateral orbitofrontal in 35 (28.9%) cases, left medial orbitofrontal in eight (6.6%) cases, and right medial orbitofrontal in one (0.8%) case.

## Figure


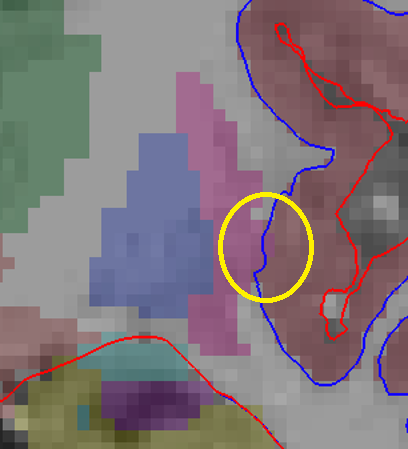


**Figure S4.** Putamen label extends past the blue border between white and gray matter into the insula (yellow circle).

# Optional flags

## Methods

### Image processing

We were interested if the skull stripping as well as minor imperfections on the pial surface could be improved by changing the initial registrations. For this analysis we chose a representative sample of ten participants. We compared the default recon-all to recon-all with certain optional flags: mprage, Schwartz atlas, or both. The magnetization prepared rapid gradient echo = MPRAGE (“-mprage”) optional flag assumes scan parameters for Massachusetts General Hospital (MGH) MPRAGE protocol. The Schwartz atlas (“-schwartzya3t-atlas”) optional flag uses special young adult 3T atlas for Talairach registration. We looked through all coronal slices for instances that might require manual correction, such as pial border extending to the superior sagittal sinus or WM border extending abnormally close to the pial border. Such errors in the automatic segmentation were labelled into three types: pial border, the border between white and gray matter (WM–GM border), and parts of the skull. One researcher (EPP) looked through the slices searching for one type of mistake in one hemisphere at a time. The same type of error in the same hemisphere of the same participant were searched for consecutively in all four different recon-all methods to maximize the internal consistency of the assessment.

### Statistics

For the comparison of different optional flags in FreeSurfer recon-all, the number of slices with errors was considered a continuous variable that was not normally distributed. Therefore, we used Kruskal-Wallis non-parametric ANOVA.

## Results

Results by recon-all option and type of error can be seen in Table S2. There were no statistically significant differences between groups (p > 0.83 in all types of error). Subsequently, we used the standard recon-all for all images in this study as the other options did not provide additional benefits.

## Table

**Table S2**

Recon-all optional flags comparison.

| Reckon-all | Pial border,  Right | Pial border,  Left | WM–GM,  Right | WM–GM,  Left | Skull,  Right | Skull,  Left |
| --- | --- | --- | --- | --- | --- | --- |
| Default | 68 (23) | 67 (19) | 66 (26) | 55 (23) | 8 (12) | 17 (19) |
| MPRAGE | 68 (24) | 69 (20) | 68 (26) | 56 (23) | 8 (12) | 17 (19) |
| Schwartz | 67 (21) | 69 (21) | 68 (27) | 58 (26) | 6 (13) | 15 (19) |
| Schwartz and MPRAGE | 68 (21) | 70 (21) | 69 (28) | 59 (27) | 6 (13) | 15 (19) |

Abbreviations: WM–GM = the border between white matter (WM) and gray matter (GM). Presented as mean (standard deviation). Unit: coronal slices with issues that should be considered for manual corrections. All potential errors, no matter how small, were counted for this part of our study. There were no statistically significant differences between groups in a Kruskal-Wallis non-parametric ANOVA (p > 0.83 in all types of error).

# CAT12

## Methods

### CAT12 processing

All participants were processed with the Computational Anatomy Toolbox (CAT; <http://www.neuro.uni-jena.de/cat/>, version r1363) within Statistical Parametric Mapping (SPM12; <http://www.fil.ion.ucl.ac.uk/spm/software/spm12/>, version 7219) using MATLAB (9.1) to gather cortical thickness (CT) estimates. The CAT12 program has two major processing steps: voxel-based and surface-based. After the initial and refined voxel-based steps, the surface and thickness were estimated utilizing the projection-based thickness method (Dahnke et al., 2013). The following steps in the surface-based processing include topology correction (Yotter et al., 2011), spherical mapping and registration (Yotter et al., 2011). Before segmentation we set the origin to the midline of the anterior commissure. We ran the standard preprocessing pipeline with the following settings: job was not split into separate processes, the default tissue probability map provided in SPM was used, affine regularization was done with the European brains template, inhomogeneity correction was medium, affine preprocessing rough, local adaptive segmentation medium, skull-stripping was done via the Graph cuts (GCUT) approach, voxel size for normalized images was 1.5 and internal resampling for preprocessing fixed 1.0 mm. Dartel was used for spatial registration and the MNI152 template (MNI = Montreal Neurosciences Institute) was used for both Dartel and Shooting templates. Surface and thickness estimation were selected in the writing options.

### Statistics

Used software and other general information are given in the full article.

To compare edited FreeSurfer to CAT12, we conducted a paired samples t-test.

We calculated a linear regression model to assess the agreement between region of interest (ROI) estimates from edited FreeSurfer with unedited FreeSurfer and CAT12. All ROIs of all subjects from both hemispheres separately were included in the analysis, leading to 7 208 datapoints. Coefficient of determination, slope, and intercept were calculated.

## Results

The difference in CT was not significantly different after Bonferroni correction (p < 0.0007) in 15/68 (22.1%) regions. FreeSurfer produced significantly higher values in 14/68 (20.6%) regions, and CAT12 produced significantly higher values in 39/68 (57.4%) regions. All results by ROI can be seen in Table S3.

A linear regression model with FreeSurfer output as independent variables and CAT12 output as dependent variables resulted in R^2^ = 0.525 (Figure S5). The Pearson correlation between the CAT12 and FreeSurfer measures was 0.725 (p < 0.001). Slope was 0.901 (p < 0.001) and intercept 0.357 (p < 0.001). We found a few big outliers in CT measurements within subjects, the extreme cases being the left entorhinal (CAT12, 4.70mm; FreeSurfer, 2.43mm; difference 2.27mm) and the left frontal pole (CAT12, 2.50mm; FreeSurfer, 4.22mm; difference 1.73mm). Pearson correlations were also calculated by ROI, and they ranged from 0.169 in the right insula to 0.711 in the right inferior parietal region. All correlations were positive. Most (61/68 (89.7%)) remained statistically significant after Bonferroni correction. All correlations can be seen in Table S4.

## Discussion

FreeSurfer and CAT12 had relatively low agreement in our sample (R^2^ = 0.525). A previous study in elderly Alzheimer patients and healthy subjects has shown a good agreement in CT values between these software (R^2^ = 0.83) (Seiger et al., 2018). The same study found that CAT12 produced systemically higher values than FreeSurfer. A similar trend was observed in our sample, since CAT12 often produced higher values. However, the opposite was also true in multiple areas, whereas in the previous study, there were no regions where FreeSurfer produced significantly higher values (in healthy subjects). One possible explanation for this difference is the age of the sample. The use of adult template for pediatric population may affect the results in different ways in FreeSurfer and CAT12, as the two have very different approaches in calculating CT: FreeSurfer reconstructs both surfaces and calculates the distance between them, whereas CAT12 takes a volume-based approach using projection-based thickness (PBT) (Dahnke et al., 2013). However, in another study FreeSurfer and CAT12 were compared in a sample of multiple sclerosis (MS) patients and healthy controls (Righart et al., 2017) and the opposite effect was seen: CAT12 produced lower values than FreeSurfer. Seiger et al. propose the use of older versions for both FreeSurfer and CAT12 as a possible explanation. They also performed a test-retest reliability assessment with young adults, showing consistently higher CT values in CAT12 than FreeSurfer, indicating age-independent difference (Seiger et al., 2018). Despite being age-independent in adult samples, the differences with our results may be due to differences in pediatric and adult brains. Furthermore, we used 3.0 Tesla images, like Righart et al., whereas Seiger at al. used 1.5 Tesla images. The reason for these conflicting findings is not clear, and further research is needed.

We have chosen to use FreeSurfer over CAT12 based on the following advantages for our purposes: 1) It is more commonly used in pediatric neuroimaging (Phan et al., 2018) and therefore comparison with other studies is more straightforward; 2) FreeSurfer provides a method to accurately assess image quality and to fix certain types of errors (which were common in our sample) via Freeview; and 3) There are rigorous quality control protocols such as the ENIGMA protocol to make final quality assessment on such a level that allows the researchers to exclude single ROIs with imperfect segmentation.

## Figure


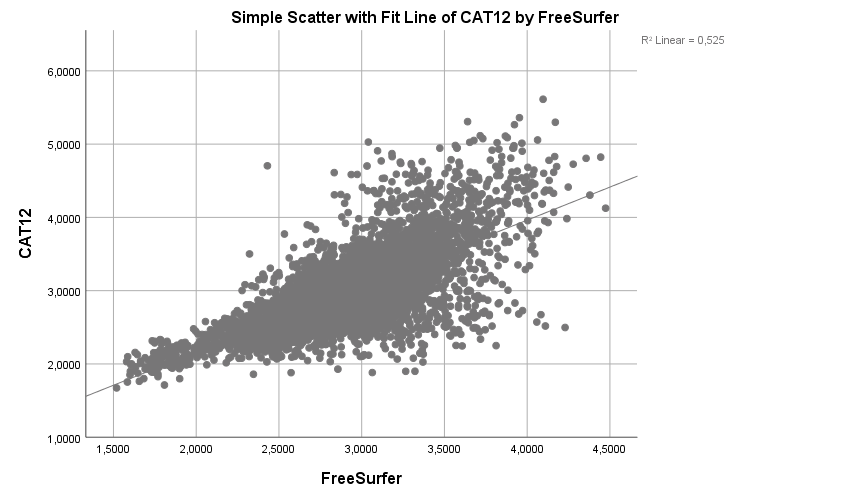


**Figure S5.** A linear regression model with Cortical Thickness (CT, in millimeters) from unedited FreeSurfer as the independent variable and CT from Computational Anatomy Toolbox (CAT12) as the dependent variable. Each datapoint represents a CT measurement from a certain subject, a certain region of interest (ROI), and a certain hemisphere. Total number of datapoints = 7 208 (per method).

## Tables

**Table S3**

Differences in CT measurements between FreeSurfer and CAT12.

| ROI | N | P-value | Mean Difference (mm) |
| --- | --- | --- | --- |
| Left banks of the superior temporal sulcus | 95 | 1.4E-12* | 0.1560 |
| Right banks of the superior temporal sulcus | 110 | 1.8E-15* | 0.1883 |
| Left caudal anterior cingulate | 96 | 6.3E-12* | 0.2347 |
| Right caudal anterior cingulate | 106 | 0.9780 | -0.0009 |
| Left caudal middle frontal | 116 | 2.0E-09* | -0.0990 |
| Right caudal middle frontal | 114 | 5.5E-21* | -0.1886 |
| Left cuneus | 93 | 1.0E-28* | -0.2169 |
| Right cuneus | 92 | 1.6E-15* | -0.1312 |
| Left entorhinal | 121 | 8.0E-41* | -0.8121 |
| Right entorhinal | 120 | 2.9E-34* | -0.7620 |
| Left fusiform | 120 | 0.0085 | -0.0296 |
| Right fusiform | 121 | 2.2E-09* | -0.0789 |
| Left inferior parietal | 102 | 0.2097 | 0.0165 |
| Right inferior parietal | 102 | 0.0361 | -0.0242 |
| Left inferior temporal | 105 | 0.0906 | 0.0236 |
| Right inferior temporal | 94 | 0.0140 | 0.0389 |
| Left isthmus cingulate | 120 | 1.7E-32* | -0.3357 |
| Right isthmus cingulate | 121 | 1.7E-20* | -0.2114 |
| Left lateral occipital | 105 | 9.8E-13* | 0.0938 |
| Right lateral occipital | 111 | 2.8E-20* | 0.1315 |
| Left lateral orbitofrontal | 113 | 2.0E-24* | -0.2361 |
| Right lateral orbitofrontal | 119 | 8.3E-44* | -0.3452 |
| Left lingual | 100 | 0.0023 | -0.0469 |
| Right lingual | 93 | 0.0045 | -0.0418 |
| Left medial orbitofrontal | 95 | 0.0116 | -0.0480 |
| Right medial orbitofrontal | 92 | 0.0038 | 0.0594 |
| Left middle temporal | 77 | 2.5E-07* | -0.1147 |
| Right middle temporal | 74 | 1.1E-10* | -0.1371 |
| Left parahippocampal | 120 | 1.4E-22* | 0.3242 |
| Right parahippocampal | 121 | 0.0002* | 0.0894 |
| Left paracentral | 116 | 7.0E-33* | -0.2016 |
| Right paracentral | 113 | 1.2E-05* | -0.0638 |
| Left pars opercularis | 120 | 4.9E-06* | -0.0967 |
| Right pars opercularis | 117 | 1.0E-06* | -0.1162 |
| Left pars orbitalis | 120 | 0.0001* | -0.1185 |
| Right pars orbitalis | 120 | 4.4E-14* | -0.2219 |
| Left pars triangularis | 120 | 2.3E-06* | -0.0969 |
| Right pars triangularis | 119 | 1.7E-08* | -0.1189 |
| Left pericalcarine | 90 | 8.8E-32* | -0.2513 |
| Right pericalcarine | 87 | 8.2E-31* | -0.2571 |
| Left postcentral | 95 | 1.1E-10* | -0.1129 |
| Right postcentral | 91 | 1.7E-22* | -0.1804 |
| Left posterior cingulate | 119 | 0.0258 | -0.0341 |
| Right posterior cingulate | 119 | 0.0001* | -0.0551 |
| Left precentral | 105 | 0.0002* | -0.0503 |
| Right precentral | 99 | 0.0006* | -0.0495 |
| Left precuneus | 110 | 1.1E-31* | -0.1613 |
| Right precuneus | 118 | 8.4E-27* | -0.1499 |
| Left rostral anterior cingulate | 106 | 8.6E-10* | 0.1801 |
| Right rostral anterior cingulate | 119 | 1.2E-09* | 0.1661 |
| Left rostral middle frontal | 119 | 2.0E-06* | -0.0713 |
| Right rostral middle frontal | 116 | 3.2E-08* | -0.0819 |
| Left superior frontal | 81 | 2.4E-08* | -0.1262 |
| Right superior frontal | 97 | 5.9E-08* | -0.1077 |
| Left superior parietal | 93 | 0.0964 | -0.0201 |
| Right superior parietal | 88 | 2.4E-06* | -0.0728 |
| Left superior temporal | 77 | 1.1E-07* | 0.1106 |
| Right superior temporal | 83 | 0.0017 | 0.0527 |
| Left supramarginal | 86 | 4.6E-07* | 0.0937 |
| Right supramarginal | 88 | 0.0001* | 0.0743 |
| Left frontal pole | 119 | 4.5E-31* | 0.6513 |
| Right frontal pole | 120 | 1.1E-24* | 0.5472 |
| Left temporal pole | 117 | 9.5E-16* | -0.3801 |
| Right temporal pole | 118 | 1.8E-23* | -0.4485 |
| Left transverse temporal | 121 | 0.3811 | 0.0313 |
| Right transverse temporal | 121 | 0.0031 | 0.1083 |
| Left insula | 100 | 1.5E-08* | -0.1506 |
| Right insula | 93 | 2.0E-06* | -0.1332 |

Abbreviations: CT = cortical thickness, CAT12 = Computational Anatomy Toolbox, ROI = region of interest, N = number of participants, * = statistically significant after Bonferroni correction (p < 0.0007). Results of the paired sample t test comparing CAT12 and edited FreeSurfer. In mean differences, positive value indicates that FreeSurfer produced higher values than CAT12. Total number of participants was 121, the number of approved subjects varied by ROI according to how many passed the ENIGMA quality control protocol.

**Table S4**

Pearson’s correlation between FreeSurfer and CAT12 CT measurements.

| ROI | N | P-value | Correlation |
| --- | --- | --- | --- |
| Left banks of the superior temporal sulcus | 95 | 1.1E-13* | 0.670 |
| Right banks of the superior temporal sulcus | 110 | 4.1E-13* | 0.622 |
| Left caudal anterior cingulate | 96 | 8.6E-12* | 0.627 |
| Right caudal anterior cingulate | 106 | 3.5E-06* | 0.433 |
| Left caudal middle frontal | 116 | 7.8E-07* | 0.440 |
| Right caudal middle frontal | 114 | 7.2E-08* | 0.479 |
| Left cuneus | 93 | 6.9E-15* | 0.699 |
| Right cuneus | 92 | 2.6E-15* | 0.709 |
| Left entorhinal | 121 | 0.0004* | 0.318 |
| Right entorhinal | 120 | 4.8E-06* | 0.404 |
| Left fusiform | 120 | 1.3E-15* | 0.648 |
| Right fusiform | 121 | 2.4E-13* | 0.604 |
| Left inferior parietal | 102 | 3.5E-11* | 0.597 |
| Right inferior parietal | 102 | 5.4E-17* | 0.711 |
| Left inferior temporal | 105 | 4.3E-13* | 0.633 |
| Right inferior temporal | 94 | 1.8E-12* | 0.648 |
| Left isthmus cingulate | 120 | 8.2E-09* | 0.496 |
| Right isthmus cingulate | 121 | 4.5E-10* | 0.529 |
| Left lateral occipital | 105 | 1.1E-16* | 0.699 |
| Right lateral occipital | 111 | 3.3E-16* | 0.677 |
| Left lateral orbitofrontal | 113 | 9.6E-08* | 0.476 |
| Right lateral orbitofrontal | 119 | 2.1E-10* | 0.541 |
| Left lingual | 100 | 1.3E-08* | 0.531 |
| Right lingual | 93 | 1.3E-09* | 0.578 |
| Left medial orbitofrontal | 95 | 1.4E-09* | 0.572 |
| Right medial orbitofrontal | 92 | 8.9E-06* | 0.445 |
| Left middle temporal | 77 | 1.2E-08* | 0.595 |
| Right middle temporal | 74 | 1.8E-09* | 0.630 |
| Left parahippocampal | 120 | 1.3E-05* | 0.387 |
| Right parahippocampal | 121 | 1.1E-08* | 0.490 |
| Left paracentral | 116 | 2.0E-14* | 0.635 |
| Right paracentral | 113 | 2.5E-08* | 0.495 |
| Left pars opercularis | 120 | 0.0003* | 0.324 |
| Right pars opercularis | 117 | 0.0010 | 0.299 |
| Left pars orbitalis | 120 | 3.4E-08* | 0.478 |
| Right pars orbitalis | 120 | 3.3E-11* | 0.559 |
| Left pars triangularis | 120 | 9.7E-06* | 0.392 |
| Right pars triangularis | 119 | 1.8E-06* | 0.421 |
| Left pericalcarine | 90 | 3.8E-13* | 0.673 |
| Right pericalcarine | 87 | 8.2E-10* | 0.600 |
| Left postcentral | 95 | 1.1E-05* | 0.435 |
| Right postcentral | 91 | 4.4E-11* | 0.623 |
| Left posterior cingulate | 119 | 5.0E-12* | 0.579 |
| Right posterior cingulate | 119 | 4.4E-15* | 0.640 |
| Left precentral | 105 | 6.2E-08* | 0.499 |
| Right precentral | 99 | 1.7E-06* | 0.460 |
| Left precuneus | 110 | 6.2E-17* | 0.691 |
| Right precuneus | 118 | 3.3E-10* | 0.538 |
| Left rostral anterior cingulate | 106 | 2.4E-08* | 0.509 |
| Right rostral anterior cingulate | 119 | 8.2E-11* | 0.551 |
| Left rostral middle frontal | 119 | 7.3E-07* | 0.436 |
| Right rostral middle frontal | 116 | 3.4E-06* | 0.416 |
| Left superior frontal | 81 | 0.0018 | 0.342 |
| Right superior frontal | 97 | 0.0006* | 0.341 |
| Left superior parietal | 93 | 2.5E-11* | 0.623 |
| Right superior parietal | 88 | 0.0001* | 0.403 |
| Left superior temporal | 77 | 8.1E-08* | 0.566 |
| Right superior temporal | 83 | 3.2E-12* | 0.673 |
| Left supramarginal | 86 | 6.3E-06* | 0.465 |
| Right supramarginal | 88 | 0.0001* | 0.397 |
| Left frontal pole | 119 | 0.0076 | 0.244 |
| Right frontal pole | 120 | 0.0352 | 0.192 |
| Left temporal pole | 117 | 0.0005* | 0.315 |
| Right temporal pole | 118 | 4.1E-12* | 0.584 |
| Left transverse temporal | 121 | 0.0057 | 0.250 |
| Right transverse temporal | 121 | 0.0026 | 0.271 |
| Left insula | 100 | 1.1E-05* | 0.423 |
| Right insula | 93 | 0.1060 | 0.169 |

Abbreviations: CAT12 = Computational Anatomy Toolbox, CT = cortical thickness, ROI = region of interest, N = number of participants, * = statistically significant after Bonferroni correction (p < 0.0007). Edited FreeSurfer images were used in this comparison. Total number of participants was 121, the number of approved subjects varied by ROI according to how many passed the ENIGMA quality control protocol.

# Qoala-T

## Methods

Qoala-T is a supervised learning tool for quality control (QC) of automated labeling processed in FreeSurfer, and it is particularly intended to use in developmental datasets (Klapwijk et al., 2019). We applied Qoala-T (version 1.2.1) using R 4.1.3 (R Core Team (2021). R: A language and environment for statistical computing. R Foundation for Statistical Computing, Vienna, Austria. URL <https://www.R-project.org/>) in RStudio (version 2021.09.1.+372.pro1).

### Statistics

We compared Qoala-T scores from all 134 participants that entered the FreeSurfer segmentation protocol.

We described frequencies of quality control assessments in four categories according to the Qoala-T recommendations (and in which category the participants we excluded were placed in): 1) exclude, no manual QC; 2) exclude, manual QC; 3) include, manual QC; and 4) include, no manual QC. These were reported for both unedited and edited images separately. Furthermore, we performed a paired samples t-test to compare the change in Qoala-T scores between unedited and edited images.

## Results

In the unedited images, the recommendations were: exclude, no manual QC (number of participants (n) = 24, 17.9%), exclude, manual QC (n = 27, 20.1%), include, manual QC (n = 70, 52.2%), include, no manual QC (n = 13, 9.7%). Overall, manual QC was recommended for 97 (72.4%) participants.

In the edited images, the recommendations were: exclude, no manual QC (n = 19, 14.2%), exclude, manual QC (n = 25, 18.7%), include, manual QC (n = 74, 55.2%), include, no manual QC (n = 16, 11.9%). Overall, manual QC was recommended for 99 (73.9%) participants.

In both unedited and edited images, 12/13 images that we excluded were in the “exclude, no manual QC” category, and one was in the “include, manual QC” category.

Figure S6 presents the distribution of Qoala-T scores in unedited images and Figure S7 presents the distribution of Qoala-T scores in edited images. In both images, the participants excluded in the FinnBrain QC protocol are represented by black circles.

The mean Qoala-T score in unedited images was 50.95 (standard deviation (SD) = 17.62). The mean Qoala-T score in edited images was 52.70 (SD = 17.20). This difference was statistically significant (p = 0.000078).

## Discussion

Our results show that manual edits increased Qoala-T scores and therefore the number of participants that passed the Qoala-T QC protocol. Apart from one exception, the quality ratings matched our exclusions well. The one exception had a lot of missing gray matter (GM) in superior parietal regions, and was therefore excluded according to our protocol. Notably, the Qoala-T protocol also recommended manual QC for that participant, allowing for that error to be found in that protocol, too. The last notable finding from this analysis was the high number of participants, for whom a manual QC was recommended. In other words, even with the help of an automated tool, there may still be need for manual QC with the majority of the participants.

## Figures


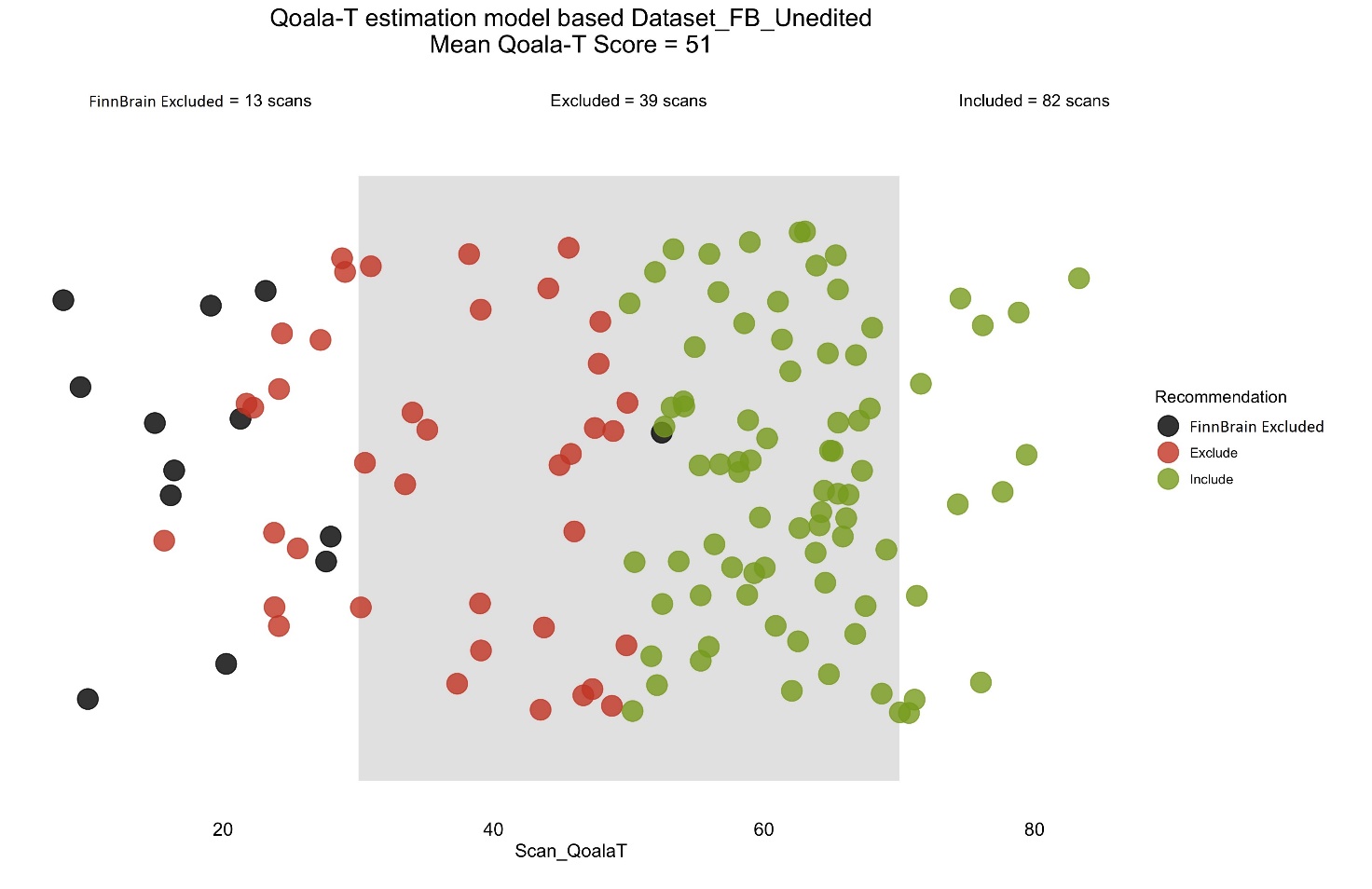


**Figure S6.** Qoala-T scores of FreeSurfer images before manual editing. Black circles = the participants excluded in the FinnBrain quality control (QC) protocol, red circles = the participants Qoala-T recommends for exclusion, green circles = the participants Qoala-T recommends for inclusion, gray rectangle = the circles in this area are recommended for manual QC. Participants are randomly placed on the Y-axis.


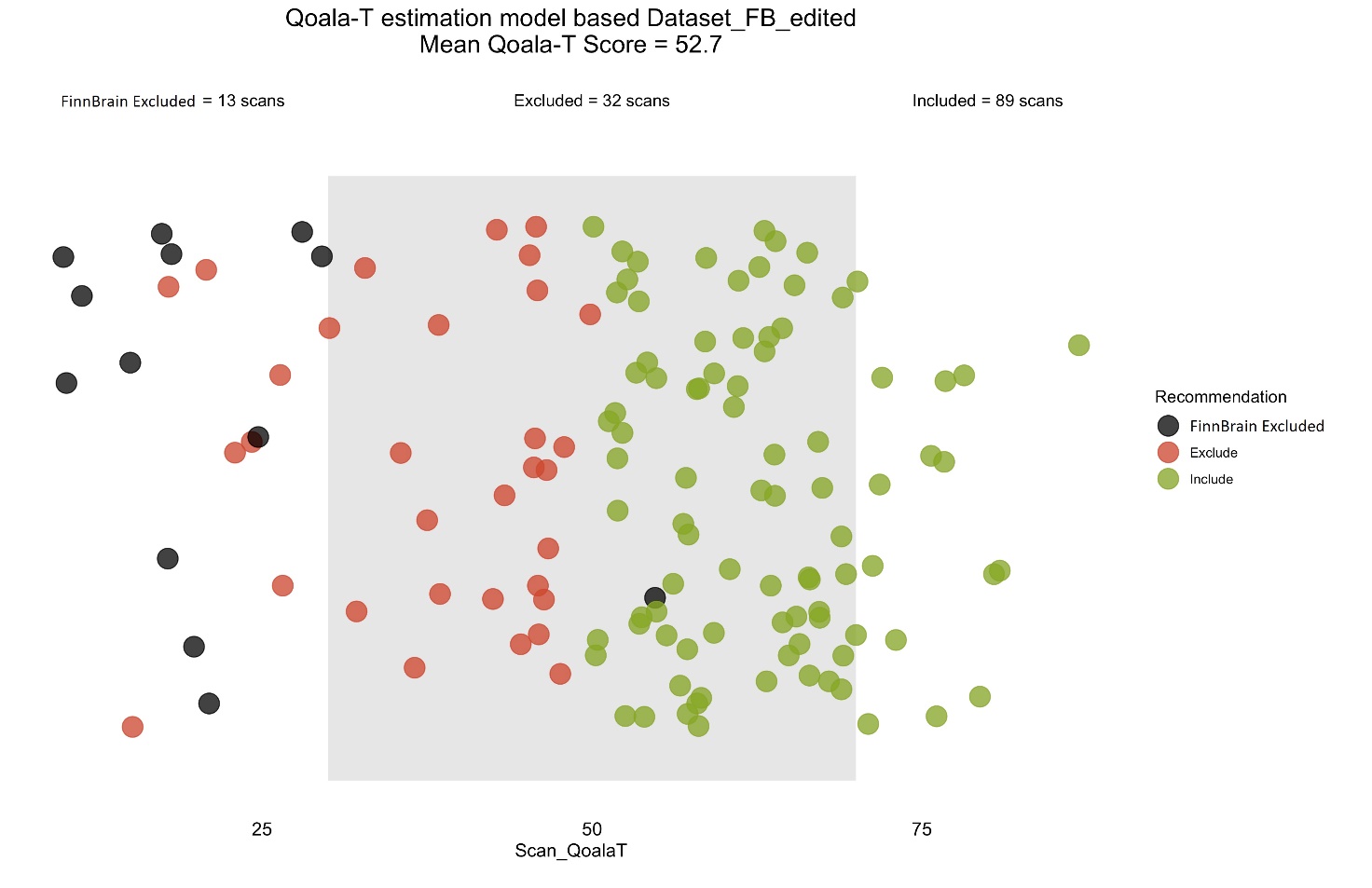


**Figure S7.** Qoala-T scores of FreeSurfer images after manual editing. Black circles = the participants excluded in the FinnBrain quality control (QC) protocol, red circles = the participants Qoala-T recommends for exclusion, green circles = the participants Qoala-T recommends for inclusion, gray rectangle = the circles in this area are recommended for manual QC. Participants are randomly placed on the Y-axis.

# References

Dahnke, R., Yotter, R. A., & Gaser, C. (2013). Cortical thickness and central surface estimation. *NeuroImage*, *65*, 336–348. https://doi.org/10.1016/j.neuroimage.2012.09.050

Klapwijk, E. T., van de Kamp, F., van der Meulen, M., Peters, S., & Wierenga, L. M. (2019). Qoala-T: A supervised-learning tool for quality control of FreeSurfer segmented MRI data. *NeuroImage*, *189*, 116–129. https://doi.org/10.1016/j.neuroimage.2019.01.014

Phan, T. V., Smeets, D., Talcott, J. B., & Vandermosten, M. (2018). Processing of structural neuroimaging data in young children: Bridging the gap between current practice and state-of-the-art methods. In *Developmental Cognitive Neuroscience* (Vol. 33, pp. 206–223). Elsevier Ltd. https://doi.org/10.1016/j.dcn.2017.08.009

Righart, R., Schmidt, P., Dahnke, R., Biberacher, V., Beer, A., Buck, D., Hemmer, B., Kirschke, J. S., Zimmer, C., Gaser, C., & Mühlau, M. (2017). Volume versus surface-based cortical thickness measurements: A comparative study with healthy controls and multiple sclerosis patients. *PLOS ONE*, *12*(7), e0179590. https://doi.org/10.1371/journal.pone.0179590

Seiger, R., Ganger, S., Kranz, G. S., Hahn, A., & Lanzenberger, R. (2018). Cortical Thickness Estimations of FreeSurfer and the CAT12 Toolbox in Patients with Alzheimer’s Disease and Healthy Controls. *Journal of Neuroimaging*, *28*(5), 515–523. https://doi.org/10.1111/jon.12521

Yotter, Rachel A., Thompson, P. M., & Gaser, C. (2011). Algorithms to Improve the Reparameterization of Spherical Mappings of Brain Surface Meshes. *Journal of Neuroimaging*, *21*(2), e134–e147. https://doi.org/10.1111/j.1552-6569.2010.00484.x

Yotter, Rachel Aine, Dahnke, R., Thompson, P. M., & Gaser, C. (2011). Topological correction of brain surface meshes using spherical harmonics. *Human Brain Mapping*, *32*(7), 1109–1124. https://doi.org/10.1002/hbm.21095
