# Supplementary material for: Feasibility of FreeSurfer Processing for T1-Weighted Brain Images of 5-Year-Olds: Semiautomated Protocol of FinnBrain Neuroimaging Lab
Source: Front Neurosci. 2022 May 2;16:874062. doi: 10.3389/fnins.2022.874062 (PMC9108497; doi:10.3389/fnins.2022.874062)
Supplement: Supplementary file 3 [file Data_Sheet_3.PDF]

Results

Sex Differences - Cortical Thickness - Quality Controlled

Independent Samples T-Test

	t	df	p	Mean Difference	SE Difference	95% CI for Mean Difference		Cohen's d
						Lower	Upper	
lh_bankssts_CT_QC	−1.289	93	0.200	−0.054	0.042	−0.138	0.029	−0.266
lh_caudalanteriorcingulate_CT_QC	−1.610	94	0.111	−0.095	0.059	−0.213	0.022	−0.330
lh_caudalmiddlefrontal_CT_QC	−0.972	114	0.333	−0.027	0.028	−0.081	0.028	−0.181
lh_cuneus_CT_QC	−1.867	91	0.065	−0.058	0.031	−0.120	0.004	−0.388
lh_entorhinal_CT_QC	−1.085	119	0.280	−0.063	0.058	−0.179	0.052	−0.198
lh_fusiform_CT_QC	−2.134	118	0.035	−0.044	0.020	−0.084	−0.003	−0.391
lh_inferiorparietal_CT_QC	−2.225	100	0.028	−0.051	0.023	−0.097	−0.006	−0.441
lh_inferiortemporal_CT_QC	−1.050	103	0.296	−0.029	0.027	−0.083	0.025	−0.205
lh_isthmuscingulate_CT_QC	−2.708	118	0.008	−0.091	0.033	−0.157	−0.024	−0.495
lh_lateraloccipital_CT_QC	−1.526	103	0.130	−0.034	0.023	−0.079	0.010	−0.298
lh_lateralorbitofrontal_CT_QC	0.752	111	0.454	0.023	0.030	−0.037	0.082	0.142
lh_lingual_CT_QC	−1.612	98	0.110	−0.045	0.028	−0.101	0.010	−0.324
lh_medialorbitofrontal_CT_QC	−0.221	93	0.825	−0.008	0.037	−0.081	0.065	−0.046
lh_middletemporal_CT_QC	−0.577	75	0.565	−0.017	0.030	−0.076	0.042	−0.132
lh_parahippocampal_CT_QC	−3.267	118	0.001	−0.164	0.050	−0.263	−0.065	−0.597
lh_paracentral_CT_QC	−1.892	114	0.061	−0.049	0.026	−0.100	0.002	−0.352
lh_parsopercularis_CT_QC	−1.071	118	0.287	−0.025	0.024	−0.072	0.021	−0.196
lh_parsorbitalis_CT_QC	−1.925	118	0.057	−0.075	0.039	−0.153	0.002	−0.352
lh_parstriangularis_CT_QC	−1.642	118	0.103	−0.048	0.029	−0.105	0.010	−0.300
lh_pericalcarine_CT_QC	−1.268	88	0.208	−0.042	0.033	−0.107	0.024	−0.268
lh_postcentral_CT_QC	−1.201	93	0.233	−0.032	0.027	−0.086	0.021	−0.247
lh_posteriorcingulate_CT_QC	−1.261	117	0.210	−0.035	0.028	−0.091	0.020	−0.232
lh_precentral_CT_QC	−0.586	103	0.559	−0.013	0.022	−0.057	0.031	−0.115
lh_precuneus_CT_QC	−1.573	108	0.119	−0.036	0.023	−0.081	0.009	−0.300
lh_rostralanteriorcingulate_CT_QC	−1.930	104	0.056	−0.089	0.046	−0.180	0.002	−0.376
lh_rostralmiddlefrontal_CT_QC	−0.885	117	0.378	−0.022	0.025	−0.072	0.028	−0.162
lh_superiorfrontal_CT_QC	−1.797	79	0.076	−0.056	0.031	−0.119	0.006	−0.400
lh_superiorparietal_CT_QC	−3.605	91	< .001	−0.088	0.024	−0.137	−0.040	−0.750
lh_superiortemporal_CT_QC	−1.893	75	0.062	−0.059	0.031	−0.121	0.003	−0.433
lh_supramarginal_CT_QC	−1.590	84	0.116	−0.047	0.029	−0.105	0.012	−0.343
lh_frontalpole_CT_QC	−2.282	117	0.024	−0.137	0.060	−0.256	−0.018	−0.419
lh_temporalpole_CT_QC	−0.228	115	0.820	−0.013	0.056	−0.124	0.098	−0.042
lh_transversetemporal_CT_QC	−2.704	119	0.008	−0.125	0.046	−0.216	−0.033	−0.492
lh_insula_CT_QC	−0.500	98	0.618	−0.016	0.031	−0.077	0.046	−0.100
rh_bankssts_CT_QC	−1.903	108	0.060	−0.069	0.036	−0.141	0.003	−0.363
rh_caudalanteriorcingulate_CT_QC	−0.427	104	0.670	−0.021	0.049	−0.117	0.076	−0.083
rh_caudalmiddlefrontal_CT_QC	0.011	112	0.991	3.333e−4	0.031	−0.061	0.061	0.002
rh_cuneus_CT_QC	−1.687	90	0.095	−0.054	0.032	−0.117	0.010	−0.352
rh_entorhinal_CT_QC	−0.540	118	0.590[a]	−0.035	0.065	−0.164	0.094	−0.099
rh_fusiform_CT_QC	−1.896	119	0.060	−0.037	0.020	−0.076	0.002	−0.345
rh_inferiorparietal_CT_QC	−2.094	100	0.039	−0.057	0.027	−0.112	−0.003	−0.415
rh_inferiortemporal_CT_QC	−1.042	92	0.300	−0.031	0.030	−0.090	0.028	−0.215
rh_isthmuscingulate_CT_QC	−1.961	119	0.052	−0.064	0.033	−0.129	6.236e−4	−0.357
rh_lateraloccipital_CT_QC	−2.538	109	0.013	−0.058	0.023	−0.104	−0.013	−0.482
rh_lateralorbitofrontal_CT_QC	−0.834	117	0.406[a]	−0.025	0.030	−0.083	0.034	−0.153
rh_lingual_CT_QC	−1.532	91	0.129	−0.043	0.028	−0.098	0.013	−0.318
rh_medialorbitofrontal_CT_QC	−2.133	90	0.036	−0.075	0.035	−0.144	−0.005	−0.445
rh_middletemporal_CT_QC	−0.617	72	0.539	−0.020	0.032	−0.085	0.045	−0.145
rh_parahippocampal_CT_QC	−2.780	119	0.006	−0.118	0.042	−0.201	−0.034	−0.506
rh_paracentral_CT_QC	−1.205	111	0.231[a]	−0.030	0.025	−0.080	0.019	−0.227
rh_parsopercularis_CT_QC	0.215	115	0.830	0.005	0.024	−0.042	0.053	0.040
rh_parsorbitalis_CT_QC	−1.426	118	0.156	−0.059	0.041	−0.140	0.023	−0.261
rh_parstriangularis_CT_QC	−1.269	117	0.207	−0.036	0.028	−0.092	0.020	−0.233
rh_pericalcarine_CT_QC	−3.092	85	0.003	−0.088	0.029	−0.145	−0.032	−0.665
rh_postcentral_CT_QC	−2.364	89	0.020	−0.065	0.028	−0.120	−0.010	−0.498
rh_posteriorcingulate_CT_QC	−0.857	117	0.393	−0.025	0.029	−0.081	0.032	−0.157
rh_precentral_CT_QC	−0.945	97	0.347	−0.023	0.025	−0.073	0.026	−0.190
rh_precuneus_CT_QC	−0.927	116	0.356	−0.018	0.020	−0.058	0.021	−0.171
rh_rostralanteriorcingulate_CT_QC	−2.791	117	0.006	−0.122	0.044	−0.209	−0.035	−0.513
rh_rostralmiddlefrontal_CT_QC	0.457	114	0.648[a]	0.011	0.023	−0.035	0.056	0.085
rh_superiorfrontal_CT_QC	−1.362	95	0.176	−0.036	0.027	−0.089	0.017	−0.277
rh_superiorparietal_CT_QC	−1.626	86	0.108	−0.039	0.024	−0.087	0.009	−0.348
rh_superiortemporal_CT_QC	−1.108	81	0.271	−0.037	0.033	−0.103	0.029	−0.243
rh_supramarginal_CT_QC	−1.857	86	0.067	−0.049	0.026	−0.101	0.003	−0.396
rh_frontalpole_CT_QC	−2.091	118	0.039	−0.117	0.056	−0.227	−0.006	−0.383
rh_temporalpole_CT_QC	−0.028	116	0.978	−0.002	0.064	−0.129	0.125	−0.005
rh_transversetemporal_CT_QC	−2.358	119	0.020	−0.110	0.047	−0.202	−0.018	−0.429
rh_insula_CT_QC	−1.154	91	0.252	−0.040	0.034	−0.108	0.029	−0.239

Note. Student's t-test.

[a] Levene's test is significant ($p < .05$), suggesting a violation of the equal variance assumption

Descriptives

Group Descriptives

	Group	N	Mean	SD	SE
lh_bankssts_CT_QC	Boy	52	2.969	0.209	0.029
	Girl	43	3.023	0.197	0.030
lh_caudalanteriorcingulate_CT_QC	Boy	52	2.809	0.266	0.037
	Girl	44	2.905	0.316	0.048
lh_caudalmiddlefrontal_CT_QC	Boy	62	2.860	0.138	0.018
	Girl	54	2.887	0.158	0.022
lh_cuneus_CT_QC	Boy	50	2.251	0.165	0.023
	Girl	43	2.309	0.129	0.020
lh_entorhinal_CT_QC	Boy	64	3.216	0.306	0.038
	Girl	57	3.279	0.335	0.044
lh_fusiform_CT_QC	Boy	64	3.048	0.104	0.013
	Girl	56	3.092	0.120	0.016
lh_inferiorparietal_CT_QC	Boy	54	2.940	0.114	0.015
	Girl	48	2.991	0.119	0.017
lh_inferiortemporal_CT_QC	Boy	53	3.184	0.141	0.019
	Girl	52	3.213	0.138	0.019
lh_isthmuscingulate_CT_QC	Boy	64	2.649	0.161	0.020
	Girl	56	2.740	0.206	0.027
lh_lateraloccipital_CT_QC	Boy	54	2.500	0.100	0.014
	Girl	51	2.535	0.130	0.018
lh_lateralorbitofrontal_CT_QC	Boy	62	3.101	0.154	0.020
	Girl	51	3.079	0.166	0.023
lh_lingual_CT_QC	Boy	55	2.387	0.134	0.018
	Girl	45	2.432	0.147	0.022
lh_medialorbitofrontal_CT_QC	Boy	52	2.846	0.163	0.023
	Girl	43	2.854	0.194	0.030
lh_middletemporal_CT_QC	Boy	35	3.271	0.141	0.024
	Girl	42	3.288	0.120	0.019
lh_parahippocampal_CT_QC	Boy	63	2.955	0.274	0.035
	Girl	57	3.119	0.274	0.036
lh_paracentral_CT_QC	Boy	61	2.831	0.144	0.018
	Girl	55	2.880	0.134	0.018
lh_parsopercularis_CT_QC	Boy	63	2.982	0.127	0.016
	Girl	57	3.007	0.131	0.017
lh_parsorbitalis_CT_QC	Boy	63	3.226	0.210	0.026
	Girl	57	3.302	0.219	0.029
lh_parstriangularis_CT_QC	Boy	63	2.980	0.166	0.021
	Girl	57	3.028	0.149	0.020
lh_pericalcarine_CT_QC	Boy	49	1.859	0.157	0.022
	Girl	41	1.901	0.155	0.024
lh_postcentral_CT_QC	Boy	49	2.408	0.114	0.016
	Girl	46	2.440	0.148	0.022
lh_posteriorcingulate_CT_QC	Boy	63	2.810	0.160	0.020
	Girl	56	2.845	0.142	0.019
lh_precentral_CT_QC	Boy	58	2.781	0.101	0.013
	Girl	47	2.794	0.125	0.018
lh_precuneus_CT_QC	Boy	58	2.885	0.110	0.014
	Girl	52	2.920	0.129	0.018
lh_rostralanteriorcingulate_CT_QC	Boy	57	3.085	0.233	0.031
	Girl	49	3.174	0.238	0.034
lh_rostralmiddlefrontal_CT_QC	Boy	63	2.852	0.128	0.016
	Girl	56	2.874	0.147	0.020
lh_superiorfrontal_CT_QC	Boy	43	3.139	0.115	0.018
	Girl	38	3.196	0.165	0.027
lh_superiorparietal_CT_QC	Boy	50	2.597	0.099	0.014
	Girl	43	2.685	0.136	0.021
lh_superiortemporal_CT_QC	Boy	35	3.195	0.132	0.022
	Girl	42	3.254	0.139	0.022
lh_supramarginal_CT_QC	Boy	45	2.999	0.129	0.019
	Girl	41	3.046	0.143	0.022
lh_frontalpole_CT_QC	Boy	62	3.347	0.325	0.041
	Girl	57	3.484	0.331	0.044
lh_temporalpole_CT_QC	Boy	63	3.571	0.294	0.037
	Girl	54	3.584	0.311	0.042
lh_transversetemporal_CT_QC	Boy	64	2.796	0.265	0.033
	Girl	57	2.921	0.240	0.032
lh_insula_CT_QC	Boy	54	3.236	0.160	0.022
	Girl	46	3.252	0.148	0.022
rh_bankssts_CT_QC	Boy	55	3.025	0.201	0.027
	Girl	55	3.094	0.181	0.024
rh_caudalanteriorcingulate_CT_QC	Boy	53	2.746	0.261	0.036
	Girl	53	2.766	0.239	0.033
rh_caudalmiddlefrontal_CT_QC	Boy	57	2.885	0.149	0.020
	Girl	57	2.885	0.179	0.024
rh_cuneus_CT_QC	Boy	48	2.300	0.151	0.022

Group Descriptives

	Group	N	Mean	SD	SE
	Girl	44	2.354	0.156	0.023
rh_entorhinal_CT_QC	Boy	63	3.423	0.399	0.050
	Girl	57	3.458	0.303	0.040
rh_fusiform_CT_QC	Boy	64	3.054	0.108	0.014
	Girl	57	3.091	0.107	0.014
rh_inferiorparietal_CT_QC	Boy	50	2.928	0.146	0.021
	Girl	52	2.986	0.131	0.018
rh_inferiortemporal_CT_QC	Boy	48	3.173	0.139	0.020
	Girl	46	3.203	0.147	0.022
rh_isthmuscingulate_CT_QC	Boy	64	2.660	0.173	0.022
	Girl	57	2.724	0.187	0.025
rh_lateraloccipital_CT_QC	Boy	58	2.567	0.114	0.015
	Girl	53	2.625	0.129	0.018
rh_lateralorbitofrontal_CT_QC	Boy	64	3.020	0.178	0.022
	Girl	55	3.045	0.138	0.019
rh_lingual_CT_QC	Boy	47	2.415	0.133	0.019
	Girl	46	2.458	0.135	0.020
rh_medialorbitofrontal_CT_QC	Boy	48	2.906	0.169	0.024
	Girl	44	2.980	0.166	0.025
rh_middletemporal_CT_QC	Boy	32	3.296	0.117	0.021
	Girl	42	3.316	0.152	0.023
rh_parahippocampal_CT_QC	Boy	64	2.844	0.235	0.029
	Girl	57	2.961	0.229	0.030
rh_paracentral_CT_QC	Boy	60	2.856	0.145	0.019
	Girl	53	2.886	0.118	0.016
rh_parsopercularis_CT_QC	Boy	61	3.005	0.130	0.017
	Girl	56	3.000	0.128	0.017
rh_parsorbitalis_CT_QC	Boy	63	3.232	0.220	0.028
	Girl	57	3.291	0.230	0.030
rh_parstriangularis_CT_QC	Boy	63	3.020	0.145	0.018
	Girl	56	3.056	0.164	0.022
rh_pericalcarine_CT_QC	Boy	47	1.797	0.129	0.019
	Girl	40	1.886	0.137	0.022
rh_postcentral_CT_QC	Boy	50	2.367	0.118	0.017
	Girl	41	2.432	0.146	0.023
rh_posteriorcingulate_CT_QC	Boy	62	2.773	0.172	0.022
	Girl	57	2.798	0.136	0.018
rh_precentral_CT_QC	Boy	53	2.742	0.106	0.015
	Girl	46	2.765	0.141	0.021
rh_precuneus_CT_QC	Boy	63	2.878	0.101	0.013
	Girl	55	2.896	0.114	0.015
rh_rostralanteriorcingulate_CT_QC	Boy	63	3.090	0.247	0.031
	Girl	56	3.212	0.228	0.030
rh_rostralmiddlefrontal_CT_QC	Boy	61	2.885	0.106	0.014
	Girl	55	2.874	0.142	0.019
rh_superiorfrontal_CT_QC	Boy	46	3.144	0.128	0.019
	Girl	51	3.180	0.135	0.019
rh_superiorparietal_CT_QC	Boy	48	2.615	0.108	0.016
	Girl	40	2.654	0.118	0.019
rh_superiortemporal_CT_QC	Boy	40	3.291	0.146	0.023
	Girl	43	3.328	0.156	0.024
rh_supramarginal_CT_QC	Boy	44	3.004	0.128	0.019
	Girl	44	3.053	0.119	0.018
rh_frontalpole_CT_QC	Boy	64	3.270	0.294	0.037
	Girl	56	3.387	0.317	0.042
rh_temporalpole_CT_QC	Boy	63	3.704	0.317	0.040
	Girl	55	3.705	0.379	0.051
rh_transversetemporal_CT_QC	Boy	64	2.846	0.257	0.032
	Girl	57	2.956	0.255	0.034
rh_insula_CT_QC	Boy	48	3.234	0.152	0.022
	Girl	45	3.274	0.180	0.027

Sex Differences - Cortical Thickness - No Quality Control

Independent Samples T-Test

	t	df	p	Mean Difference	SE Difference	95% CI for Mean Difference Lower	Upper	Cohen's d
lh_bankssts_CT_no_QC	−1.557	119	0.122	−0.059	0.038	−0.133	0.016	−0.284
lh_caudalanteriorcingulate_CT_no_QC	−1.984	119	0.050[a]	−0.102	0.051	−0.203	−1.772e−4	−0.361
lh_caudalmiddlefrontal_CT_no_QC	−1.659	119	0.100	−0.051	0.031	−0.113	0.010	−0.302
lh_cuneus_CT_no_QC	−2.372	119	0.019	−0.067	0.028	−0.123	−0.011	−0.432
lh_entorhinal_CT_no_QC	−0.871	119	0.386	−0.049	0.057	−0.162	0.063	−0.159
lh_fusiform_CT_no_QC	−2.014	119	0.046	−0.039	0.019	−0.077	−6.500e−4	−0.367
lh_inferiorparietal_CT_no_QC	−2.216	119	0.029	−0.054	0.024	−0.103	−0.006	−0.404
lh_inferiortemporal_CT_no_QC	−2.037	119	0.044	−0.055	0.027	−0.109	−0.002	−0.371
lh_isthmuscingulate_CT_no_QC	−2.800	119	0.006	−0.092	0.033	−0.157	−0.027	−0.510
lh_lateraloccipital_CT_no_QC	−1.954	119	0.053	−0.043	0.022	−0.086	5.721e−4	−0.356
lh_lateralorbitofrontal_CT_no_QC	0.063	119	0.950	0.002	0.028	−0.053	0.057	0.011
lh_lingual_CT_no_QC	−2.200	119	0.030[a]	−0.058	0.026	−0.110	−0.006	−0.401
lh_medialorbitofrontal_CT_no_QC	−1.321	119	0.189	−0.047	0.035	−0.117	0.023	−0.241
lh_middletemporal_CT_no_QC	−1.874	119	0.063	−0.054	0.029	−0.111	0.003	−0.341
lh_parahippocampal_CT_no_QC	−3.042	119	0.003	−0.152	0.050	−0.251	−0.053	−0.554
lh_paracentral_CT_no_QC	−1.872	119	0.064	−0.051	0.027	−0.104	0.003	−0.341
lh_parsopercularis_CT_no_QC	−0.887	119	0.377	−0.020	0.022	−0.064	0.024	−0.161
lh_parsorbitalis_CT_no_QC	−1.312	119	0.192	−0.057	0.043	−0.142	0.029	−0.239
lh_parstriangularis_CT_no_QC	−1.216	119	0.226	−0.037	0.030	−0.097	0.023	−0.221
lh_pericalcarine_CT_no_QC	−1.657	119	0.100	−0.046	0.027	−0.100	0.009	−0.302
lh_postcentral_CT_no_QC	−2.243	119	0.027	−0.050	0.022	−0.095	−0.006	−0.409
lh_posteriorcingulate_CT_no_QC	−1.257	119	0.211	−0.037	0.029	−0.095	0.021	−0.229
lh_precentral_CT_no_QC	−0.908	119	0.366	−0.022	0.024	−0.071	0.026	−0.165
lh_precuneus_CT_no_QC	−1.801	119	0.074	−0.038	0.021	−0.080	0.004	−0.328
lh_rostralanteriorcingulate_CT_no_QC	−2.685	119	0.008	−0.119	0.044	−0.207	−0.031	−0.489
lh_rostralmiddlefrontal_CT_no_QC	−1.037	119	0.302	−0.028	0.027	−0.081	0.025	−0.189
lh_superiorfrontal_CT_no_QC	−2.490	119	0.014	−0.068	0.027	−0.123	−0.014	−0.453
lh_superiorparietal_CT_no_QC	−2.565	119	0.012	−0.062	0.024	−0.109	−0.014	−0.467
lh_superiortemporal_CT_no_QC	−2.069	119	0.041	−0.058	0.028	−0.113	−0.002	−0.377
lh_supramarginal_CT_no_QC	−2.230	119	0.028	−0.059	0.026	−0.111	−0.007	−0.406
lh_frontalpole_CT_no_QC	−2.114	119	0.037	−0.138	0.065	−0.267	−0.009	−0.385
lh_temporalpole_CT_no_QC	−0.839	119	0.403	−0.048	0.057	−0.161	0.065	−0.153
lh_transversetemporal_CT_no_QC	−2.400	119	0.018	−0.110	0.046	−0.201	−0.019	−0.437
lh_insula_CT_no_QC	0.897	119	0.371	0.026	0.029	−0.032	0.084	0.163
rh_bankssts_CT_no_QC	−1.033	119	0.304	−0.039	0.038	−0.113	0.036	−0.188
rh_caudalanteriorcingulate_CT_no_QC	−0.199	119	0.843	−0.009	0.044	−0.097	0.079	−0.036
rh_caudalmiddlefrontal_CT_no_QC	−1.910	119	0.058	−0.066	0.035	−0.135	0.002	−0.348
rh_cuneus_CT_no_QC	−2.236	119	0.027	−0.060	0.027	−0.112	−0.007	−0.407
rh_entorhinal_CT_no_QC	−1.026	119	0.307[a]	−0.063	0.061	−0.185	0.059	−0.187
rh_fusiform_CT_no_QC	−1.634	119	0.105	−0.033	0.020	−0.073	0.007	−0.298
rh_inferiorparietal_CT_no_QC	−1.812	119	0.072	−0.047	0.026	−0.099	0.004	−0.330
rh_inferiortemporal_CT_no_QC	−0.940	119	0.349	−0.028	0.030	−0.087	0.031	−0.171
rh_isthmuscingulate_CT_no_QC	−1.876	119	0.063	−0.067	0.036	−0.137	0.004	−0.342
rh_lateraloccipital_CT_no_QC	−2.792	119	0.006	−0.064	0.023	−0.110	−0.019	−0.508
rh_lateralorbitofrontal_CT_no_QC	−0.389	119	0.698	−0.011	0.029	−0.070	0.047	−0.071
rh_lingual_CT_no_QC	−2.563	119	0.012	−0.066	0.026	−0.116	−0.015	−0.467
rh_medialorbitofrontal_CT_no_QC	−2.785	119	0.006	−0.084	0.030	−0.144	−0.024	−0.507
rh_middletemporal_CT_no_QC	−1.599	119	0.113	−0.045	0.028	−0.101	0.011	−0.291
rh_parahippocampal_CT_no_QC	−3.240	119	0.002	−0.140	0.043	−0.225	−0.054	−0.590
rh_paracentral_CT_no_QC	−1.482	119	0.141	−0.036	0.024	−0.085	0.012	−0.270
rh_parsopercularis_CT_no_QC	−0.364	119	0.717	−0.008	0.023	−0.053	0.037	−0.066
rh_parsorbitalis_CT_no_QC	−1.489	119	0.139	−0.065	0.043	−0.150	0.021	−0.271
rh_parstriangularis_CT_no_QC	−1.919	119	0.057	−0.052	0.027	−0.107	0.002	−0.350
rh_pericalcarine_CT_no_QC	−2.738	119	0.007	−0.069	0.025	−0.119	−0.019	−0.499
rh_postcentral_CT_no_QC	−3.192	119	0.002[a]	−0.070	0.022	−0.113	−0.027	−0.581
rh_posteriorcingulate_CT_no_QC	−1.725	119	0.087	−0.048	0.028	−0.104	0.007	−0.314
rh_precentral_CT_no_QC	−1.068	119	0.288	−0.025	0.024	−0.072	0.022	−0.195
rh_precuneus_CT_no_QC	−1.446	119	0.151	−0.027	0.019	−0.065	0.010	−0.263
rh_rostralanteriorcingulate_CT_no_QC	−2.589	119	0.011	−0.107	0.041	−0.188	−0.025	−0.471
rh_rostralmiddlefrontal_CT_no_QC	−0.420	119	0.676	−0.010	0.025	−0.059	0.038	−0.076
rh_superiorfrontal_CT_no_QC	−3.193	119	0.002	−0.092	0.029	−0.149	−0.035	−0.581
rh_superiorparietal_CT_no_QC	−2.397	119	0.018	−0.055	0.023	−0.101	−0.010	−0.437
rh_superiortemporal_CT_no_QC	−2.340	119	0.021	−0.063	0.027	−0.115	−0.010	−0.426
rh_supramarginal_CT_no_QC	−0.792	119	0.430	−0.020	0.026	−0.071	0.030	−0.144
rh_frontalpole_CT_no_QC	−2.180	119	0.031	−0.124	0.057	−0.237	−0.011	−0.397
rh_temporalpole_CT_no_QC	−0.187	119	0.852	−0.011	0.061	−0.132	0.109	−0.034
rh_transversetemporal_CT_no_QC	−3.687	119	< .001	−0.174	0.047	−0.267	−0.081	−0.671
rh_insula_CT_no_QC	−0.419	119	0.676	−0.012	0.030	−0.071	0.047	−0.076

Note. Student's t-test.

[a] Levene's test is significant (p < .05), suggesting a violation of the equal variance assumption

Descriptives

Group Descriptives

	Group	N	Mean	SD	SE
lh_bankssts_CT_no_QC	Boy	64	2.950	0.220	0.027
	Girl	57	3.008	0.191	0.025
lh_caudalanteriorcingulate_CT_no_QC	Boy	64	2.848	0.249	0.031
	Girl	57	2.949	0.314	0.042
lh_caudalmiddlefrontal_CT_no_QC	Boy	64	2.808	0.163	0.020
	Girl	57	2.859	0.178	0.024
lh_cuneus_CT_no_QC	Boy	64	2.255	0.149	0.019
	Girl	57	2.322	0.162	0.021
lh_entorhinal_CT_no_QC	Boy	64	3.236	0.308	0.038
	Girl	57	3.285	0.315	0.042
lh_fusiform_CT_no_QC	Boy	64	3.056	0.101	0.013
	Girl	57	3.095	0.112	0.015
lh_inferiorparietal_CT_no_QC	Boy	64	2.927	0.141	0.018
	Girl	57	2.982	0.126	0.017
lh_inferiortemporal_CT_no_QC	Boy	64	3.131	0.160	0.020
	Girl	57	3.186	0.135	0.018
lh_isthmuscingulate_CT_no_QC	Boy	64	2.652	0.160	0.020
	Girl	57	2.743	0.200	0.027
lh_lateraloccipital_CT_no_QC	Boy	64	2.485	0.109	0.014
	Girl	57	2.527	0.131	0.017
lh_lateralorbitofrontal_CT_no_QC	Boy	64	3.085	0.144	0.018
	Girl	57	3.083	0.162	0.022
lh_lingual_CT_no_QC	Boy	64	2.386	0.129	0.016
	Girl	57	2.444	0.161	0.021
lh_medialorbitofrontal_CT_no_QC	Boy	64	2.832	0.179	0.022
	Girl	57	2.878	0.211	0.028
lh_middletemporal_CT_no_QC	Boy	64	3.223	0.175	0.022
	Girl	57	3.278	0.139	0.018
lh_parahippocampal_CT_no_QC	Boy	64	2.950	0.280	0.035
	Girl	57	3.102	0.268	0.035
lh_paracentral_CT_no_QC	Boy	64	2.818	0.151	0.019
	Girl	57	2.869	0.145	0.019
lh_parsopercularis_CT_no_QC	Boy	64	2.989	0.129	0.016
	Girl	57	3.009	0.115	0.015
lh_parsorbitalis_CT_no_QC	Boy	64	3.225	0.256	0.032
	Girl	57	3.282	0.215	0.028
lh_parstriangularis_CT_no_QC	Boy	64	2.989	0.167	0.021
	Girl	57	3.026	0.166	0.022
lh_pericalcarine_CT_no_QC	Boy	64	1.856	0.146	0.018
	Girl	57	1.902	0.157	0.021
lh_postcentral_CT_no_QC	Boy	64	2.380	0.110	0.014
	Girl	57	2.430	0.137	0.018
lh_posteriorcingulate_CT_no_QC	Boy	64	2.819	0.169	0.021
	Girl	57	2.856	0.150	0.020
lh_precentral_CT_no_QC	Boy	64	2.750	0.133	0.017
	Girl	57	2.772	0.136	0.018
lh_precuneus_CT_no_QC	Boy	64	2.885	0.110	0.014
	Girl	57	2.923	0.124	0.016
lh_rostralanteriorcingulate_CT_no_QC	Boy	64	3.088	0.229	0.029
	Girl	57	3.207	0.260	0.034
lh_rostralmiddlefrontal_CT_no_QC	Boy	64	2.850	0.140	0.017
	Girl	57	2.878	0.156	0.021
lh_superiorfrontal_CT_no_QC	Boy	64	3.093	0.139	0.017
	Girl	57	3.162	0.163	0.022
lh_superiorparietal_CT_no_QC	Boy	64	2.554	0.128	0.016
	Girl	57	2.616	0.137	0.018
lh_superiortemporal_CT_no_QC	Boy	64	3.190	0.159	0.020
	Girl	57	3.247	0.145	0.019
lh_supramarginal_CT_no_QC	Boy	64	2.981	0.150	0.019
	Girl	57	3.040	0.139	0.018
lh_frontalpole_CT_no_QC	Boy	64	3.324	0.361	0.045
	Girl	57	3.462	0.355	0.047
lh_temporalpole_CT_no_QC	Boy	64	3.561	0.297	0.037
	Girl	57	3.609	0.333	0.044
lh_transversetemporal_CT_no_QC	Boy	64	2.800	0.251	0.031
	Girl	57	2.910	0.254	0.034
lh_insula_CT_no_QC	Boy	64	3.236	0.141	0.018
	Girl	57	3.210	0.181	0.024
rh_bankssts_CT_no_QC	Boy	64	3.053	0.208	0.026
	Girl	57	3.091	0.204	0.027
rh_caudalanteriorcingulate_CT_no_QC	Boy	64	2.779	0.249	0.031
	Girl	57	2.788	0.237	0.031
rh_caudalmiddlefrontal_CT_no_QC	Boy	64	2.785	0.198	0.025
	Girl	57	2.852	0.183	0.024
rh_cuneus_CT_no_QC	Boy	64	2.305	0.139	0.017

Group Descriptives

	Group	N	Mean	SD	SE
	Girl	57	2.364	0.154	0.020
rh_entorhinal_CT_no_QC	Boy	64	3.398	0.386	0.048
	Girl	57	3.461	0.271	0.036
rh_fusiform_CT_no_QC	Boy	64	3.045	0.107	0.013
	Girl	57	3.079	0.116	0.015
rh_inferiorparietal_CT_no_QC	Boy	64	2.908	0.146	0.018
	Girl	57	2.956	0.140	0.019
rh_inferiortemporal_CT_no_QC	Boy	64	3.109	0.167	0.021
	Girl	57	3.137	0.162	0.021
rh_isthmuscingulate_CT_no_QC	Boy	64	2.674	0.185	0.023
	Girl	57	2.741	0.207	0.027
rh_lateraloccipital_CT_no_QC	Boy	64	2.554	0.129	0.016
	Girl	57	2.618	0.122	0.016
rh_lateralorbitofrontal_CT_no_QC	Boy	64	3.039	0.168	0.021
	Girl	57	3.050	0.155	0.020
rh_lingual_CT_no_QC	Boy	64	2.409	0.128	0.016
	Girl	57	2.475	0.153	0.020
rh_medialorbitofrontal_CT_no_QC	Boy	64	2.915	0.170	0.021
	Girl	57	2.999	0.161	0.021
rh_middletemporal_CT_no_QC	Boy	64	3.267	0.157	0.020
	Girl	57	3.312	0.151	0.020
rh_parahippocampal_CT_no_QC	Boy	64	2.829	0.223	0.028
	Girl	57	2.969	0.251	0.033
rh_paracentral_CT_no_QC	Boy	64	2.845	0.140	0.018
	Girl	57	2.881	0.127	0.017
rh_parsopercularis_CT_no_QC	Boy	64	3.010	0.122	0.015
	Girl	57	3.018	0.128	0.017
rh_parsorbitalis_CT_no_QC	Boy	64	3.237	0.222	0.028
	Girl	57	3.301	0.254	0.034
rh_parstriangularis_CT_no_QC	Boy	64	3.018	0.146	0.018
	Girl	57	3.071	0.155	0.020
rh_pericalcarine_CT_no_QC	Boy	64	1.818	0.127	0.016
	Girl	57	1.888	0.151	0.020
rh_postcentral_CT_no_QC	Boy	64	2.353	0.104	0.013
	Girl	57	2.423	0.136	0.018
rh_posteriorcingulate_CT_no_QC	Boy	64	2.767	0.169	0.021
	Girl	57	2.816	0.134	0.018
rh_precentral_CT_no_QC	Boy	64	2.705	0.114	0.014
	Girl	57	2.731	0.147	0.019
rh_precuneus_CT_no_QC	Boy	64	2.877	0.097	0.012
	Girl	57	2.904	0.111	0.015
rh_rostralanteriorcingulate_CT_no_QC	Boy	64	3.142	0.235	0.029
	Girl	57	3.248	0.215	0.029
rh_rostralmiddlefrontal_CT_no_QC	Boy	64	2.871	0.123	0.015
	Girl	57	2.881	0.146	0.019
rh_superiorfrontal_CT_no_QC	Boy	64	3.068	0.150	0.019
	Girl	57	3.160	0.166	0.022
rh_superiorparietal_CT_no_QC	Boy	64	2.546	0.121	0.015
	Girl	57	2.601	0.133	0.018
rh_superiortemporal_CT_no_QC	Boy	64	3.275	0.149	0.019
	Girl	57	3.338	0.144	0.019
rh_supramarginal_CT_no_QC	Boy	64	2.980	0.137	0.017
	Girl	57	3.000	0.145	0.019
rh_frontalpole_CT_no_QC	Boy	64	3.268	0.308	0.039
	Girl	57	3.392	0.317	0.042
rh_temporalpole_CT_no_QC	Boy	64	3.708	0.309	0.039
	Girl	57	3.719	0.362	0.048
rh_transversetemporal_CT_no_QC	Boy	64	2.826	0.262	0.033
	Girl	57	3.000	0.255	0.034
rh_insula_CT_no_QC	Boy	64	3.228	0.160	0.020
	Girl	57	3.241	0.167	0.022

Sex Differences - Surface Area - Quality Controlled

Independent Samples T-Test

	t	df	p	Mean Difference	SE Difference	95% CI for Mean Difference		Cohen's d
						Lower	Upper	
lh_bankssts_SA_QC	1.730	93	0.087	65.050	37.596	−9.609	139.708	0.357
lh_caudalanteriorcingulate_SA_QC	0.942	94	0.349	20.131	21.376	−22.311	62.573	0.193
lh_caudalmiddlefrontal_SA_QC	1.657	114	0.100	114.189	68.904	−22.309	250.687	0.308
lh_cuneus_SA_QC	4.028	91	< .001	171.989	42.703	87.164	256.814	0.838
lh_entorhinal_SA_QC	1.287	119	0.200	22.228	17.267	−11.962	56.419	0.234
lh_fusiform_SA_QC	3.536	118	< .001	236.429	66.859	104.030	368.827	0.647
lh_inferiorparietal_SA_QC	3.265	100	0.001	461.051	141.204	180.905	741.197	0.648
lh_inferiortemporal_SA_QC	2.845	103	0.005[a]	254.496	89.445	77.103	431.888	0.555
lh_isthmuscingulate_SA_QC	4.999	118	< .001	131.225	26.248	79.247	183.204	0.915
lh_lateraloccipital_SA_QC	4.076	103	< .001	543.511	133.357	279.029	807.993	0.796
lh_lateralorbitofrontal_SA_QC	3.073	111	0.003	165.290	53.795	58.691	271.889	0.581
lh_lingual_SA_QC	3.389	98	0.001	292.545	86.320	121.246	463.844	0.681
lh_medialorbitofrontal_SA_QC	3.302	93	0.001	153.754	46.568	61.278	246.230	0.681
lh_middletemporal_SA_QC	3.104	75	0.003	263.657	84.954	94.420	432.895	0.710
lh_parahippocampal_SA_QC	1.285	118	0.201	19.799	15.413	−10.723	50.322	0.235
lh_paracentral_SA_QC	1.909	114	0.059	62.275	32.619	−2.344	126.894	0.355
lh_parsopercularis_SA_QC	3.191	118	0.002	146.119	45.793	55.436	236.802	0.583
lh_parsorbitalis_SA_QC	4.135	118	< .001	67.281	16.269	35.063	99.498	0.756
lh_parstriangularis_SA_QC	4.488	118	< .001[a]	173.916	38.751	97.178	250.654	0.820
lh_pericalcarine_SA_QC	2.920	88	0.004	131.831	45.140	42.125	221.537	0.618
lh_postcentral_SA_QC	2.777	93	0.007	235.708	84.883	67.147	404.268	0.570
lh_posteriorcingulate_SA_QC	2.945	117	0.004	96.135	32.638	31.496	160.773	0.541
lh_precentral_SA_QC	4.509	103	< .001	367.680	81.545	205.954	529.407	0.885
lh_precuneus_SA_QC	3.950	108	< .001	399.219	101.057	198.907	599.531	0.754
lh_rostralanteriorcingulate_SA_QC	3.016	104	0.003	86.077	28.541	29.479	142.675	0.588
lh_rostralmiddlefrontal_SA_QC	4.387	117	< .001	639.250	145.716	350.668	927.832	0.806
lh_superiorfrontal_SA_QC	4.141	79	< .001	784.898	189.539	407.630	1162.167	0.922
lh_superiorparietal_SA_QC	2.229	91	0.028	429.183	192.577	46.652	811.714	0.464
lh_superiortemporal_SA_QC	4.017	75	< .001	354.776	88.327	178.821	530.732	0.919
lh_supramarginal_SA_QC	3.609	84	< .001	589.221	163.285	264.511	913.930	0.779
lh_frontalpole_SA_QC	2.209	117	0.029	11.919	5.397	1.231	22.608	0.405
lh_temporalpole_SA_QC	4.374	115	< .001	41.529	9.495	22.722	60.336	0.811
lh_transversetemporal_SA_QC	3.498	119	< .001	46.246	13.220	20.069	72.423	0.637
lh_insula_SA_QC	3.290	98	0.001	149.589	45.472	59.352	239.827	0.660
rh_bankssts_SA_QC	0.664	108	0.508	17.418	26.217	−34.548	69.384	0.127
rh_caudalanteriorcingulate_SA_QC	0.746	104	0.457	20.170	27.024	−33.420	73.759	0.145
rh_caudalmiddlefrontal_SA_QC	0.774	112	0.441	55.088	71.167	−85.920	196.095	0.145
rh_cuneus_SA_QC	5.661	90	< .001	212.953	37.620	138.213	287.692	1.181
rh_entorhinal_SA_QC	2.472	118	0.015	38.436	15.547	7.648	69.224	0.452
rh_fusiform_SA_QC	5.618	119	< .001	355.396	63.261	230.133	480.658	1.023
rh_inferiorparietal_SA_QC	3.164	100	0.002	544.594	172.111	203.131	886.057	0.627
rh_inferiortemporal_SA_QC	4.555	92	< .001	426.782	93.692	240.701	612.863	0.940
rh_isthmuscingulate_SA_QC	3.156	119	0.002	90.468	28.669	33.702	147.235	0.575
rh_lateraloccipital_SA_QC	4.973	109	< .001	627.097	126.099	377.173	877.020	0.945
rh_lateralorbitofrontal_SA_QC	3.486	117	< .001	254.611	73.030	109.979	399.244	0.641
rh_lingual_SA_QC	3.632	91	< .001	298.346	82.153	135.160	461.532	0.753
rh_medialorbitofrontal_SA_QC	3.963	90	< .001	205.964	51.967	102.723	309.205	0.827
rh_middletemporal_SA_QC	2.897	72	0.005	273.680	94.471	85.356	462.004	0.680
rh_parahippocampal_SA_QC	2.265	119	0.025	31.202	13.775	3.926	58.478	0.413
rh_paracentral_SA_QC	2.838	111	0.005	102.318	36.057	30.868	173.767	0.535
rh_parsopercularis_SA_QC	0.807	115	0.421	32.316	40.026	−46.969	111.600	0.149
rh_parsorbitalis_SA_QC	4.903	118	< .001	95.007	19.379	56.632	133.382	0.896
rh_parstriangularis_SA_QC	3.518	117	< .001[a]	178.173	50.640	77.883	278.462	0.646
rh_pericalcarine_SA_QC	3.899	85	< .001	158.216	40.574	77.544	238.888	0.839
rh_postcentral_SA_QC	2.201	89	0.030	247.902	112.639	24.091	471.713	0.464
rh_posteriorcingulate_SA_QC	2.918	117	0.004	97.047	33.260	31.177	162.917	0.535
rh_precentral_SA_QC	4.062	97	< .001	366.396	90.204	187.366	545.425	0.819
rh_precuneus_SA_QC	4.356	116	< .001	435.292	99.921	237.386	633.199	0.804
rh_rostralanteriorcingulate_SA_QC	3.304	117	0.001	71.597	21.669	28.683	114.512	0.607
rh_rostralmiddlefrontal_SA_QC	4.421	114	< .001	686.766	155.344	379.031	994.501	0.822
rh_superiorfrontal_SA_QC	4.524	95	< .001	773.037	170.890	433.778	1112.296	0.920
rh_superiorparietal_SA_QC	2.851	86	0.005	458.592	160.836	138.860	778.324	0.610
rh_superiortemporal_SA_QC	1.660	81	0.101	145.730	87.814	−28.992	320.453	0.365
rh_supramarginal_SA_QC	3.661	86	< .001	433.273	118.341	198.019	668.527	0.781
rh_frontalpole_SA_QC	3.340	118	0.001	23.877	7.149	9.721	38.033	0.611
rh_temporalpole_SA_QC	4.133	116	< .001	43.077	10.422	22.434	63.719	0.763
rh_transversetemporal_SA_QC	2.796	119	0.006	25.549	9.136	7.458	43.640	0.509
rh_insula_SA_QC	3.405	91	< .001	174.708	51.313	72.782	276.634	0.706

Note. Student's t-test.

[a] Levene's test is significant (p < .05), suggesting a violation of the equal variance assumption

Descriptives

Group Descriptives

	Group	N	Mean	SD	SE
lh_bankssts_SA_QC	Boy	52	1070.096	206.263	28.603
	Girl	43	1005.047	148.342	22.622
lh_caudalanteriorcingulate_SA_QC	Boy	52	602.654	103.800	14.394
	Girl	44	582.523	105.008	15.831
lh_caudalmiddlefrontal_SA_QC	Boy	62	2567.097	398.420	50.599
	Girl	54	2452.907	334.729	45.551
lh_cuneus_SA_QC	Boy	50	1724.780	214.691	30.362
	Girl	43	1552.791	193.826	29.558
lh_entorhinal_SA_QC	Boy	64	475.141	101.086	12.636
	Girl	57	452.912	87.211	11.551
lh_fusiform_SA_QC	Boy	64	3412.250	360.617	45.077
	Girl	56	3175.821	370.773	49.547
lh_inferiorparietal_SA_QC	Boy	54	5245.259	713.876	97.146
	Girl	48	4784.208	709.480	102.405
lh_inferiortemporal_SA_QC	Boy	53	3634.226	413.581	56.810
	Girl	52	3379.731	499.695	69.295
lh_isthmuscingulate_SA_QC	Boy	64	1159.297	146.370	18.296
	Girl	56	1028.071	140.023	18.711
lh_lateraloccipital_SA_QC	Boy	54	5802.315	703.701	95.762
	Girl	51	5258.804	660.288	92.459
lh_lateralorbitofrontal_SA_QC	Boy	62	2786.565	275.046	34.931
	Girl	51	2621.275	295.768	41.416
lh_lingual_SA_QC	Boy	55	3249.145	401.825	54.182
	Girl	45	2956.600	461.068	68.732
lh_medialorbitofrontal_SA_QC	Boy	52	1990.731	222.053	30.793
	Girl	43	1836.977	230.542	35.157
lh_middletemporal_SA_QC	Boy	35	3390.371	323.132	54.619
	Girl	42	3126.714	406.762	62.765
lh_parahippocampal_SA_QC	Boy	63	658.571	83.446	10.513
	Girl	57	638.772	85.270	11.294
lh_paracentral_SA_QC	Boy	61	1504.148	154.984	19.844
	Girl	55	1441.873	195.650	26.381
lh_parsopercularis_SA_QC	Boy	63	1768.365	270.144	34.035
	Girl	57	1622.246	226.789	30.039
lh_parsorbitalis_SA_QC	Boy	63	773.333	89.304	11.251
	Girl	57	706.053	88.658	11.743
lh_parstriangularis_SA_QC	Boy	63	1511.302	233.991	29.480
	Girl	57	1337.386	184.584	24.449
lh_pericalcarine_SA_QC	Boy	49	1549.367	206.459	29.494
	Girl	41	1417.537	221.166	34.540
lh_postcentral_SA_QC	Boy	49	4640.816	419.644	59.949
	Girl	46	4405.109	406.762	59.974
lh_posteriorcingulate_SA_QC	Boy	63	1315.063	172.410	21.722
	Girl	56	1218.929	183.507	24.522
lh_precentral_SA_QC	Boy	58	5267.638	431.004	56.594
	Girl	47	4899.957	395.439	57.681
lh_precuneus_SA_QC	Boy	58	4535.103	542.864	71.282
	Girl	52	4135.885	513.406	71.197
lh_rostralanteriorcingulate_SA_QC	Boy	57	821.404	157.570	20.871
	Girl	49	735.327	132.431	18.919
lh_rostralmiddlefrontal_SA_QC	Boy	63	6692.143	802.004	101.043
	Girl	56	6052.893	783.607	104.714
lh_superiorfrontal_SA_QC	Boy	43	8212.372	927.203	141.397
	Girl	38	7427.474	755.960	122.633
lh_superiorparietal_SA_QC	Boy	50	6544.160	870.932	123.168
	Girl	43	6114.977	986.245	150.401
lh_superiortemporal_SA_QC	Boy	35	4237.943	360.025	60.855
	Girl	42	3883.167	406.154	62.671
lh_supramarginal_SA_QC	Boy	45	4674.489	824.831	122.958
	Girl	41	4085.268	672.902	105.090
lh_frontalpole_SA_QC	Boy	62	275.919	30.823	3.914
	Girl	57	264.000	27.791	3.681
lh_temporalpole_SA_QC	Boy	63	475.714	55.804	7.031
	Girl	54	434.185	45.220	6.154
lh_transversetemporal_SA_QC	Boy	64	496.281	83.537	10.442
	Girl	57	450.035	57.847	7.662
lh_insula_SA_QC	Boy	54	2448.111	237.083	32.263
	Girl	46	2298.522	213.667	31.503
rh_bankssts_SA_QC	Boy	55	933.545	146.553	19.761
	Girl	55	916.127	127.766	17.228
rh_caudalanteriorcingulate_SA_QC	Boy	53	712.132	141.712	19.466
	Girl	53	691.962	136.467	18.745
rh_caudalmiddlefrontal_SA_QC	Boy	57	2403.018	333.562	44.181
	Girl	57	2347.930	421.217	55.792
rh_cuneus_SA_QC	Boy	48	1806.771	198.208	28.609

Group Descriptives

	Group	N	Mean	SD	SE
	Girl	44	1593.818	158.309	23.866
rh_entorhinal_SA_QC	Boy	63	425.857	85.535	10.776
	Girl	57	387.421	84.508	11.193
rh_fusiform_SA_QC	Boy	64	3336.922	326.424	40.803
	Girl	57	2981.526	369.481	48.939
rh_inferiorparietal_SA_QC	Boy	50	6323.440	982.668	138.970
	Girl	52	5778.846	743.486	103.103
rh_inferiortemporal_SA_QC	Boy	48	3599.021	488.830	70.556
	Girl	46	3172.239	414.705	61.145
rh_isthmuscingulate_SA_QC	Boy	64	1057.188	162.271	20.284
	Girl	57	966.719	151.763	20.101
rh_lateraloccipital_SA_QC	Boy	58	5892.776	690.152	90.621
	Girl	53	5265.679	633.198	86.976
rh_lateralorbitofrontal_SA_QC	Boy	64	2836.375	447.400	55.925
	Girl	55	2581.764	329.070	44.372
rh_lingual_SA_QC	Boy	47	3365.085	389.061	56.750
	Girl	46	3066.739	403.175	59.445
rh_medialorbitofrontal_SA_QC	Boy	48	2089.396	245.552	35.442
	Girl	44	1883.432	252.692	38.095
rh_middletemporal_SA_QC	Boy	32	3733.156	427.494	75.571
	Girl	42	3459.476	382.716	59.054
rh_parahippocampal_SA_QC	Boy	64	635.500	73.626	9.203
	Girl	57	604.298	77.837	10.310
rh_paracentral_SA_QC	Boy	60	1658.167	195.086	25.185
	Girl	53	1555.849	186.865	25.668
rh_parsopercularis_SA_QC	Boy	61	1482.066	195.239	24.998
	Girl	56	1449.750	237.108	31.685
rh_parsorbitalis_SA_QC	Boy	63	962.270	113.682	14.323
	Girl	57	867.263	96.807	12.822
rh_parstriangularis_SA_QC	Boy	63	1764.905	316.647	39.894
	Girl	56	1586.732	220.686	29.490
rh_pericalcarine_SA_QC	Boy	47	1683.766	195.259	28.481
	Girl	40	1525.550	180.455	28.532
rh_postcentral_SA_QC	Boy	50	4565.780	543.021	76.795
	Girl	41	4317.878	524.145	81.858
rh_posteriorcingulate_SA_QC	Boy	62	1363.468	184.676	23.454
	Girl	57	1266.421	177.448	23.504
rh_precentral_SA_QC	Boy	53	5356.113	447.070	61.410
	Girl	46	4989.717	448.286	66.096
rh_precuneus_SA_QC	Boy	63	4697.365	539.532	67.975
	Girl	55	4262.073	543.671	73.309
rh_rostralanteriorcingulate_SA_QC	Boy	63	632.508	113.449	14.293
	Girl	56	560.911	122.901	16.423
rh_rostralmiddlefrontal_SA_QC	Boy	61	6960.148	848.153	108.595
	Girl	55	6273.382	821.068	110.713
rh_superiorfrontal_SA_QC	Boy	46	7930.174	803.032	118.401
	Girl	51	7157.137	872.692	122.201
rh_superiorparietal_SA_QC	Boy	48	6370.167	754.029	108.835
	Girl	40	5911.575	747.920	118.257
rh_superiortemporal_SA_QC	Boy	40	3886.800	396.833	62.745
	Girl	43	3741.070	402.441	61.372
rh_supramarginal_SA_QC	Boy	44	4166.909	536.560	80.890
	Girl	44	3733.636	572.978	86.380
rh_frontalpole_SA_QC	Boy	64	350.859	40.438	5.055
	Girl	56	326.982	37.435	5.002
rh_temporalpole_SA_QC	Boy	63	473.222	58.605	7.383
	Girl	55	430.145	53.932	7.272
rh_transversetemporal_SA_QC	Boy	64	377.672	52.840	6.605
	Girl	57	352.123	46.974	6.222
rh_insula_SA_QC	Boy	48	2382.042	249.899	36.070
	Girl	45	2207.333	244.475	36.444

Sex Differences - Surface Area - No Quality Control

Independent Samples T-Test

	t	df	p	Mean Difference	SE Difference	95% CI for Mean Difference		Cohen's d
						Lower	Upper	
lh_bankssts_SA_no_QC	0.190	119	0.850	7.786	41.031	−73.459	89.032	0.035
lh_caudalanteriorcingulate_SA_no_QC	0.163	119	0.870	3.686	22.546	−40.958	48.330	0.030
lh_caudalmiddlefrontal_SA_no_QC	2.252	119	0.026	174.232	77.352	21.066	327.397	0.410
lh_cuneus_SA_no_QC	3.785	119	< .001	146.299	38.654	69.759	222.838	0.689
lh_entorhinal_SA_no_QC	0.820	119	0.414[a]	14.489	17.673	−20.506	49.484	0.149
lh_fusiform_SA_no_QC	3.318	119	0.001	223.269	67.288	90.031	356.506	0.604
lh_inferiorparietal_SA_no_QC	3.526	119	< .001	471.313	133.669	206.635	735.991	0.642
lh_inferiortemporal_SA_no_QC	3.203	119	0.002	252.629	78.880	96.439	408.820	0.583
lh_isthmuscingulate_SA_no_QC	5.134	119	< .001	130.517	25.422	80.179	180.855	0.935
lh_lateraloccipital_SA_no_QC	4.332	119	< .001	532.438	122.919	289.045	775.830	0.789
lh_lateralorbitofrontal_SA_no_QC	3.677	119	< .001	195.925	53.286	90.413	301.436	0.670
lh_lingual_SA_no_QC	3.662	119	< .001	270.960	73.991	124.450	417.469	0.667
lh_medialorbitofrontal_SA_no_QC	3.099	119	0.002	148.763	48.011	53.697	243.828	0.564
lh_middletemporal_SA_no_QC	2.430	119	0.017	165.911	68.277	30.716	301.107	0.443
lh_parahippocampal_SA_no_QC	1.733	119	0.086	27.676	15.974	−3.953	59.306	0.316
lh_paracentral_SA_no_QC	2.270	119	0.025	82.264	36.246	10.493	154.036	0.413
lh_parsopercularis_SA_no_QC	3.162	119	0.002	158.104	49.995	59.109	257.099	0.576
lh_parsorbitalis_SA_no_QC	4.495	119	< .001	72.424	16.112	40.521	104.327	0.819
lh_parstriangularis_SA_no_QC	4.333	119	< .001	167.487	38.650	90.955	244.018	0.789
lh_pericalcarine_SA_no_QC	2.653	119	0.009	117.591	44.329	29.815	205.368	0.483
lh_postcentral_SA_no_QC	3.415	119	< .001	274.524	80.395	115.334	433.714	0.622
lh_posteriorcingulate_SA_no_QC	3.210	119	0.002	104.498	32.551	40.043	168.952	0.585
lh_precentral_SA_no_QC	4.739	119	< .001	389.762	82.253	226.892	552.632	0.863
lh_precuneus_SA_no_QC	3.977	119	< .001	403.891	101.564	202.785	604.997	0.724
lh_rostralanteriorcingulate_SA_no_QC	3.312	119	0.001	98.827	29.836	39.749	157.905	0.603
lh_rostralmiddlefrontal_SA_no_QC	4.266	119	< .001	635.674	149.004	340.632	930.715	0.777
lh_superiorfrontal_SA_no_QC	5.511	119	< .001	869.854	157.833	557.329	1182.378	1.004
lh_superiorparietal_SA_no_QC	2.989	119	0.003	521.248	174.401	175.917	866.579	0.544
lh_superiortemporal_SA_no_QC	4.337	119	< .001	336.665	77.618	182.973	490.357	0.790
lh_supramarginal_SA_no_QC	4.144	119	< .001	575.107	138.786	300.297	849.918	0.755
lh_frontalpole_SA_no_QC	2.553	119	0.012	12.652	4.956	2.839	22.465	0.465
lh_temporalpole_SA_no_QC	3.798	119	< .001	39.994	10.529	19.145	60.843	0.692
lh_transversetemporal_SA_no_QC	2.437	119	0.016	32.566	13.365	6.103	59.029	0.444
lh_insula_SA_no_QC	3.487	119	< .001	165.496	47.460	71.521	259.471	0.635
rh_bankssts_SA_no_QC	0.342	119	0.733	8.359	24.425	−40.005	56.722	0.062
rh_caudalanteriorcingulate_SA_no_QC	2.068	119	0.041	59.279	28.658	2.533	116.026	0.377
rh_caudalmiddlefrontal_SA_no_QC	1.690	119	0.094	136.428	80.706	−23.379	296.234	0.308
rh_cuneus_SA_no_QC	4.581	119	< .001	202.287	44.162	114.841	289.733	0.834
rh_entorhinal_SA_no_QC	2.750	119	0.007	39.782	14.466	11.139	68.425	0.501
rh_fusiform_SA_no_QC	5.556	119	< .001	368.966	66.408	237.472	500.461	1.012
rh_inferiorparietal_SA_no_QC	2.969	119	0.004	452.925	152.575	150.812	755.038	0.541
rh_inferiortemporal_SA_no_QC	5.143	119	< .001	417.130	81.109	256.525	577.734	0.937
rh_isthmuscingulate_SA_no_QC	3.044	119	0.003	93.435	30.690	32.666	154.204	0.554
rh_lateraloccipital_SA_no_QC	5.527	119	< .001	686.140	124.147	440.317	931.962	1.007
rh_lateralorbitofrontal_SA_no_QC	3.044	119	0.003	206.375	67.798	72.128	340.621	0.554
rh_lingual_SA_no_QC	3.122	119	0.002	261.067	83.633	95.466	426.669	0.569
rh_medialorbitofrontal_SA_no_QC	4.659	119	< .001	209.325	44.927	120.364	298.285	0.849
rh_middletemporal_SA_no_QC	3.652	119	< .001	283.547	77.635	129.822	437.272	0.665
rh_parahippocampal_SA_no_QC	2.437	119	0.016	36.492	14.973	6.843	66.140	0.444
rh_paracentral_SA_no_QC	3.214	119	0.002	117.661	36.610	45.169	190.153	0.585
rh_parsopercularis_SA_no_QC	1.390	119	0.167	57.992	41.733	−24.643	140.627	0.253
rh_parsorbitalis_SA_no_QC	4.916	119	< .001	97.203	19.773	58.050	136.356	0.895
rh_parstriangularis_SA_no_QC	3.453	119	< .001[a]	173.873	50.360	74.156	273.590	0.629
rh_pericalcarine_SA_no_QC	2.460	119	0.015	117.298	47.679	22.889	211.706	0.448
rh_postcentral_SA_no_QC	3.724	119	< .001	360.674	96.853	168.894	552.453	0.678
rh_posteriorcingulate_SA_no_QC	3.384	119	< .001	114.207	33.753	47.373	181.041	0.616
rh_precentral_SA_no_QC	4.771	119	< .001	453.283	95.007	265.161	641.406	0.869
rh_precuneus_SA_no_QC	4.094	119	< .001	410.806	100.342	212.120	609.493	0.746
rh_rostralanteriorcingulate_SA_no_QC	3.248	119	0.002	69.704	21.464	27.204	112.204	0.591
rh_rostralmiddlefrontal_SA_no_QC	4.774	119	< .001	767.263	160.711	449.038	1085.487	0.869
rh_superiorfrontal_SA_no_QC	4.773	119	< .001	765.073	160.280	447.702	1082.444	0.869
rh_superiorparietal_SA_no_QC	3.090	119	0.002	452.953	146.592	162.686	743.220	0.563
rh_superiortemporal_SA_no_QC	2.533	119	0.013	186.016	73.438	40.602	331.431	0.461
rh_supramarginal_SA_no_QC	3.592	119	< .001	393.570	109.574	176.602	610.538	0.654
rh_frontalpole_SA_no_QC	3.720	119	< .001	28.758	7.730	13.453	44.064	0.678
rh_temporalpole_SA_no_QC	2.737	119	0.007	30.507	11.146	8.436	52.578	0.498
rh_transversetemporal_SA_no_QC	3.440	119	< .001	32.181	9.356	13.656	50.707	0.626
rh_insula_SA_no_QC	4.230	119	< .001	201.678	47.683	107.261	296.096	0.770

Note. Student's t-test.

[a] Levene's test is significant (p < .05), suggesting a violation of the equal variance assumption

Descriptives

Group Descriptives

	Group	N	Mean	SD	SE
lh_bankssts_SA_no_QC	Boy	64	1094.453	227.338	28.417
	Girl	57	1086.667	222.970	29.533
lh_caudalanteriorcingulate_SA_no_QC	Boy	64	616.984	113.940	14.243
	Girl	57	613.298	134.021	17.752
lh_caudalmiddlefrontal_SA_no_QC	Boy	64	2627.828	452.857	56.607
	Girl	57	2453.596	390.663	51.745
lh_cuneus_SA_no_QC	Boy	64	1713.141	204.765	25.596
	Girl	57	1566.842	220.353	29.187
lh_entorhinal_SA_no_QC	Boy	64	466.016	109.405	13.676
	Girl	57	451.526	80.902	10.716
lh_fusiform_SA_no_QC	Boy	64	3406.813	360.212	45.027
	Girl	57	3183.544	379.605	50.280
lh_inferiorparietal_SA_no_QC	Boy	64	5335.594	750.887	93.861
	Girl	57	4864.281	714.411	94.626
lh_inferiortemporal_SA_no_QC	Boy	64	3630.875	405.393	50.674
	Girl	57	3378.246	462.319	61.236
lh_isthmuscingulate_SA_no_QC	Boy	64	1162.219	145.835	18.229
	Girl	57	1031.702	132.205	17.511
lh_lateraloccipital_SA_no_QC	Boy	64	5850.438	690.612	86.326
	Girl	57	5318.000	656.829	86.999
lh_lateralorbitofrontal_SA_no_QC	Boy	64	2797.328	271.637	33.955
	Girl	57	2601.404	314.482	41.654
lh_lingual_SA_no_QC	Boy	64	3290.328	382.156	47.770
	Girl	57	3019.368	431.787	57.192
lh_medialorbitofrontal_SA_no_QC	Boy	64	1966.219	260.195	32.524
	Girl	57	1817.456	267.412	35.420
lh_middletemporal_SA_no_QC	Boy	64	3351.578	355.261	44.408
	Girl	57	3185.667	395.819	52.428
lh_parahippocampal_SA_no_QC	Boy	64	671.922	92.684	11.585
	Girl	57	644.246	81.748	10.828
lh_paracentral_SA_no_QC	Boy	64	1529.563	191.298	23.912
	Girl	57	1447.298	207.366	27.466
lh_parsopercularis_SA_no_QC	Boy	64	1786.156	303.060	37.883
	Girl	57	1628.053	238.342	31.569
lh_parsorbitalis_SA_no_QC	Boy	64	778.688	86.871	10.859
	Girl	57	706.263	90.228	11.951
lh_parstriangularis_SA_no_QC	Boy	64	1510.609	234.138	29.267
	Girl	57	1343.123	184.476	24.434
lh_pericalcarine_SA_no_QC	Boy	64	1577.328	213.642	26.705
	Girl	57	1459.737	273.033	36.164
lh_postcentral_SA_no_QC	Boy	64	4703.875	449.732	56.216
	Girl	57	4429.351	431.905	57.207
lh_posteriorcingulate_SA_no_QC	Boy	64	1321.234	172.860	21.608
	Girl	57	1216.737	185.114	24.519
lh_precentral_SA_no_QC	Boy	64	5341.797	456.253	57.032
	Girl	57	4952.035	446.386	59.125
lh_precuneus_SA_no_QC	Boy	64	4574.031	556.411	69.551
	Girl	57	4170.140	559.071	74.051
lh_rostralanteriorcingulate_SA_no_QC	Boy	64	837.266	173.634	21.704
	Girl	57	738.439	152.031	20.137
lh_rostralmiddlefrontal_SA_no_QC	Boy	64	6737.516	842.801	105.350
	Girl	57	6101.842	789.491	104.571
lh_superiorfrontal_SA_no_QC	Boy	64	8369.906	865.280	108.160
	Girl	57	7500.053	868.139	114.988
lh_superiorparietal_SA_no_QC	Boy	64	6747.125	981.455	122.682
	Girl	57	6225.877	930.026	123.185
lh_superiortemporal_SA_no_QC	Boy	64	4291.016	436.468	54.559
	Girl	57	3954.351	414.312	54.877
lh_supramarginal_SA_no_QC	Boy	64	4765.125	800.446	100.056
	Girl	57	4190.018	716.388	94.888
lh_frontalpole_SA_no_QC	Boy	64	275.547	24.432	3.054
	Girl	57	262.895	30.031	3.978
lh_temporalpole_SA_no_QC	Boy	64	477.766	59.771	7.471
	Girl	57	437.772	55.531	7.355
lh_transversetemporal_SA_no_QC	Boy	64	484.250	80.741	10.093
	Girl	57	451.684	64.100	8.490
lh_insula_SA_no_QC	Boy	64	2533.250	281.779	35.222
	Girl	57	2367.754	234.477	31.057
rh_bankssts_SA_no_QC	Boy	64	921.938	143.135	17.892
	Girl	57	913.579	123.174	16.315
rh_caudalanteriorcingulate_SA_no_QC	Boy	64	748.297	174.223	21.778
	Girl	57	689.018	135.902	18.001
rh_caudalmiddlefrontal_SA_no_QC	Boy	64	2503.375	494.445	61.806
	Girl	57	2366.947	377.175	49.958
rh_cuneus_SA_no_QC	Boy	64	1859.234	236.180	29.522

Group Descriptives

	Group	N	Mean	SD	SE
	Girl	57	1656.947	249.389	33.032
rh_entorhinal_SA_no_QC	Boy	64	421.203	85.572	10.696
	Girl	57	381.421	71.890	9.522
rh_fusiform_SA_no_QC	Boy	64	3355.703	353.883	44.235
	Girl	57	2986.737	376.360	49.850
rh_inferiorparietal_SA_no_QC	Boy	64	6350.469	929.056	116.132
	Girl	57	5897.544	721.357	95.546
rh_inferiortemporal_SA_no_QC	Boy	64	3665.516	466.414	58.302
	Girl	57	3248.386	420.402	55.684
rh_isthmuscingulate_SA_no_QC	Boy	64	1064.031	176.258	22.032
	Girl	57	970.596	159.347	21.106
rh_lateraloccipital_SA_no_QC	Boy	64	5971.578	728.728	91.091
	Girl	57	5285.439	624.488	82.715
rh_lateralorbitofrontal_SA_no_QC	Boy	64	2816.234	398.717	49.840
	Girl	57	2609.860	340.055	45.041
rh_lingual_SA_no_QC	Boy	64	3442.594	452.588	56.573
	Girl	57	3181.526	466.551	61.796
rh_medialorbitofrontal_SA_no_QC	Boy	64	2066.500	238.078	29.760
	Girl	57	1857.175	256.023	33.911
rh_middletemporal_SA_no_QC	Boy	64	3756.828	442.475	55.309
	Girl	57	3473.281	407.286	53.946
rh_parahippocampal_SA_no_QC	Boy	64	644.141	83.097	10.387
	Girl	57	607.649	81.210	10.757
rh_paracentral_SA_no_QC	Boy	64	1684.047	191.003	23.875
	Girl	57	1566.386	211.723	28.043
rh_parsopercularis_SA_no_QC	Boy	64	1515.781	227.923	28.490
	Girl	57	1457.789	230.514	30.532
rh_parsorbitalis_SA_no_QC	Boy	64	963.203	113.648	14.206
	Girl	57	866.000	102.559	13.584
rh_parstriangularis_SA_no_QC	Boy	64	1762.750	314.228	39.278
	Girl	57	1588.877	226.708	30.028
rh_pericalcarine_SA_no_QC	Boy	64	1748.719	238.496	29.812
	Girl	57	1631.421	285.740	37.847
rh_postcentral_SA_no_QC	Boy	64	4669.516	528.268	66.033
	Girl	57	4308.842	535.748	70.962
rh_posteriorcingulate_SA_no_QC	Boy	64	1371.172	194.926	24.366
	Girl	57	1256.965	173.899	23.033
rh_precentral_SA_no_QC	Boy	64	5488.266	558.466	69.808
	Girl	57	5034.982	476.872	63.163
rh_precuneus_SA_no_QC	Boy	64	4723.719	550.495	68.812
	Girl	57	4312.912	551.470	73.044
rh_rostralanteriorcingulate_SA_no_QC	Boy	64	626.125	118.057	14.757
	Girl	57	556.421	117.622	15.579
rh_rostralmiddlefrontal_SA_no_QC	Boy	64	7062.719	916.647	114.581
	Girl	57	6295.456	842.282	111.563
rh_superiorfrontal_SA_no_QC	Boy	64	8024.406	861.801	107.725
	Girl	57	7259.333	900.168	119.230
rh_superiorparietal_SA_no_QC	Boy	64	6629.813	814.456	101.807
	Girl	57	6176.860	794.026	105.171
rh_superiortemporal_SA_no_QC	Boy	64	3968.438	415.110	51.889
	Girl	57	3782.421	389.435	51.582
rh_supramarginal_SA_no_QC	Boy	64	4290.359	624.261	78.033
	Girl	57	3896.789	575.149	76.180
rh_frontalpole_SA_no_QC	Boy	64	352.109	40.855	5.107
	Girl	57	323.351	44.161	5.849
rh_temporalpole_SA_no_QC	Boy	64	461.297	66.261	8.283
	Girl	57	430.789	54.958	7.279
rh_transversetemporal_SA_no_QC	Boy	64	380.094	55.415	6.927
	Girl	57	347.912	46.399	6.146
rh_insula_SA_no_QC	Boy	64	2473.906	269.512	33.689
	Girl	57	2272.228	252.884	33.495

Sex Differences - Volume - Quality Controlled

Independent Samples T-Test

	t	df	p	Mean Difference	SE Difference	95% CI for Mean Difference		Cohen's d
						Lower	Upper	
lh_bankssts_Vol_QC	1.523	93	0.131[a]	182.854	120.074	−55.589	421.297	0.314
lh_caudalanteriorcingulate_Vol_QC	−0.532	94	0.596	−48.685	91.507	−230.374	133.004	−0.109
lh_caudalmiddlefrontal_Vol_QC	0.953	114	0.343	220.281	231.226	−237.775	678.338	0.177
lh_cuneus_Vol_QC	2.561	91	0.012	311.165	121.488	69.844	552.486	0.533
lh_entorhinal_Vol_QC	0.817	119	0.415	57.110	69.882	−81.265	195.484	0.149
lh_fusiform_Vol_QC	2.216	118	0.029	618.775	279.255	65.774	1171.775	0.405
lh_inferiorparietal_Vol_QC	2.296	100	0.024	1087.602	473.698	147.798	2027.406	0.455
lh_inferiortemporal_Vol_QC	2.287	103	0.024	840.713	367.640	111.585	1569.841	0.446
lh_isthmuscingulate_Vol_QC	4.024	118	< .001	350.475	87.100	177.994	522.957	0.736
lh_lateraloccipital_Vol_QC	2.849	103	0.005	1210.930	425.009	368.025	2053.836	0.556
lh_lateralorbitofrontal_Vol_QC	3.159	111	0.002	593.601	187.930	221.205	965.996	0.597
lh_lingual_Vol_QC	2.551	98	0.012	635.600	249.127	141.215	1129.985	0.513
lh_medialorbitofrontal_Vol_QC	2.764	93	0.007	479.893	173.632	135.095	824.691	0.570
lh_middletemporal_Vol_QC	2.922	75	0.005	1009.757	345.594	321.299	1698.215	0.669
lh_parahippocampal_Vol_QC	−1.877	118	0.063	−108.258	57.664	−222.448	5.932	−0.343
lh_paracentral_Vol_QC	1.210	114	0.229	131.610	108.737	−83.798	347.017	0.225
lh_parsopercularis_Vol_QC	2.502	118	0.014	412.544	164.884	86.029	739.059	0.457
lh_parsorbitalis_Vol_QC	2.412	118	0.017	208.474	86.428	37.323	379.624	0.441
lh_parstriangularis_Vol_QC	3.525	118	< .001	508.341	144.216	222.753	793.928	0.644
lh_pericalcarine_Vol_QC	1.932	88	0.057	164.341	85.055	−4.688	333.371	0.409
lh_postcentral_Vol_QC	1.440	93	0.153	397.050	275.781	−150.597	944.696	0.296
lh_posteriorcingulate_Vol_QC	1.906	117	0.059	212.056	111.234	−8.237	432.348	0.350
lh_precentral_Vol_QC	3.337	103	0.001	969.227	290.455	393.178	1545.276	0.655
lh_precuneus_Vol_QC	3.019	108	0.003	1018.522	337.322	349.890	1687.153	0.577
lh_rostralanteriorcingulate_Vol_QC	1.405	104	0.163	154.513	109.968	−63.559	372.585	0.274
lh_rostralmiddlefrontal_Vol_QC	4.124	117	< .001	1919.284	465.423	997.539	2841.029	0.757
lh_superiorfrontal_Vol_QC	3.878	79	< .001[a]	2355.294	607.346	1146.401	3564.186	0.863
lh_superiorparietal_Vol_QC	0.832	91	0.408	499.882	600.881	−693.695	1693.460	0.173
lh_superiortemporal_Vol_QC	2.362	75	0.021	851.357	360.512	133.180	1569.534	0.540
lh_supramarginal_Vol_QC	2.968	84	0.004	1675.225	564.473	552.709	2797.741	0.641
lh_frontalpole_Vol_QC	−0.707	117	0.481	−34.368	48.611	−130.638	61.903	−0.130
lh_temporalpole_Vol_QC	1.909	115	0.059	143.524	75.196	−5.424	292.472	0.354
lh_transversetemporal_Vol_QC	1.943	119	0.054	91.341	47.013	−1.750	184.432	0.354
lh_insula_Vol_QC	2.277	98	0.025	381.482	167.533	49.019	713.946	0.457
rh_bankssts_Vol_QC	−0.024	108	0.981	−2.000	82.147	−164.830	160.830	−0.005
rh_caudalanteriorcingulate_Vol_QC	0.032	104	0.974	3.453	106.481	−207.703	214.608	0.006
rh_caudalmiddlefrontal_Vol_QC	0.422	112	0.674	99.632	236.316	−368.599	567.862	0.079
rh_cuneus_Vol_QC	3.207	90	0.002	435.468	135.796	165.686	705.249	0.669
rh_entorhinal_Vol_QC	2.260	118	0.026	164.774	72.911	20.390	309.159	0.413
rh_fusiform_Vol_QC	4.527	119	< .001	1147.164	253.398	645.411	1648.916	0.824
rh_inferiorparietal_Vol_QC	2.201	100	0.030	1268.868	576.486	125.135	2412.600	0.436
rh_inferiortemporal_Vol_QC	3.317	92	0.001	1330.005	400.949	533.686	2126.325	0.684
rh_isthmuscingulate_Vol_QC	2.204	119	0.029	217.652	98.760	22.097	413.206	0.401
rh_lateraloccipital_Vol_QC	3.488	109	< .001	1442.932	413.676	623.039	2262.825	0.663
rh_lateralorbitofrontal_Vol_QC	3.412	117	< .001	721.135	211.371	302.525	1139.745	0.627
rh_lingual_Vol_QC	2.247	91	0.027	578.496	257.470	67.064	1089.929	0.466
rh_medialorbitofrontal_Vol_QC	3.257	90	0.002	524.646	161.095	204.602	844.690	0.680
rh_middletemporal_Vol_QC	2.043	72	0.045	907.028	443.975	21.979	1792.077	0.479
rh_parahippocampal_Vol_QC	−0.366	119	0.715	−19.971	54.537	−127.961	88.018	−0.067
rh_paracentral_Vol_QC	2.193	111	0.030	258.581	117.913	24.928	492.233	0.413
rh_parsopercularis_Vol_QC	0.538	115	0.592	73.547	136.765	−197.358	344.452	0.100
rh_parsorbitalis_Vol_QC	3.316	118	0.001	295.023	88.961	118.856	471.189	0.606
rh_parstriangularis_Vol_QC	2.857	117	0.005[a]	519.520	181.822	159.431	879.609	0.525
rh_pericalcarine_Vol_QC	1.567	85	0.121	132.930	84.847	−35.769	301.629	0.337
rh_postcentral_Vol_QC	0.599	89	0.551	195.376	326.356	−453.086	843.838	0.126
rh_posteriorcingulate_Vol_QC	2.210	117	0.029	241.012	109.055	25.034	456.990	0.406
rh_precentral_Vol_QC	2.792	97	0.006	835.262	299.185	241.463	1429.060	0.563
rh_precuneus_Vol_QC	3.674	116	< .001	1171.676	318.910	540.034	1803.319	0.678
rh_rostralanteriorcingulate_Vol_QC	1.338	117	0.183	117.740	87.986	−56.513	291.993	0.246
rh_rostralmiddlefrontal_Vol_QC	4.441	114	< .001	2337.358	526.348	1294.666	3380.050	0.826
rh_superiorfrontal_Vol_QC	4.388	95	< .001	2533.263	577.257	1387.263	3679.263	0.892
rh_superiorparietal_Vol_QC	1.916	86	0.059	926.842	483.752	−34.825	1888.509	0.410
rh_superiortemporal_Vol_QC	0.801	81	0.425	284.933	355.624	−422.648	992.514	0.176
rh_supramarginal_Vol_QC	2.585	86	0.011	1068.273	413.200	246.857	1889.688	0.551
rh_frontalpole_Vol_QC	−0.041	118	0.967	−2.029	48.901	−98.867	94.809	−0.008
rh_temporalpole_Vol_QC	2.293	116	0.024	173.468	75.641	23.651	323.285	0.423
rh_transversetemporal_Vol_QC	0.952	119	0.343	33.695	35.380	−36.361	103.750	0.173
rh_insula_Vol_QC	2.696	91	0.008	419.781	155.718	110.466	729.095	0.559

Note. Student's t-test.

[a] Levene's test is significant (p < .05), suggesting a violation of the equal variance assumption

Descriptives

Group Descriptives

	Group	N	Mean	SD	SE
lh_bankssts_Vol_QC	Boy	52	3123.365	666.610	92.442
	Girl	43	2940.512	460.242	70.186
lh_caudalanteriorcingulate_Vol_QC	Boy	52	1847.519	452.762	62.787
	Girl	44	1896.205	439.470	66.253
lh_caudalmiddlefrontal_Vol_QC	Boy	62	8319.226	1310.011	166.372
	Girl	54	8098.944	1159.308	157.762
lh_cuneus_Vol_QC	Boy	50	4198.560	616.850	87.236
	Girl	43	3887.395	543.477	82.879
lh_entorhinal_Vol_QC	Boy	64	2033.250	402.134	50.267
	Girl	57	1976.140	361.863	47.930
lh_fusiform_Vol_QC	Boy	64	12171.328	1521.289	190.161
	Girl	56	11552.554	1531.674	204.678
lh_inferiorparietal_Vol_QC	Boy	54	17661.519	2288.866	311.475
	Girl	48	16573.917	2494.899	360.108
lh_inferiortemporal_Vol_QC	Boy	53	14341.925	1761.568	241.970
	Girl	52	13501.212	2000.203	277.378
lh_isthmuscingulate_Vol_QC	Boy	64	3631.297	480.560	60.070
	Girl	56	3280.821	470.731	62.904
lh_lateraloccipital_Vol_QC	Boy	54	16299.852	2314.471	314.960
	Girl	51	15088.922	2020.286	282.897
lh_lateralorbitofrontal_Vol_QC	Boy	62	9851.306	990.508	125.795
	Girl	51	9257.706	998.502	139.818
lh_lingual_Vol_QC	Boy	55	8490.400	1276.816	172.166
	Girl	45	7854.800	1191.857	177.672
lh_medialorbitofrontal_Vol_QC	Boy	52	6719.846	876.423	121.538
	Girl	43	6239.953	799.073	121.857
lh_middletemporal_Vol_QC	Boy	35	14427.829	1340.312	226.554
	Girl	42	13418.071	1637.447	252.664
lh_parahippocampal_Vol_QC	Boy	63	2234.619	312.907	39.423
	Girl	57	2342.877	318.225	42.150
lh_paracentral_Vol_QC	Boy	61	4752.246	545.639	69.862
	Girl	55	4620.636	625.411	84.330
lh_parsopercularis_Vol_QC	Boy	63	6285.000	927.175	116.813
	Girl	57	5872.456	873.231	115.662
lh_parsorbitalis_Vol_QC	Boy	63	3568.333	491.196	61.885
	Girl	57	3359.860	451.542	59.808
lh_parstriangularis_Vol_QC	Boy	63	5465.762	864.582	108.927
	Girl	57	4957.421	695.610	92.136
lh_pericalcarine_Vol_QC	Boy	49	2508.000	402.149	57.450
	Girl	41	2343.659	401.503	62.704
lh_postcentral_Vol_QC	Boy	49	12841.571	1225.273	175.039
	Girl	46	12444.522	1458.746	215.080
lh_posteriorcingulate_Vol_QC	Boy	63	4190.556	547.682	69.001
	Girl	56	3978.500	664.980	88.862
lh_precentral_Vol_QC	Boy	58	16370.121	1477.577	194.015
	Girl	47	15400.894	1482.888	216.301
lh_precuneus_Vol_QC	Boy	58	14588.310	1780.770	233.827
	Girl	52	13569.788	1749.986	242.679
lh_rostralanteriorcingulate_Vol_QC	Boy	57	2916.982	549.349	72.763
	Girl	49	2762.469	581.642	83.092
lh_rostralmiddlefrontal_Vol_QC	Boy	63	23544.587	2585.092	325.691
	Girl	56	21625.304	2475.543	330.808
lh_superiorfrontal_Vol_QC	Boy	43	31218.767	3083.009	470.154
	Girl	38	28863.474	2257.977	366.292
lh_superiorparietal_Vol_QC	Boy	50	19549.580	2606.139	368.564
	Girl	43	19049.698	3187.684	486.117
lh_superiortemporal_Vol_QC	Boy	35	16294.571	1485.764	251.140
	Girl	42	15443.214	1645.666	253.932
lh_supramarginal_Vol_QC	Boy	45	16300.444	2790.729	416.017
	Girl	41	14625.220	2405.828	375.727
lh_frontalpole_Vol_QC	Boy	62	1485.790	270.146	34.309
	Girl	57	1520.158	259.076	34.315
lh_temporalpole_Vol_QC	Boy	63	2607.635	441.752	55.656
	Girl	54	2464.111	358.413	48.774
lh_transversetemporal_Vol_QC	Boy	64	1561.797	272.497	34.062
	Girl	57	1470.456	240.969	31.917
lh_insula_Vol_QC	Boy	54	7885.852	856.617	116.571
	Girl	46	7504.370	808.754	119.244
rh_bankssts_Vol_QC	Boy	55	2743.764	478.536	64.526
	Girl	55	2745.764	377.029	50.839
rh_caudalanteriorcingulate_Vol_QC	Boy	53	2314.528	520.054	71.435
	Girl	53	2311.075	574.862	78.963
rh_caudalmiddlefrontal_Vol_QC	Boy	57	7854.123	1127.423	149.331
	Girl	57	7754.491	1382.790	183.155
rh_cuneus_Vol_QC	Boy	48	4665.854	722.905	104.342

Group Descriptives

	Group	N	Mean	SD	SE
	Girl	44	4230.386	561.098	84.589
rh_entorhinal_Vol_QC	Boy	63	2017.476	388.266	48.917
	Girl	57	1852.702	410.255	54.340
rh_fusiform_Vol_QC	Boy	64	11981.953	1363.310	170.414
	Girl	57	10834.789	1422.241	188.380
rh_inferiorparietal_Vol_QC	Boy	50	21676.560	3266.954	462.017
	Girl	52	20407.692	2521.109	349.615
rh_inferiortemporal_Vol_QC	Boy	48	13959.875	2057.075	296.913
	Girl	46	12629.870	1816.729	267.862
rh_isthmuscingulate_Vol_QC	Boy	64	3448.266	546.753	68.344
	Girl	57	3230.614	537.184	71.152
rh_lateraloccipital_Vol_QC	Boy	58	17240.121	2331.957	306.201
	Girl	53	15797.189	1993.270	273.797
rh_lateralorbitofrontal_Vol_QC	Boy	64	9612.953	1255.710	156.964
	Girl	55	8891.818	1011.818	136.433
rh_lingual_Vol_QC	Boy	47	9027.170	1304.258	190.246
	Girl	46	8448.674	1173.679	173.050
rh_medialorbitofrontal_Vol_QC	Boy	48	7282.396	767.934	110.842
	Girl	44	6757.750	776.120	117.004
rh_middletemporal_Vol_QC	Boy	32	15378.719	1992.840	352.288
	Girl	42	14471.690	1812.206	279.629
rh_parahippocampal_Vol_QC	Boy	64	2075.766	271.596	33.949
	Girl	57	2095.737	327.977	43.442
rh_paracentral_Vol_QC	Boy	60	5230.033	614.438	79.324
	Girl	53	4971.453	637.843	87.615
rh_parsopercularis_Vol_QC	Boy	61	5161.672	696.440	89.170
	Girl	56	5088.125	782.781	104.603
rh_parsorbitalis_Vol_QC	Boy	63	4233.286	513.409	64.683
	Girl	57	3938.263	455.188	60.291
rh_parstriangularis_Vol_QC	Boy	63	6377.127	1136.387	143.171
	Girl	56	5857.607	793.237	106.001
rh_pericalcarine_Vol_QC	Boy	47	2656.830	408.515	59.588
	Girl	40	2523.900	377.114	59.627
rh_postcentral_Vol_QC	Boy	50	12139.400	1477.819	208.995
	Girl	41	11944.024	1631.942	254.866
rh_posteriorcingulate_Vol_QC	Boy	62	4367.468	594.822	75.543
	Girl	57	4126.456	593.731	78.642
rh_precentral_Vol_QC	Boy	53	16133.566	1383.776	190.076
	Girl	46	15298.304	1593.379	234.931
rh_precuneus_Vol_QC	Boy	63	14982.349	1773.277	223.412
	Girl	55	13810.673	1674.819	225.833
rh_rostralanteriorcingulate_Vol_QC	Boy	63	2283.222	484.850	61.085
	Girl	56	2165.482	472.488	63.139
rh_rostralmiddlefrontal_Vol_QC	Boy	61	24531.049	2902.661	371.648
	Girl	55	22193.691	2748.486	370.606
rh_superiorfrontal_Vol_QC	Boy	46	30209.478	2540.895	374.635
	Girl	51	27676.216	3082.538	431.642
rh_superiorparietal_Vol_QC	Boy	48	18945.167	2166.948	312.772
	Girl	40	18018.325	2366.448	374.168
rh_superiortemporal_Vol_QC	Boy	40	15145.375	1732.256	273.894
	Girl	43	14860.442	1505.995	229.662
rh_supramarginal_Vol_QC	Boy	44	14315.705	1832.003	276.185
	Girl	44	13247.432	2038.649	307.338
rh_frontalpole_Vol_QC	Boy	64	1791.578	274.387	34.298
	Girl	56	1793.607	258.829	34.588
rh_temporalpole_Vol_QC	Boy	63	2667.032	400.571	50.467
	Girl	55	2493.564	420.337	56.678
rh_transversetemporal_Vol_QC	Boy	64	1252.484	201.765	25.221
	Girl	57	1218.789	185.461	24.565
rh_insula_Vol_QC	Boy	48	7658.292	776.349	112.056
	Girl	45	7238.511	721.765	107.594

Sex Differences - Volume - No Quality Control

Independent Samples T-Test

	t	df	p	Mean Difference	SE Difference	95% CI for Mean Difference		Cohen's d
						Lower	Upper	
lh_bankssts_Vol_no_QC	0.034	119	0.973	4.354	129.289	−251.651	260.359	0.006
lh_caudalanteriorcingulate_Vol_no_QC	−0.881	119	0.380	−79.636	90.423	−258.683	99.411	−0.160
lh_caudalmiddlefrontal_Vol_no_QC	1.221	119	0.224	295.618	242.060	−183.686	774.922	0.222
lh_cuneus_Vol_no_QC	1.999	119	0.048	225.903	112.984	2.183	449.622	0.364
lh_entorhinal_Vol_no_QC	0.526	119	0.600	41.040	77.948	−113.306	195.385	0.096
lh_fusiform_Vol_no_QC	1.932	119	0.056	551.173	285.226	−13.603	1115.949	0.352
lh_inferiorparietal_Vol_no_QC	2.584	119	0.011	1090.296	422.009	254.675	1925.916	0.471
lh_inferiortemporal_Vol_no_QC	1.902	119	0.060	653.867	343.734	−26.762	1334.495	0.346
lh_isthmuscingulate_Vol_no_QC	4.012	119	< .001	345.552	86.134	174.999	516.105	0.731
lh_lateraloccipital_Vol_no_QC	2.996	119	0.003	1138.341	379.959	385.984	1890.699	0.546
lh_lateralorbitofrontal_Vol_no_QC	3.511	119	< .001	634.880	180.835	276.809	992.952	0.639
lh_lingual_Vol_no_QC	2.367	119	0.020	513.863	217.133	83.917	943.808	0.431
lh_medialorbitofrontal_Vol_no_QC	3.058	119	0.003	455.055	148.812	160.392	749.717	0.557
lh_middletemporal_Vol_no_QC	1.268	119	0.207	386.720	304.968	−217.148	990.587	0.231
lh_parahippocampal_Vol_no_QC	−1.045	119	0.298	−65.922	63.069	−190.805	58.960	−0.190
lh_paracentral_Vol_no_QC	1.118	119	0.266	133.153	119.079	−102.636	368.942	0.204
lh_parsopercularis_Vol_no_QC	2.540	119	0.012	454.895	179.111	100.238	809.551	0.463
lh_parsorbitalis_Vol_no_QC	2.650	119	0.009	235.667	88.920	59.596	411.738	0.483
lh_parstriangularis_Vol_no_QC	3.452	119	< .001	487.798	141.308	207.995	767.601	0.629
lh_pericalcarine_Vol_no_QC	1.442	119	0.152	121.331	84.144	−45.283	287.945	0.263
lh_postcentral_Vol_no_QC	1.695	119	0.093	413.312	243.879	−69.594	896.217	0.309
lh_posteriorcingulate_Vol_no_QC	2.163	119	0.033	240.336	111.096	20.354	460.318	0.394
lh_precentral_Vol_no_QC	3.235	119	0.002	882.399	272.729	342.369	1422.429	0.589
lh_precuneus_Vol_no_QC	2.965	119	0.004	1009.018	340.305	335.180	1682.856	0.540
lh_rostralanteriorcingulate_Vol_no_QC	1.701	119	0.092	190.609	112.048	−31.258	412.475	0.310
lh_rostralmiddlefrontal_Vol_no_QC	3.878	119	< .001	1823.528	470.189	892.506	2754.549	0.706
lh_superiorfrontal_Vol_no_QC	5.080	119	< .001	2440.908	480.529	1489.413	3392.403	0.925
lh_superiorparietal_Vol_no_QC	1.988	119	0.049	1008.970	507.634	3.805	2014.135	0.362
lh_superiortemporal_Vol_no_QC	2.425	119	0.017	728.657	300.538	133.561	1323.752	0.442
lh_supramarginal_Vol_no_QC	3.514	119	< .001	1610.887	458.422	703.165	2518.609	0.640
lh_frontalpole_Vol_no_QC	−0.582	119	0.561	−29.175	50.096	−128.371	70.020	−0.106
lh_temporalpole_Vol_no_QC	1.204	119	0.231	96.808	80.372	−62.337	255.953	0.219
lh_transversetemporal_Vol_no_QC	1.334	119	0.185	63.840	47.855	−30.918	158.598	0.243
lh_insula_Vol_no_QC	3.417	119	< .001	549.947	160.966	231.219	868.676	0.622
rh_bankssts_Vol_no_QC	−0.147	119	0.883	−11.831	80.228	−170.691	147.029	−0.027
rh_caudalanteriorcingulate_Vol_no_QC	1.294	119	0.198	141.361	109.271	−75.007	357.730	0.236
rh_caudalmiddlefrontal_Vol_no_QC	0.111	119	0.912	28.284	254.731	−476.109	532.676	0.020
rh_cuneus_Vol_no_QC	2.760	119	0.007	383.930	139.110	108.478	659.382	0.503
rh_entorhinal_Vol_no_QC	2.096	119	0.038	150.936	72.015	8.340	293.532	0.382
rh_fusiform_Vol_no_QC	4.596	119	< .001	1190.258	258.974	677.463	1703.054	0.837
rh_inferiorparietal_Vol_no_QC	2.221	119	0.028	1134.293	510.820	122.818	2145.768	0.404
rh_inferiortemporal_Vol_no_QC	3.632	119	< .001	1296.829	357.056	589.823	2003.835	0.661
rh_isthmuscingulate_Vol_no_QC	2.041	119	0.043	211.331	103.531	6.330	416.332	0.372
rh_lateraloccipital_Vol_no_QC	3.791	119	< .001	1517.554	400.268	724.983	2310.125	0.690
rh_lateralorbitofrontal_Vol_no_QC	2.966	119	0.004	597.175	201.348	198.487	995.863	0.540
rh_lingual_Vol_no_QC	1.635	119	0.105	412.954	252.577	−87.174	913.083	0.298
rh_medialorbitofrontal_Vol_no_QC	3.747	119	< .001	542.853	144.880	255.977	829.730	0.682
rh_middletemporal_Vol_no_QC	2.135	119	0.035	755.850	354.059	54.778	1456.922	0.389
rh_parahippocampal_Vol_no_QC	−0.274	119	0.785	−16.520	60.333	−135.986	102.947	−0.050
rh_paracentral_Vol_no_QC	2.400	119	0.018	285.027	118.744	49.902	520.153	0.437
rh_parsopercularis_Vol_no_QC	0.929	119	0.355	129.556	139.423	−146.516	405.627	0.169
rh_parsorbitalis_Vol_no_QC	3.217	119	0.002	285.346	88.696	109.719	460.974	0.586
rh_parstriangularis_Vol_no_QC	2.761	119	0.007[a]	491.717	178.086	139.088	844.345	0.503
rh_pericalcarine_Vol_no_QC	0.887	119	0.377	85.994	96.987	−106.050	278.037	0.161
rh_postcentral_Vol_no_QC	1.805	119	0.074	504.414	279.490	−49.005	1057.832	0.329
rh_posteriorcingulate_Vol_no_QC	2.299	119	0.023	253.041	110.067	35.097	470.984	0.419
rh_precentral_Vol_no_QC	3.908	119	< .001	1094.055	279.952	539.721	1648.388	0.712
rh_precuneus_Vol_no_QC	3.276	119	0.001	1049.834	320.446	415.318	1684.350	0.597
rh_rostralanteriorcingulate_Vol_no_QC	1.357	119	0.177	120.885	89.075	−55.493	297.263	0.247
rh_rostralmiddlefrontal_Vol_no_QC	4.677	119	< .001	2459.662	525.901	1418.325	3500.999	0.852
rh_superiorfrontal_Vol_no_QC	3.547	119	< .001	1787.903	504.047	789.839	2785.967	0.646
rh_superiorparietal_Vol_no_QC	1.913	119	0.058	814.031	425.460	−28.423	1656.484	0.348
rh_superiortemporal_Vol_no_QC	0.645	119	0.520	180.975	280.482	−374.408	736.358	0.118
rh_supramarginal_Vol_no_QC	2.723	119	0.007	1027.996	377.555	280.400	1775.593	0.496
rh_frontalpole_Vol_no_QC	0.259	119	0.796	12.797	49.470	−85.158	110.752	0.047
rh_temporalpole_Vol_no_QC	1.290	119	0.200	102.197	79.237	−54.700	259.095	0.235
rh_transversetemporal_Vol_no_QC	0.828	119	0.409	30.316	36.593	−42.142	102.773	0.151
rh_insula_Vol_no_QC	4.140	119	< .001	619.379	149.597	323.162	915.595	0.754

Note. Student's t-test.

[a] Levene's test is significant ($p < .05$), suggesting a violation of the equal variance assumption

Descriptives

Group Descriptives

	Group	N	Mean	SD	SE
lh_bankssts_Vol_no_QC	Boy	64	3230.547	732.031	91.504
	Girl	57	3226.193	684.145	90.617
lh_caudalanteriorcingulate_Vol_no_QC	Boy	64	1947.609	485.986	60.748
	Girl	57	2027.246	508.059	67.294
lh_caudalmiddlefrontal_Vol_no_QC	Boy	64	8258.109	1374.637	171.830
	Girl	57	7962.491	1275.935	169.002
lh_cuneus_Vol_no_QC	Boy	64	4180.078	590.091	73.761
	Girl	57	3954.175	652.759	86.460
lh_entorhinal_Vol_no_QC	Boy	64	2024.391	481.851	60.231
	Girl	57	1983.351	357.852	47.399
lh_fusiform_Vol_no_QC	Boy	64	12167.875	1517.726	189.716
	Girl	57	11616.702	1618.825	214.419
lh_inferiorparietal_Vol_no_QC	Boy	64	17794.594	2265.026	283.128
	Girl	57	16704.298	2374.450	314.504
lh_inferiortemporal_Vol_no_QC	Boy	64	14087.516	1862.591	232.824
	Girl	57	13433.649	1914.867	253.630
lh_isthmuscingulate_Vol_no_QC	Boy	64	3644.078	490.423	61.303
	Girl	57	3298.526	452.469	59.931
lh_lateraloccipital_Vol_no_QC	Boy	64	16302.078	2211.218	276.402
	Girl	57	15163.737	1936.104	256.443
lh_lateralorbitofrontal_Vol_no_QC	Boy	64	9811.688	970.148	121.268
	Girl	57	9176.807	1017.945	134.830
lh_lingual_Vol_no_QC	Boy	64	8578.547	1177.336	147.167
	Girl	57	8064.684	1208.773	160.106
lh_medialorbitofrontal_Vol_no_QC	Boy	64	6619.125	865.634	108.204
	Girl	57	6164.070	758.785	100.504
lh_middletemporal_Vol_no_QC	Boy	64	13970.141	1783.839	222.980
	Girl	57	13583.421	1542.295	204.282
lh_parahippocampal_Vol_no_QC	Boy	64	2288.797	370.564	46.320
	Girl	57	2354.719	316.784	41.959
lh_paracentral_Vol_no_QC	Boy	64	4775.609	616.927	77.116
	Girl	57	4642.456	693.020	91.793
lh_parsopercularis_Vol_no_QC	Boy	64	6346.000	1039.080	129.885
	Girl	57	5891.105	916.859	121.441
lh_parsorbitalis_Vol_no_QC	Boy	64	3581.281	518.264	64.783
	Girl	57	3345.614	452.092	59.881
lh_parstriangularis_Vol_no_QC	Boy	64	5464.359	838.244	104.781
	Girl	57	4976.561	699.127	92.602
lh_pericalcarine_Vol_no_QC	Boy	64	2563.594	405.305	50.663
	Girl	57	2442.263	518.457	68.671
lh_postcentral_Vol_no_QC	Boy	64	12793.891	1306.363	163.295
	Girl	57	12380.579	1374.979	182.120
lh_posteriorcingulate_Vol_no_QC	Boy	64	4218.406	550.839	68.855
	Girl	57	3978.070	670.356	88.791
lh_precentral_Vol_no_QC	Boy	64	16275.750	1489.959	186.245
	Girl	57	15393.351	1505.928	199.465
lh_precuneus_Vol_no_QC	Boy	64	14672.422	1783.974	222.997
	Girl	57	13663.404	1959.326	259.519
lh_rostralanteriorcingulate_Vol_no_QC	Boy	64	3005.188	602.884	75.361
	Girl	57	2814.579	628.836	83.291
lh_rostralmiddlefrontal_Vol_no_QC	Boy	64	23607.703	2673.996	334.250
	Girl	57	21784.175	2473.773	327.659
lh_superiorfrontal_Vol_no_QC	Boy	64	31144.609	2712.887	339.111
	Girl	57	28703.702	2552.184	338.045
lh_superiorparietal_Vol_no_QC	Boy	64	19586.672	2678.947	334.868
	Girl	57	18577.702	2904.386	384.695
lh_superiortemporal_Vol_no_QC	Boy	64	16355.938	1720.000	215.000
	Girl	57	15627.281	1567.946	207.679
lh_supramarginal_Vol_no_QC	Boy	64	16479.063	2696.192	337.024
	Girl	57	14868.175	2299.004	304.511
lh_frontalpole_Vol_no_QC	Boy	64	1475.141	259.728	32.466
	Girl	57	1504.316	291.362	38.592
lh_temporalpole_Vol_no_QC	Boy	64	2612.563	462.813	57.852
	Girl	57	2515.754	415.786	55.072
lh_transversetemporal_Vol_no_QC	Boy	64	1537.734	270.379	33.797
	Girl	57	1473.895	253.921	33.633
lh_insula_Vol_no_QC	Boy	64	8174.000	982.591	122.824
	Girl	57	7624.053	757.482	100.331
rh_bankssts_Vol_no_QC	Boy	64	2733.906	477.819	59.727
	Girl	57	2745.737	394.357	52.234
rh_caudalanteriorcingulate_Vol_no_QC	Boy	64	2460.344	633.607	79.201
	Girl	57	2318.982	559.755	74.141
rh_caudalmiddlefrontal_Vol_no_QC	Boy	64	7677.547	1497.922	187.240
	Girl	57	7649.263	1277.836	169.253
rh_cuneus_Vol_no_QC	Boy	64	4805.000	758.963	94.870

Group Descriptives

	Group	N	Mean	SD	SE
	Girl	57	4421.070	769.259	101.891
rh_entorhinal_Vol_no_QC	Boy	64	1971.094	430.584	53.823
	Girl	57	1820.158	351.676	46.581
rh_fusiform_Vol_no_QC	Boy	64	11981.609	1438.693	179.837
	Girl	57	10791.351	1402.926	185.822
rh_inferiorparietal_Vol_no_QC	Boy	64	21516.188	3089.942	386.243
	Girl	57	20381.895	2444.590	323.794
rh_inferiortemporal_Vol_no_QC	Boy	64	13925.250	2096.734	262.092
	Girl	57	12628.421	1794.959	237.748
rh_isthmuscingulate_Vol_no_QC	Boy	64	3468.734	572.118	71.515
	Girl	57	3257.404	564.327	74.747
rh_lateraloccipital_Vol_no_QC	Boy	64	17294.203	2378.785	297.348
	Girl	57	15776.649	1974.431	261.520
rh_lateralorbitofrontal_Vol_no_QC	Boy	64	9575.719	1150.921	143.865
	Girl	57	8978.544	1052.187	139.366
rh_lingual_Vol_no_QC	Boy	64	9208.656	1416.197	177.025
	Girl	57	8795.702	1353.070	179.218
rh_medialorbitofrontal_Vol_no_QC	Boy	64	7228.766	808.221	101.028
	Girl	57	6685.912	780.949	103.439
rh_middletemporal_Vol_no_QC	Boy	64	15201.078	2056.709	257.089
	Girl	57	14445.228	1808.972	239.604
rh_parahippocampal_Vol_no_QC	Boy	64	2086.375	316.832	39.604
	Girl	57	2102.895	346.812	45.936
rh_paracentral_Vol_no_QC	Boy	64	5268.063	599.376	74.922
	Girl	57	4983.035	706.532	93.582
rh_parsopercularis_Vol_no_QC	Boy	64	5269.188	772.336	96.542
	Girl	57	5139.632	757.827	100.377
rh_parsorbitalis_Vol_no_QC	Boy	64	4236.609	512.572	64.072
	Girl	57	3951.263	456.550	60.471
rh_parstriangularis_Vol_no_QC	Boy	64	6361.734	1131.285	141.411
	Girl	57	5870.018	769.451	101.916
rh_pericalcarine_Vol_no_QC	Boy	64	2817.766	472.017	59.002
	Girl	57	2731.772	593.283	78.582
rh_postcentral_Vol_no_QC	Boy	64	12255.203	1385.266	173.158
	Girl	57	11750.789	1686.915	223.437
rh_posteriorcingulate_Vol_no_QC	Boy	64	4380.813	617.182	77.148
	Girl	57	4127.772	589.591	78.093
rh_precentral_Vol_no_QC	Boy	64	16119.125	1434.151	179.269
	Girl	57	15025.070	1645.351	217.932
rh_precuneus_Vol_no_QC	Boy	64	15023.063	1784.029	223.004
	Girl	57	13973.228	1731.498	229.343
rh_rostralanteriorcingulate_Vol_no_QC	Boy	64	2301.938	515.526	64.441
	Girl	57	2181.053	457.536	60.602
rh_rostralmiddlefrontal_Vol_no_QC	Boy	64	24707.609	3021.246	377.656
	Girl	57	22247.947	2729.465	361.526
rh_superiorfrontal_Vol_no_QC	Boy	64	29500.219	2796.482	349.560
	Girl	57	27712.316	2734.779	362.230
rh_superiorparietal_Vol_no_QC	Boy	64	18852.750	2173.659	271.707
	Girl	57	18038.719	2506.315	331.969
rh_superiortemporal_Vol_no_QC	Boy	64	15216.063	1593.692	199.211
	Girl	57	15035.088	1477.418	195.689
rh_supramarginal_Vol_no_QC	Boy	64	14477.031	2091.173	261.397
	Girl	57	13449.035	2052.523	271.863
rh_frontalpole_Vol_no_QC	Boy	64	1788.797	287.797	35.975
	Girl	57	1776.000	252.202	33.405
rh_temporalpole_Vol_no_QC	Boy	64	2625.109	433.272	54.159
	Girl	57	2522.912	437.094	57.895
rh_transversetemporal_Vol_no_QC	Boy	64	1254.000	213.973	26.747
	Girl	57	1223.684	185.146	24.523
rh_insula_Vol_no_QC	Boy	64	7991.344	874.495	109.312
	Girl	57	7371.965	757.241	100.299

Structural Asymmetry - Cortical Thickness - Quality Control

Paired Samples T-Test

Measure 1		Measure 2	t	df	p	Mean Difference	SE Difference	95% CI for Mean Difference Lower	95% CI for Mean Difference Upper	Cohen's d
lh_bankssts_CT_QC	-	rh_bankssts_CT_QC	−3.071	87	0.003	−0.077	0.025	−0.127	−0.027	−0.327
lh_caudalanteriorcingulate_CT_QC	-	rh_caudalanteriorcingulate_CT_QC	3.941	84	< .001	0.119	0.030	0.059	0.179	0.427
lh_caudalmiddlefrontal_CT_QC	-	rh_caudalmiddlefrontal_CT_QC	−0.217	110	0.829	−0.003	0.014	−0.031	0.024	−0.021
lh_cuneus_CT_QC	-	rh_cuneus_CT_QC	−3.080	75	0.003	−0.045	0.015	−0.074	−0.016	−0.353
lh_entorhinal_CT_QC	-	rh_entorhinal_CT_QC	−7.179	119	< .001	−0.198	0.028	−0.252	−0.143	−0.655
lh_fusiform_CT_QC	-	rh_fusiform_CT_QC	−0.476	119	0.635	−0.004	0.009	−0.022	0.014	−0.043
lh_inferiorparietal_CT_QC	-	rh_inferiorparietal_CT_QC	0.797	88	0.427	0.008	0.010	−0.012	0.028	0.085
lh_inferiortemporal_CT_QC	-	rh_inferiortemporal_CT_QC	0.175	84	0.862	0.002	0.012	−0.022	0.027	0.019
lh_isthmuscingulate_CT_QC	-	rh_isthmuscingulate_CT_QC	0.071	119	0.944	0.001	0.017	−0.033	0.035	0.006
lh_lateraloccipital_CT_QC	-	rh_lateraloccipital_CT_QC	−10.412	97	< .001	−0.082	0.008	−0.097	−0.066	−1.052
lh_lateralorbitofrontal_CT_QC	-	rh_lateralorbitofrontal_CT_QC	4.034	112	< .001	0.059	0.015	0.030	0.088	0.380
lh_lingual_CT_QC	-	rh_lingual_CT_QC	−2.133	81	0.036	−0.027	0.013	−0.053	−0.002	−0.236
lh_medialorbitofrontal_CT_QC	-	rh_medialorbitofrontal_CT_QC	−5.749	73	< .001	−0.110	0.019	−0.148	−0.072	−0.668
lh_middletemporal_CT_QC	-	rh_middletemporal_CT_QC	−0.605	54	0.547	−0.009	0.015	−0.040	0.021	−0.082
lh_parahippocampal_CT_QC	-	rh_parahippocampal_CT_QC	5.677	119	< .001	0.130	0.023	0.084	0.175	0.518
lh_paracentral_CT_QC	-	rh_paracentral_CT_QC	−2.100	108	0.038	−0.025	0.012	−0.049	−0.001	−0.201
lh_parsopercularis_CT_QC	-	rh_parsopercularis_CT_QC	−0.435	115	0.664	−0.005	0.011	−0.028	0.018	−0.040
lh_parsorbitalis_CT_QC	-	rh_parsorbitalis_CT_QC	−0.049	118	0.961	−8.403e−4	0.017	−0.035	0.033	−0.004
lh_parstriangularis_CT_QC	-	rh_parstriangularis_CT_QC	−2.646	117	0.009	−0.034	0.013	−0.059	−0.009	−0.244
lh_pericalcarine_CT_QC	-	rh_pericalcarine_CT_QC	2.572	72	0.012	0.043	0.017	0.010	0.076	0.301
lh_postcentral_CT_QC	-	rh_postcentral_CT_QC	1.912	77	0.060	0.024	0.012	−9.714e−4	0.048	0.217
lh_posteriorcingulate_CT_QC	-	rh_posteriorcingulate_CT_QC	3.295	116	0.001	0.046	0.014	0.018	0.074	0.305
lh_precentral_CT_QC	-	rh_precentral_CT_QC	2.088	91	0.040	0.023	0.011	0.001	0.045	0.218
lh_precuneus_CT_QC	-	rh_precuneus_CT_QC	2.193	107	0.030	0.019	0.009	0.002	0.036	0.211
lh_rostralanteriorcingulate_CT_QC	-	rh_rostralanteriorcingulate_CT_QC	−1.418	103	0.159	−0.034	0.024	−0.081	0.013	−0.139
lh_rostralmiddlefrontal_CT_QC	-	rh_rostralmiddlefrontal_CT_QC	−1.338	113	0.184	−0.013	0.010	−0.032	0.006	−0.125
lh_superiorfrontal_CT_QC	-	rh_superiorfrontal_CT_QC	0.429	67	0.669	0.005	0.013	−0.020	0.030	0.052
lh_superiorparietal_CT_QC	-	rh_superiorparietal_CT_QC	0.001	76	0.999	1.299e−5	0.009	−0.018	0.018	1.601e−4
lh_superiortemporal_CT_QC	-	rh_superiortemporal_CT_QC	−5.345	57	< .001	−0.067	0.013	−0.092	−0.042	−0.702
lh_supramarginal_CT_QC	-	rh_supramarginal_CT_QC	0.260	63	0.795	0.004	0.014	−0.025	0.032	0.033
lh_frontalpole_CT_QC	-	rh_frontalpole_CT_QC	2.365	117	0.020	0.078	0.033	0.013	0.144	0.218
lh_temporalpole_CT_QC	-	rh_temporalpole_CT_QC	−3.636	114	< .001	−0.125	0.034	−0.192	−0.057	−0.339
lh_transversetemporal_CT_QC	-	rh_transversetemporal_CT_QC	−1.768	120	0.080	−0.043	0.024	−0.091	0.005	−0.161
lh_insula_CT_QC	-	rh_insula_CT_QC	−1.135	75	0.260	−0.018	0.016	−0.049	0.013	−0.130

Note. Student's t-test.

Descriptives

Descriptives

	N	Mean	SD	SE
lh_bankssts_CT_QC	95	2.993	0.204	0.021
rh_bankssts_CT_QC	110	3.060	0.193	0.018
lh_caudalanteriorcingulate_CT_QC	96	2.853	0.292	0.030
rh_caudalanteriorcingulate_CT_QC	106	2.756	0.249	0.024
lh_caudalmiddlefrontal_CT_QC	116	2.873	0.148	0.014
rh_caudalmiddlefrontal_CT_QC	114	2.885	0.164	0.015
lh_cuneus_CT_QC	93	2.277	0.152	0.016
rh_cuneus_CT_QC	92	2.326	0.155	0.016
lh_entorhinal_CT_QC	121	3.245	0.320	0.029
rh_entorhinal_CT_QC	120	3.440	0.355	0.032
lh_fusiform_CT_QC	120	3.069	0.113	0.010
rh_fusiform_CT_QC	121	3.071	0.109	0.010
lh_inferiorparietal_CT_QC	102	2.964	0.118	0.012
rh_inferiorparietal_CT_QC	102	2.957	0.141	0.014
lh_inferiortemporal_CT_QC	105	3.198	0.140	0.014
rh_inferiortemporal_CT_QC	94	3.188	0.143	0.015
lh_isthmuscingulate_CT_QC	120	2.692	0.188	0.017
rh_isthmuscingulate_CT_QC	121	2.690	0.181	0.016
lh_lateraloccipital_CT_QC	105	2.517	0.116	0.011
rh_lateraloccipital_CT_QC	111	2.595	0.124	0.012
lh_lateralorbitofrontal_CT_QC	113	3.091	0.159	0.015
rh_lateralorbitofrontal_CT_QC	119	3.032	0.161	0.015
lh_lingual_CT_QC	100	2.407	0.141	0.014
rh_lingual_CT_QC	93	2.436	0.135	0.014
lh_medialorbitofrontal_CT_QC	95	2.850	0.177	0.018
rh_medialorbitofrontal_CT_QC	92	2.941	0.171	0.018
lh_middletemporal_CT_QC	77	3.280	0.129	0.015
rh_middletemporal_CT_QC	74	3.308	0.137	0.016
lh_parahippocampal_CT_QC	120	3.033	0.285	0.026
rh_parahippocampal_CT_QC	121	2.899	0.239	0.022
lh_paracentral_CT_QC	116	2.854	0.141	0.013
rh_paracentral_CT_QC	113	2.870	0.133	0.013
lh_parsopercularis_CT_QC	120	2.994	0.129	0.012
rh_parsopercularis_CT_QC	117	3.003	0.129	0.012
lh_parsorbitalis_CT_QC	120	3.262	0.217	0.020
rh_parsorbitalis_CT_QC	120	3.260	0.226	0.021
lh_parstriangularis_CT_QC	120	3.003	0.159	0.015
rh_parstriangularis_CT_QC	119	3.037	0.154	0.014
lh_pericalcarine_CT_QC	90	1.878	0.156	0.016
rh_pericalcarine_CT_QC	87	1.838	0.139	0.015
lh_postcentral_CT_QC	95	2.423	0.132	0.014
rh_postcentral_CT_QC	91	2.396	0.134	0.014
lh_posteriorcingulate_CT_QC	119	2.827	0.153	0.014
rh_posteriorcingulate_CT_QC	119	2.785	0.156	0.014
lh_precentral_CT_QC	105	2.786	0.112	0.011
rh_precentral_CT_QC	99	2.753	0.123	0.012
lh_precuneus_CT_QC	110	2.902	0.120	0.011
rh_precuneus_CT_QC	118	2.886	0.107	0.010
lh_rostralanteriorcingulate_CT_QC	106	3.126	0.239	0.023
rh_rostralanteriorcingulate_CT_QC	119	3.148	0.245	0.022
lh_rostralmiddlefrontal_CT_QC	119	2.863	0.137	0.013
rh_rostralmiddlefrontal_CT_QC	116	2.880	0.124	0.012
lh_superiorfrontal_CT_QC	81	3.166	0.143	0.016
rh_superiorfrontal_CT_QC	97	3.163	0.132	0.013
lh_superiorparietal_CT_QC	93	2.637	0.125	0.013
rh_superiorparietal_CT_QC	88	2.632	0.114	0.012
lh_superiortemporal_CT_QC	77	3.227	0.138	0.016
rh_superiortemporal_CT_QC	83	3.310	0.151	0.017
lh_supramarginal_CT_QC	86	3.021	0.137	0.015
rh_supramarginal_CT_QC	88	3.029	0.125	0.013
lh_frontalpole_CT_QC	119	3.413	0.334	0.031
rh_frontalpole_CT_QC	120	3.325	0.309	0.028
lh_temporalpole_CT_QC	117	3.577	0.301	0.028
rh_temporalpole_CT_QC	118	3.704	0.346	0.032
lh_transversetemporal_CT_QC	121	2.855	0.260	0.024
rh_transversetemporal_CT_QC	121	2.898	0.261	0.024
lh_insula_CT_QC	100	3.243	0.154	0.015
rh_insula_CT_QC	93	3.253	0.166	0.017

Structural Asymmetry - Cortical Thickness - No Quality Control

Paired Samples T-Test

Measure 1		Measure 2	t	df	p	Mean Difference	SE Difference	95% CI for Mean Difference		Cohen's d
								Lower	Upper	
lh_bankssts_CT_no_QC	-	rh_bankssts_CT_no_QC	−4.296	120	< .001	−0.093	0.022	−0.137	−0.050	−0.391
lh_caudalanteriorcingulate_CT_no_QC	-	rh_caudalanteriorcingulate_CT_no_QC	4.475	120	< .001	0.113	0.025	0.063	0.162	0.407
lh_caudalmiddlefrontal_CT_no_QC	-	rh_caudalmiddlefrontal_CT_no_QC	1.076	120	0.284	0.016	0.014	−0.013	0.044	0.098
lh_cuneus_CT_no_QC	-	rh_cuneus_CT_no_QC	−3.942	120	< .001	−0.046	0.012	−0.069	−0.023	−0.358
lh_entorhinal_CT_no_QC	-	rh_entorhinal_CT_no_QC	−6.159	120	< .001	−0.169	0.027	−0.223	−0.115	−0.560
lh_fusiform_CT_no_QC	-	rh_fusiform_CT_no_QC	1.451	120	0.149	0.013	0.009	−0.005	0.032	0.132
lh_inferiorparietal_CT_no_QC	-	rh_inferiorparietal_CT_no_QC	2.237	120	0.027	0.022	0.010	0.003	0.042	0.203
lh_inferiortemporal_CT_no_QC	-	rh_inferiortemporal_CT_no_QC	2.648	120	0.009	0.035	0.013	0.009	0.061	0.241
lh_isthmuscingulate_CT_no_QC	-	rh_isthmuscingulate_CT_no_QC	−0.610	120	0.543	−0.011	0.018	−0.047	0.025	−0.055
lh_lateraloccipital_CT_no_QC	-	rh_lateraloccipital_CT_no_QC	−10.172	120	< .001	−0.079	0.008	−0.095	−0.064	−0.925
lh_lateralorbitofrontal_CT_no_QC	-	rh_lateralorbitofrontal_CT_no_QC	3.092	120	0.002	0.040	0.013	0.015	0.066	0.281
lh_lingual_CT_no_QC	-	rh_lingual_CT_no_QC	−2.396	120	0.018	−0.026	0.011	−0.048	−0.005	−0.218
lh_medialorbitofrontal_CT_no_QC	-	rh_medialorbitofrontal_CT_no_QC	−6.142	120	< .001	−0.101	0.016	−0.134	−0.069	−0.558
lh_middletemporal_CT_no_QC	-	rh_middletemporal_CT_no_QC	−2.780	120	0.006	−0.039	0.014	−0.067	−0.011	−0.253
lh_parahippocampal_CT_no_QC	-	rh_parahippocampal_CT_no_QC	5.634	120	< .001	0.127	0.022	0.082	0.171	0.512
lh_paracentral_CT_no_QC	-	rh_paracentral_CT_no_QC	−1.631	120	0.106	−0.020	0.012	−0.044	0.004	−0.148
lh_parsopercularis_CT_no_QC	-	rh_parsopercularis_CT_no_QC	−1.355	120	0.178	−0.015	0.011	−0.036	0.007	−0.123
lh_parsorbitalis_CT_no_QC	-	rh_parsorbitalis_CT_no_QC	−0.819	120	0.415	−0.015	0.019	−0.053	0.022	−0.074
lh_parstriangularis_CT_no_QC	-	rh_parstriangularis_CT_no_QC	−2.934	120	0.004	−0.037	0.013	−0.062	−0.012	−0.267
lh_pericalcarine_CT_no_QC	-	rh_pericalcarine_CT_no_QC	2.026	120	0.045	0.027	0.013	6.094e−4	0.052	0.184
lh_postcentral_CT_no_QC	-	rh_postcentral_CT_no_QC	1.608	120	0.110	0.017	0.011	−0.004	0.039	0.146
lh_posteriorcingulate_CT_no_QC	-	rh_posteriorcingulate_CT_no_QC	3.170	120	0.002	0.046	0.015	0.017	0.075	0.288
lh_precentral_CT_no_QC	-	rh_precentral_CT_no_QC	4.258	120	< .001	0.043	0.010	0.023	0.063	0.387
lh_precuneus_CT_no_QC	-	rh_precuneus_CT_no_QC	1.571	120	0.119	0.013	0.009	−0.004	0.030	0.143
lh_rostralanteriorcingulate_CT_no_QC	-	rh_rostralanteriorcingulate_CT_no_QC	−1.920	120	0.057	−0.048	0.025	−0.098	0.002	−0.175
lh_rostralmiddlefrontal_CT_no_QC	-	rh_rostralmiddlefrontal_CT_no_QC	−1.255	120	0.212	−0.012	0.010	−0.031	0.007	−0.114
lh_superiorfrontal_CT_no_QC	-	rh_superiorfrontal_CT_no_QC	1.652	120	0.101	0.014	0.009	−0.003	0.032	0.150
lh_superiorparietal_CT_no_QC	-	rh_superiorparietal_CT_no_QC	1.495	120	0.137	0.011	0.008	−0.004	0.026	0.136
lh_superiortemporal_CT_no_QC	-	rh_superiortemporal_CT_no_QC	−8.163	120	< .001	−0.088	0.011	−0.109	−0.066	−0.742
lh_supramarginal_CT_no_QC	-	rh_supramarginal_CT_no_QC	1.456	120	0.148	0.019	0.013	−0.007	0.045	0.132
lh_frontalpole_CT_no_QC	-	rh_frontalpole_CT_no_QC	1.780	120	0.078	0.063	0.035	−0.007	0.133	0.162
lh_temporalpole_CT_no_QC	-	rh_temporalpole_CT_no_QC	−3.845	120	< .001	−0.129	0.034	−0.196	−0.063	−0.350
lh_transversetemporal_CT_no_QC	-	rh_transversetemporal_CT_no_QC	−2.129	120	0.035	−0.056	0.026	−0.108	−0.004	−0.194
lh_insula_CT_no_QC	-	rh_insula_CT_no_QC	−0.748	120	0.456	−0.011	0.015	−0.040	0.018	−0.068

Note. Student's t-test.

Descriptives

Descriptives

	N	Mean	SD	SE
lh_bankssts_CT_no_QC	121	2.977	0.208	0.019
rh_bankssts_CT_no_QC	121	3.071	0.206	0.019
lh_caudalanteriorcingulate_CT_no_QC	121	2.896	0.285	0.026
rh_caudalanteriorcingulate_CT_no_QC	121	2.783	0.242	0.022
lh_caudalmiddlefrontal_CT_no_QC	121	2.832	0.171	0.016
rh_caudalmiddlefrontal_CT_no_QC	121	2.817	0.193	0.018
lh_cuneus_CT_no_QC	121	2.287	0.158	0.014
rh_cuneus_CT_no_QC	121	2.333	0.149	0.014
lh_entorhinal_CT_no_QC	121	3.259	0.311	0.028
rh_entorhinal_CT_no_QC	121	3.428	0.337	0.031
lh_fusiform_CT_no_QC	121	3.074	0.108	0.010
rh_fusiform_CT_no_QC	121	3.061	0.112	0.010
lh_inferiorparietal_CT_no_QC	121	2.953	0.136	0.012
rh_inferiorparietal_CT_no_QC	121	2.931	0.145	0.013
lh_inferiortemporal_CT_no_QC	121	3.157	0.151	0.014
rh_inferiortemporal_CT_no_QC	121	3.122	0.164	0.015
lh_isthmuscingulate_CT_no_QC	121	2.695	0.185	0.017
rh_isthmuscingulate_CT_no_QC	121	2.706	0.198	0.018
lh_lateraloccipital_CT_no_QC	121	2.505	0.121	0.011
rh_lateraloccipital_CT_no_QC	121	2.584	0.130	0.012
lh_lateralorbitofrontal_CT_no_QC	121	3.084	0.152	0.014
rh_lateralorbitofrontal_CT_no_QC	121	3.044	0.161	0.015
lh_lingual_CT_no_QC	121	2.414	0.147	0.013
rh_lingual_CT_no_QC	121	2.440	0.144	0.013
lh_medialorbitofrontal_CT_no_QC	121	2.854	0.195	0.018
rh_medialorbitofrontal_CT_no_QC	121	2.955	0.171	0.016
lh_middletemporal_CT_no_QC	121	3.249	0.160	0.015
rh_middletemporal_CT_no_QC	121	3.288	0.156	0.014
lh_parahippocampal_CT_no_QC	121	3.022	0.284	0.026
rh_parahippocampal_CT_no_QC	121	2.895	0.246	0.022
lh_paracentral_CT_no_QC	121	2.842	0.150	0.014
rh_paracentral_CT_no_QC	121	2.862	0.135	0.012
lh_parsopercularis_CT_no_QC	121	2.999	0.123	0.011
rh_parsopercularis_CT_no_QC	121	3.013	0.125	0.011
lh_parsorbitalis_CT_no_QC	121	3.252	0.238	0.022
rh_parsorbitalis_CT_no_QC	121	3.267	0.239	0.022
lh_parstriangularis_CT_no_QC	121	3.006	0.167	0.015
rh_parstriangularis_CT_no_QC	121	3.043	0.152	0.014
lh_pericalcarine_CT_no_QC	121	1.878	0.152	0.014
rh_pericalcarine_CT_no_QC	121	1.851	0.143	0.013
lh_postcentral_CT_no_QC	121	2.404	0.126	0.011
rh_postcentral_CT_no_QC	121	2.386	0.125	0.011
lh_posteriorcingulate_CT_no_QC	121	2.836	0.161	0.015
rh_posteriorcingulate_CT_no_QC	121	2.790	0.155	0.014
lh_precentral_CT_no_QC	121	2.760	0.134	0.012
rh_precentral_CT_no_QC	121	2.717	0.130	0.012
lh_precuneus_CT_no_QC	121	2.903	0.118	0.011
rh_precuneus_CT_no_QC	121	2.890	0.104	0.009
lh_rostralanteriorcingulate_CT_no_QC	121	3.144	0.250	0.023
rh_rostralanteriorcingulate_CT_no_QC	121	3.192	0.231	0.021
lh_rostralmiddlefrontal_CT_no_QC	121	2.863	0.148	0.013
rh_rostralmiddlefrontal_CT_no_QC	121	2.875	0.134	0.012
lh_superiorfrontal_CT_no_QC	121	3.126	0.154	0.014
rh_superiorfrontal_CT_no_QC	121	3.111	0.164	0.015
lh_superiorparietal_CT_no_QC	121	2.583	0.135	0.012
rh_superiorparietal_CT_no_QC	121	2.572	0.129	0.012
lh_superiortemporal_CT_no_QC	121	3.217	0.155	0.014
rh_superiortemporal_CT_no_QC	121	3.305	0.149	0.014
lh_supramarginal_CT_no_QC	121	3.009	0.147	0.013
rh_supramarginal_CT_no_QC	121	2.990	0.141	0.013
lh_frontalpole_CT_no_QC	121	3.389	0.363	0.033
rh_frontalpole_CT_no_QC	121	3.326	0.317	0.029
lh_temporalpole_CT_no_QC	121	3.584	0.314	0.029
rh_temporalpole_CT_no_QC	121	3.713	0.334	0.030
lh_transversetemporal_CT_no_QC	121	2.852	0.257	0.023
rh_transversetemporal_CT_no_QC	121	2.908	0.272	0.025
lh_insula_CT_no_QC	121	3.224	0.161	0.015
rh_insula_CT_no_QC	121	3.234	0.163	0.015

Structural Asymmetry - Surface Area - Quality Controlled

Paired Samples T-Test

Measure 1		Measure 2	t	df	p	Mean Difference	SE Difference	95% CI for Mean Difference Lower	95% CI for Mean Difference Upper	Cohen's d
lh_bankssts_SA_QC	-	rh_bankssts_SA_QC	7.773	87	< .001	131.250	16.885	97.689	164.811	0.829
lh_caudalanteriorcingulate_SA_QC	-	rh_caudalanteriorcingulate_SA_QC	−6.729	84	< .001	−113.247	16.829	−146.714	−79.780	−0.730
lh_caudalmiddlefrontal_SA_QC	-	rh_caudalmiddlefrontal_SA_QC	3.720	110	< .001	127.027	34.149	59.352	194.702	0.353
lh_cuneus_SA_QC	-	rh_cuneus_SA_QC	−4.505	75	< .001	−94.513	20.978	−136.303	−52.723	−0.517
lh_entorhinal_SA_QC	-	rh_entorhinal_SA_QC	7.634	119	< .001	55.750	7.302	41.290	70.210	0.697
lh_fusiform_SA_QC	-	rh_fusiform_SA_QC	4.350	119	< .001	127.283	29.258	69.349	185.218	0.397
lh_inferiorparietal_SA_QC	-	rh_inferiorparietal_SA_QC	−12.207	88	< .001	−987.472	80.891	−1148.226	−826.717	−1.294
lh_inferiortemporal_SA_QC	-	rh_inferiortemporal_SA_QC	1.587	84	0.116	70.106	44.164	−17.719	157.931	0.172
lh_isthmuscingulate_SA_QC	-	rh_isthmuscingulate_SA_QC	6.356	119	< .001	84.125	13.236	57.917	110.333	0.580
lh_lateraloccipital_SA_QC	-	rh_lateraloccipital_SA_QC	−0.981	97	0.329	−52.888	53.908	−159.881	54.105	−0.099
lh_lateralorbitofrontal_SA_QC	-	rh_lateralorbitofrontal_SA_QC	−0.184	112	0.854	−4.752	25.810	−55.891	46.387	−0.017
lh_lingual_SA_QC	-	rh_lingual_SA_QC	−5.092	81	< .001	−156.220	30.680	−217.264	−95.175	−0.562
lh_medialorbitofrontal_SA_QC	-	rh_medialorbitofrontal_SA_QC	−1.678	73	0.098	−39.689	23.657	−86.837	7.458	−0.195
lh_middletemporal_SA_QC	-	rh_middletemporal_SA_QC	−8.776	54	< .001	−348.655	39.729	−428.306	−269.003	−1.183
lh_parahippocampal_SA_QC	-	rh_parahippocampal_SA_QC	4.407	119	< .001	28.425	6.449	15.655	41.195	0.402
lh_paracentral_SA_QC	-	rh_paracentral_SA_QC	−6.671	108	< .001	−128.367	19.242	−166.507	−90.227	−0.639
lh_parsopercularis_SA_QC	-	rh_parsopercularis_SA_QC	10.256	115	< .001	224.595	21.900	181.216	267.974	0.952
lh_parsorbitalis_SA_QC	-	rh_parsorbitalis_SA_QC	−20.415	118	< .001	−174.731	8.559	−191.680	−157.782	−1.871
lh_parstriangularis_SA_QC	-	rh_parstriangularis_SA_QC	−11.581	117	< .001	−250.364	21.619	−293.180	−207.549	−1.066
lh_pericalcarine_SA_QC	-	rh_pericalcarine_SA_QC	−11.519	72	< .001	−156.438	13.581	−183.512	−129.365	−1.348
lh_postcentral_SA_QC	-	rh_postcentral_SA_QC	1.672	77	0.099	68.795	41.143	−13.132	150.721	0.189
lh_posteriorcingulate_SA_QC	-	rh_posteriorcingulate_SA_QC	−2.684	116	0.008	−48.410	18.038	−84.136	−12.684	−0.248
lh_precentral_SA_QC	-	rh_precentral_SA_QC	−2.217	91	0.029	−86.011	38.789	−163.060	−8.961	−0.231
lh_precuneus_SA_QC	-	rh_precuneus_SA_QC	−4.285	107	< .001	−140.537	32.800	−205.560	−75.514	−0.412
lh_rostralanteriorcingulate_SA_QC	-	rh_rostralanteriorcingulate_SA_QC	14.038	103	< .001	184.798	13.164	158.690	210.906	1.377
lh_rostralmiddlefrontal_SA_QC	-	rh_rostralmiddlefrontal_SA_QC	−5.050	113	< .001	−264.588	52.397	−368.395	−160.781	−0.473
lh_superiorfrontal_SA_QC	-	rh_superiorfrontal_SA_QC	5.053	67	< .001	374.632	74.145	226.639	522.626	0.613
lh_superiorparietal_SA_QC	-	rh_superiorparietal_SA_QC	2.154	76	0.034	137.364	63.770	10.355	264.372	0.245
lh_superiortemporal_SA_QC	-	rh_superiortemporal_SA_QC	6.446	57	< .001	266.379	41.322	183.634	349.125	0.846
lh_supramarginal_SA_QC	-	rh_supramarginal_SA_QC	6.424	63	< .001	449.734	70.010	309.830	589.639	0.803
lh_frontalpole_SA_QC	-	rh_frontalpole_SA_QC	−16.824	117	< .001	−70.203	4.173	−78.468	−61.939	−1.549
lh_temporalpole_SA_QC	-	rh_temporalpole_SA_QC	0.719	114	0.473	3.922	5.452	−6.878	14.721	0.067
lh_transversetemporal_SA_QC	-	rh_transversetemporal_SA_QC	17.652	120	< .001	108.860	6.167	96.649	121.070	1.605
lh_insula_SA_QC	-	rh_insula_SA_QC	5.607	75	< .001	97.855	17.452	63.090	132.621	0.643

Note. Student's t-test.

Descriptives

Descriptives

	N	Mean	SD	SE
lh_bankssts_SA_QC	95	1040.653	184.321	18.911
rh_bankssts_SA_QC	110	924.836	137.128	13.075
lh_caudalanteriorcingulate_SA_QC	96	593.427	104.292	10.644
rh_caudalanteriorcingulate_SA_QC	106	702.047	138.821	13.483
lh_caudalmiddlefrontal_SA_QC	116	2513.940	372.975	34.630
rh_caudalmiddlefrontal_SA_QC	114	2375.474	379.252	35.520
lh_cuneus_SA_QC	93	1645.258	221.660	22.985
rh_cuneus_SA_QC	92	1704.924	208.742	21.763
lh_entorhinal_SA_QC	121	464.669	95.069	8.643
rh_entorhinal_SA_QC	120	407.600	86.857	7.929
lh_fusiform_SA_QC	120	3301.917	382.642	34.930
rh_fusiform_SA_QC	121	3169.504	389.077	35.371
lh_inferiorparietal_SA_QC	102	5028.294	745.080	73.774
rh_inferiorparietal_SA_QC	102	6045.804	906.890	89.796
lh_inferiortemporal_SA_QC	105	3508.190	473.622	46.221
rh_inferiortemporal_SA_QC	94	3390.170	499.982	51.569
lh_isthmuscingulate_SA_QC	120	1098.058	157.245	14.354
rh_isthmuscingulate_SA_QC	121	1014.570	163.183	14.835
lh_lateraloccipital_SA_QC	105	5538.324	732.438	71.479
rh_lateraloccipital_SA_QC	111	5593.351	731.680	69.448
lh_lateralorbitofrontal_SA_QC	113	2711.965	295.095	27.760
rh_lateralorbitofrontal_SA_QC	119	2718.697	415.541	38.093
lh_lingual_SA_QC	100	3117.500	451.606	45.161
rh_lingual_SA_QC	93	3217.516	421.526	43.710
lh_medialorbitofrontal_SA_QC	95	1921.137	237.527	24.370
rh_medialorbitofrontal_SA_QC	92	1990.891	268.358	27.978
lh_middletemporal_SA_QC	77	3246.558	391.704	44.639
rh_middletemporal_SA_QC	74	3577.824	422.501	49.115
lh_parahippocampal_SA_QC	120	649.167	84.547	7.718
rh_parahippocampal_SA_QC	121	620.802	76.927	6.993
lh_paracentral_SA_QC	116	1474.621	177.431	16.474
rh_paracentral_SA_QC	113	1610.177	197.209	18.552
lh_parsopercularis_SA_QC	120	1698.958	259.990	23.734
rh_parsopercularis_SA_QC	117	1466.598	215.952	19.965
lh_parsorbitalis_SA_QC	120	741.375	94.828	8.657
rh_parsorbitalis_SA_QC	120	917.142	115.816	10.572
lh_parstriangularis_SA_QC	120	1428.692	228.398	20.850
rh_parstriangularis_SA_QC	119	1681.059	288.718	26.467
lh_pericalcarine_SA_QC	90	1489.311	222.108	23.412
rh_pericalcarine_SA_QC	87	1611.023	203.593	21.827
lh_postcentral_SA_QC	95	4526.684	427.966	43.908
rh_postcentral_SA_QC	91	4454.088	545.917	57.228
lh_posteriorcingulate_SA_QC	119	1269.824	183.402	16.812
rh_posteriorcingulate_SA_QC	119	1316.983	186.934	17.136
lh_precentral_SA_QC	105	5103.057	452.466	44.156
rh_precentral_SA_QC	99	5185.869	481.733	48.416
lh_precuneus_SA_QC	110	4346.382	563.497	53.727
rh_precuneus_SA_QC	118	4494.475	581.576	53.538
lh_rostralanteriorcingulate_SA_QC	106	781.613	152.047	14.768
rh_rostralanteriorcingulate_SA_QC	119	598.815	122.845	11.261
lh_rostralmiddlefrontal_SA_QC	119	6391.319	852.545	78.153
rh_rostralmiddlefrontal_SA_QC	116	6634.526	900.276	83.589
lh_superiorfrontal_SA_QC	81	7844.148	933.274	103.697
rh_superiorfrontal_SA_QC	97	7523.732	921.679	93.582
lh_superiorparietal_SA_QC	93	6345.720	945.691	98.064
rh_superiorparietal_SA_QC	88	6161.716	781.443	83.302
lh_superiortemporal_SA_QC	77	4044.429	422.607	48.161
rh_superiortemporal_SA_QC	83	3811.301	404.004	44.345
lh_supramarginal_SA_QC	86	4393.581	808.014	87.130
rh_supramarginal_SA_QC	88	3950.273	593.321	63.248
lh_frontalpole_SA_QC	119	270.210	29.890	2.740
rh_frontalpole_SA_QC	120	339.717	40.700	3.715
lh_temporalpole_SA_QC	117	456.547	55.055	5.090
rh_temporalpole_SA_QC	118	453.144	60.234	5.545
lh_transversetemporal_SA_QC	121	474.496	75.912	6.901
rh_transversetemporal_SA_QC	121	365.636	51.571	4.688
lh_insula_SA_QC	100	2379.300	237.608	23.761
rh_insula_SA_QC	93	2297.505	261.140	27.079

Structural Asymmetry - Surface Area - No Quality Control

Paired Samples T-Test

Measure 1		Measure 2	t	df	p	Mean Difference	SE Difference	95% CI for Mean Difference Lower	Upper	Cohen's d
lh_bankssts_SA_no_QC	-	rh_bankssts_SA_no_QC	9.247	120	< .001	172.785	18.685	135.789	209.781	0.841
lh_caudalanteriorcingulate_SA_no_QC	-	rh_caudalanteriorcingulate_SA_no_QC	−6.667	120	< .001	−105.124	15.769	−136.345	−73.903	−0.606
lh_caudalmiddlefrontal_SA_no_QC	-	rh_caudalmiddlefrontal_SA_no_QC	2.898	120	0.004	106.645	36.798	33.786	179.503	0.263
lh_cuneus_SA_no_QC	-	rh_cuneus_SA_no_QC	−6.779	120	< .001	−119.719	17.659	−154.683	−84.755	−0.616
lh_entorhinal_SA_no_QC	-	rh_entorhinal_SA_no_QC	7.294	120	< .001	56.727	7.777	41.329	72.126	0.663
lh_fusiform_SA_no_QC	-	rh_fusiform_SA_no_QC	4.041	120	< .001	119.744	29.634	61.071	178.417	0.367
lh_inferiorparietal_SA_no_QC	-	rh_inferiorparietal_SA_no_QC	−15.213	120	< .001	−1023.537	67.282	−1156.750	−890.324	−1.383
lh_inferiortemporal_SA_no_QC	-	rh_inferiortemporal_SA_no_QC	1.257	120	0.211	42.851	34.082	−24.629	110.331	0.114
lh_isthmuscingulate_SA_no_QC	-	rh_isthmuscingulate_SA_no_QC	5.977	120	< .001	80.719	13.506	53.979	107.459	0.543
lh_lateraloccipital_SA_no_QC	-	rh_lateraloccipital_SA_no_QC	−1.019	120	0.310	−48.736	47.804	−143.383	45.912	−0.093
lh_lateralorbitofrontal_SA_no_QC	-	rh_lateralorbitofrontal_SA_no_QC	−0.601	120	0.549	−13.983	23.285	−60.085	32.118	−0.055
lh_lingual_SA_no_QC	-	rh_lingual_SA_no_QC	−5.358	120	< .001	−156.926	29.287	−214.911	−98.940	−0.487
lh_medialorbitofrontal_SA_no_QC	-	rh_medialorbitofrontal_SA_no_QC	−3.368	120	0.001	−71.752	21.305	−113.935	−29.569	−0.306
lh_middletemporal_SA_no_QC	-	rh_middletemporal_SA_no_QC	−11.354	120	< .001	−349.835	30.811	−410.839	−288.831	−1.032
lh_parahippocampal_SA_no_QC	-	rh_parahippocampal_SA_no_QC	4.305	120	< .001	31.934	7.418	17.247	46.621	0.391
lh_paracentral_SA_no_QC	-	rh_paracentral_SA_no_QC	−7.327	120	< .001	−137.810	18.808	−175.049	−100.571	−0.666
lh_parsopercularis_SA_no_QC	-	rh_parsopercularis_SA_no_QC	9.455	120	< .001	223.215	23.607	176.475	269.955	0.860
lh_parsorbitalis_SA_no_QC	-	rh_parsorbitalis_SA_no_QC	−20.556	120	< .001	−172.843	8.409	−189.491	−156.195	−1.869
lh_parstriangularis_SA_no_QC	-	rh_parstriangularis_SA_no_QC	−11.668	120	< .001	−249.132	21.351	−291.406	−206.858	−1.061
lh_pericalcarine_SA_no_QC	-	rh_pericalcarine_SA_no_QC	−12.073	120	< .001	−171.529	14.208	−199.659	−143.399	−1.098
lh_postcentral_SA_no_QC	-	rh_postcentral_SA_no_QC	2.016	120	0.046	74.942	37.180	1.327	148.557	0.183
lh_posteriorcingulate_SA_no_QC	-	rh_posteriorcingulate_SA_no_QC	−2.462	120	0.015	−45.364	18.424	−81.842	−8.886	−0.224
lh_precentral_SA_no_QC	-	rh_precentral_SA_no_QC	−2.840	120	0.005	−116.545	41.041	−197.804	−35.287	−0.258
lh_precuneus_SA_no_QC	-	rh_precuneus_SA_no_QC	−4.495	120	< .001	−146.430	32.577	−210.930	−81.929	−0.409
lh_rostralanteriorcingulate_SA_no_QC	-	rh_rostralanteriorcingulate_SA_no_QC	14.397	120	< .001	197.421	13.712	170.272	224.571	1.309
lh_rostralmiddlefrontal_SA_no_QC	-	rh_rostralmiddlefrontal_SA_no_QC	−4.476	120	< .001	−263.215	58.809	−379.653	−146.777	−0.407
lh_superiorfrontal_SA_no_QC	-	rh_superiorfrontal_SA_no_QC	5.739	120	< .001	296.140	51.605	193.967	398.314	0.522
lh_superiorparietal_SA_no_QC	-	rh_superiorparietal_SA_no_QC	1.462	120	0.146	85.140	58.230	−30.150	200.431	0.133
lh_superiortemporal_SA_no_QC	-	rh_superiortemporal_SA_no_QC	8.799	120	< .001	251.612	28.596	194.993	308.230	0.800
lh_supramarginal_SA_no_QC	-	rh_supramarginal_SA_no_QC	6.918	120	< .001	389.248	56.269	277.839	500.657	0.629
lh_frontalpole_SA_no_QC	-	rh_frontalpole_SA_no_QC	−16.425	120	< .001	−68.975	4.199	−77.290	−60.661	−1.493
lh_temporalpole_SA_no_QC	-	rh_temporalpole_SA_no_QC	2.349	120	0.020	12.000	5.109	1.884	22.116	0.214
lh_transversetemporal_SA_no_QC	-	rh_transversetemporal_SA_no_QC	16.983	120	< .001	103.975	6.122	91.853	116.097	1.544
lh_insula_SA_no_QC	-	rh_insula_SA_no_QC	3.671	120	< .001	76.388	20.811	35.184	117.593	0.334

Note. Student's t-test.

Descriptives

Descriptives

	N	Mean	SD	SE
lh_bankssts_SA_no_QC	121	1090.785	224.386	20.399
rh_bankssts_SA_no_QC	121	918.000	133.618	12.147
lh_caudalanteriorcingulate_SA_no_QC	121	615.248	123.294	11.209
rh_caudalanteriorcingulate_SA_no_QC	121	720.372	159.492	14.499
lh_caudalmiddlefrontal_SA_no_QC	121	2545.752	431.874	39.261
rh_caudalmiddlefrontal_SA_no_QC	121	2439.107	446.558	40.596
lh_cuneus_SA_no_QC	121	1644.223	223.717	20.338
rh_cuneus_SA_no_QC	121	1763.942	261.897	23.809
lh_entorhinal_SA_no_QC	121	459.190	96.908	8.810
rh_entorhinal_SA_no_QC	121	402.463	81.571	7.416
lh_fusiform_SA_no_QC	121	3301.636	384.566	34.961
rh_fusiform_SA_no_QC	121	3181.893	407.495	37.045
lh_inferiorparietal_SA_no_QC	121	5113.570	768.114	69.829
rh_inferiorparietal_SA_no_QC	121	6137.107	864.595	78.600
lh_inferiortemporal_SA_no_QC	121	3511.868	449.511	40.865
rh_inferiortemporal_SA_no_QC	121	3469.017	490.308	44.573
lh_isthmuscingulate_SA_no_QC	121	1100.736	153.629	13.966
rh_isthmuscingulate_SA_no_QC	121	1020.017	174.221	15.838
lh_lateraloccipital_SA_no_QC	121	5599.620	723.154	65.741
rh_lateraloccipital_SA_no_QC	121	5648.355	760.968	69.179
lh_lateralorbitofrontal_SA_no_QC	121	2705.033	307.466	27.951
rh_lateralorbitofrontal_SA_no_QC	121	2719.017	384.872	34.988
lh_lingual_SA_no_QC	121	3162.686	426.760	38.796
rh_lingual_SA_no_QC	121	3319.612	475.649	43.241
lh_medialorbitofrontal_SA_no_QC	121	1896.140	272.900	24.809
rh_medialorbitofrontal_SA_no_QC	121	1967.893	267.124	24.284
lh_middletemporal_SA_no_QC	121	3273.421	382.479	34.771
rh_middletemporal_SA_no_QC	121	3623.256	447.658	40.696
lh_parahippocampal_SA_no_QC	121	658.884	88.436	8.040
rh_parahippocampal_SA_no_QC	121	626.950	83.890	7.626
lh_paracentral_SA_no_QC	121	1490.810	202.434	18.403
rh_paracentral_SA_no_QC	121	1628.620	208.687	18.972
lh_parsopercularis_SA_no_QC	121	1711.678	284.621	25.875
rh_parsopercularis_SA_no_QC	121	1488.463	230.033	20.912
lh_parsorbitalis_SA_no_QC	121	744.570	95.284	8.662
rh_parsorbitalis_SA_no_QC	121	917.413	118.589	10.781
lh_parstriangularis_SA_no_QC	121	1431.711	227.398	20.673
rh_parstriangularis_SA_no_QC	121	1680.843	288.823	26.257
lh_pericalcarine_SA_no_QC	121	1521.934	249.450	22.677
rh_pericalcarine_SA_no_QC	121	1693.463	267.247	24.295
lh_postcentral_SA_no_QC	121	4574.554	460.622	41.875
rh_postcentral_SA_no_QC	121	4499.612	559.588	50.872
lh_posteriorcingulate_SA_no_QC	121	1272.008	185.532	16.867
rh_posteriorcingulate_SA_no_QC	121	1317.372	193.229	17.566
lh_precentral_SA_no_QC	121	5158.190	490.349	44.577
rh_precentral_SA_no_QC	121	5274.736	566.995	51.545
lh_precuneus_SA_no_QC	121	4383.769	591.085	53.735
rh_precuneus_SA_no_QC	121	4530.198	586.021	53.275
lh_rostralanteriorcingulate_SA_no_QC	121	790.711	170.494	15.499
rh_rostralanteriorcingulate_SA_no_QC	121	593.289	122.450	11.132
lh_rostralmiddlefrontal_SA_no_QC	121	6438.066	874.818	79.529
rh_rostralmiddlefrontal_SA_no_QC	121	6701.281	959.219	87.202
lh_superiorfrontal_SA_no_QC	121	7960.140	966.893	87.899
rh_superiorfrontal_SA_no_QC	121	7664.000	956.619	86.965
lh_superiorparietal_SA_no_QC	121	6501.579	988.743	89.886
rh_superiorparietal_SA_no_QC	121	6416.438	833.080	75.735
lh_superiortemporal_SA_no_QC	121	4132.421	456.724	41.520
rh_superiortemporal_SA_no_QC	121	3880.810	412.230	37.475
lh_supramarginal_SA_no_QC	121	4494.207	811.770	73.797
rh_supramarginal_SA_no_QC	121	4104.959	630.779	57.344
lh_frontalpole_SA_no_QC	121	269.587	27.829	2.530
rh_frontalpole_SA_no_QC	121	338.562	44.656	4.060
lh_temporalpole_SA_no_QC	121	458.926	60.963	5.542
rh_temporalpole_SA_no_QC	121	446.926	62.836	5.712
lh_transversetemporal_SA_no_QC	121	468.909	74.876	6.807
rh_transversetemporal_SA_no_QC	121	364.934	53.638	4.876
lh_insula_SA_no_QC	121	2455.289	272.439	24.767
rh_insula_SA_no_QC	121	2378.901	279.637	25.422

Structural Asymmetry - Volume - Quality Controlled

Paired Samples T-Test

								95% CI for Mean Difference		
Measure 1		Measure 2	t	df	p	Mean Difference	SE Difference	Lower	Upper	Cohen's d
lh_bankssts_Vol_QC	-	rh_bankssts_Vol_QC	6.105	87	< .001	326.477	53.474	220.192	432.763	0.651
lh_caudalanteriorcingulate_Vol_QC	-	rh_caudalanteriorcingulate_Vol_QC	−6.106	84	< .001	−435.776	71.364	−577.691	−293.862	−0.662
lh_caudalmiddlefrontal_Vol_QC	-	rh_caudalmiddlefrontal_Vol_QC	3.408	110	< .001	429.856	126.149	179.859	679.853	0.323
lh_cuneus_Vol_QC	-	rh_cuneus_Vol_QC	−9.450	75	< .001	−466.921	49.411	−565.353	−368.490	−1.084
lh_entorhinal_Vol_QC	-	rh_entorhinal_Vol_QC	1.646	119	0.102	57.867	35.153	−11.740	127.473	0.150
lh_fusiform_Vol_QC	-	rh_fusiform_Vol_QC	3.334	119	0.001	415.217	124.545	168.605	661.828	0.304
lh_inferiorparietal_Vol_QC	-	rh_inferiorparietal_Vol_QC	−13.689	88	< .001	−3787.955	276.707	−4337.852	−3238.058	−1.451
lh_inferiortemporal_Vol_QC	-	rh_inferiortemporal_Vol_QC	1.943	84	0.055	366.153	188.469	−8.638	740.944	0.211
lh_isthmuscingulate_Vol_QC	-	rh_isthmuscingulate_Vol_QC	2.661	119	0.009	122.592	46.068	31.373	213.810	0.243
lh_lateraloccipital_Vol_QC	-	rh_lateraloccipital_Vol_QC	−5.230	97	< .001	−871.510	166.635	−1202.235	−540.786	−0.528
lh_lateralorbitofrontal_Vol_QC	-	rh_lateralorbitofrontal_Vol_QC	5.128	112	< .001	305.009	59.480	187.156	422.861	0.482
lh_lingual_Vol_QC	-	rh_lingual_Vol_QC	−7.228	81	< .001	−682.463	94.419	−870.328	−494.599	−0.798
lh_medialorbitofrontal_Vol_QC	-	rh_medialorbitofrontal_Vol_QC	−4.982	73	< .001	−435.284	87.371	−609.413	−261.154	−0.579
lh_middletemporal_Vol_QC	-	rh_middletemporal_Vol_QC	−4.747	54	< .001	−956.255	201.424	−1360.086	−552.423	−0.640
lh_parahippocampal_Vol_QC	-	rh_parahippocampal_Vol_QC	8.120	119	< .001	197.392	24.308	149.258	245.525	0.741
lh_paracentral_Vol_QC	-	rh_paracentral_Vol_QC	−6.346	108	< .001	−417.028	65.717	−547.291	−286.764	−0.608
lh_parsopercularis_Vol_QC	-	rh_parsopercularis_Vol_QC	11.980	115	< .001	952.172	79.478	794.742	1109.603	1.112
lh_parsorbitalis_Vol_QC	-	rh_parsorbitalis_Vol_QC	−13.991	118	< .001	−624.983	44.669	−713.441	−536.526	−1.283
lh_parstriangularis_Vol_QC	-	rh_parstriangularis_Vol_QC	−10.621	117	< .001	−901.924	84.916	−1070.095	−733.752	−0.978
lh_pericalcarine_Vol_QC	-	rh_pericalcarine_Vol_QC	−7.709	72	< .001	−224.192	29.080	−282.162	−166.221	−0.902
lh_postcentral_Vol_QC	-	rh_postcentral_Vol_QC	4.212	77	< .001	547.064	129.868	288.465	805.663	0.477
lh_posteriorcingulate_Vol_QC	-	rh_posteriorcingulate_Vol_QC	−2.518	116	0.013	−157.538	62.555	−281.437	−33.640	−0.233
lh_precentral_Vol_QC	-	rh_precentral_Vol_QC	0.750	91	0.455	105.272	140.388	−173.592	384.136	0.078
lh_precuneus_Vol_QC	-	rh_precuneus_Vol_QC	−2.873	107	0.005	−297.389	103.499	−502.563	−92.215	−0.276
lh_rostralanteriorcingulate_Vol_QC	-	rh_rostralanteriorcingulate_Vol_QC	11.357	103	< .001	601.875	52.996	496.769	706.981	1.114
lh_rostralmiddlefrontal_Vol_QC	-	rh_rostralmiddlefrontal_Vol_QC	−4.590	113	< .001	−822.325	179.172	−1177.296	−467.353	−0.430
lh_superiorfrontal_Vol_QC	-	rh_superiorfrontal_Vol_QC	5.338	67	< .001	1506.588	282.251	943.214	2069.963	0.647
lh_superiorparietal_Vol_QC	-	rh_superiorparietal_Vol_QC	3.433	76	< .001	657.831	191.606	276.215	1039.447	0.391
lh_superiortemporal_Vol_QC	-	rh_superiortemporal_Vol_QC	5.753	57	< .001	1048.224	182.217	683.341	1413.107	0.755
lh_supramarginal_Vol_QC	-	rh_supramarginal_Vol_QC	6.689	63	< .001	1778.031	265.816	1246.841	2309.221	0.836
lh_frontalpole_Vol_QC	-	rh_frontalpole_Vol_QC	−10.608	117	< .001	−298.398	28.129	−354.106	−242.690	−0.977
lh_temporalpole_Vol_QC	-	rh_temporalpole_Vol_QC	−0.868	114	0.387	−36.304	41.826	−119.162	46.554	−0.081
lh_transversetemporal_Vol_QC	-	rh_transversetemporal_Vol_QC	12.920	120	< .001	282.157	21.840	238.916	325.398	1.175
lh_insula_Vol_QC	-	rh_insula_Vol_QC	5.049	75	< .001	268.671	53.218	162.656	374.686	0.579

Note. Student's t-test.

Descriptives

Descriptives

	N	Mean	SD	SE
lh_bankssts_Vol_QC	95	3040.600	586.608	60.185
rh_bankssts_Vol_QC	110	2744.764	428.804	40.885
lh_caudalanteriorcingulate_Vol_QC	96	1869.833	445.042	45.422
rh_caudalanteriorcingulate_Vol_QC	106	2312.802	545.530	52.987
lh_caudalmiddlefrontal_Vol_QC	116	8216.681	1241.725	115.291
rh_caudalmiddlefrontal_Vol_QC	114	7804.307	1256.986	117.728
lh_cuneus_Vol_QC	93	4054.688	601.524	62.375
rh_cuneus_Vol_QC	92	4457.587	683.019	71.210
lh_entorhinal_Vol_QC	121	2006.347	383.178	34.834
rh_entorhinal_Vol_QC	120	1939.208	405.677	37.033
lh_fusiform_Vol_QC	120	11882.567	1551.006	141.587
rh_fusiform_Vol_QC	121	11441.554	1500.120	136.375
lh_inferiorparietal_Vol_QC	102	17149.706	2437.889	241.387
rh_inferiorparietal_Vol_QC	102	21029.686	2965.431	293.621
lh_inferiortemporal_Vol_QC	105	13925.571	1921.427	187.512
rh_inferiortemporal_Vol_QC	94	13309.021	2045.074	210.933
lh_isthmuscingulate_Vol_QC	120	3467.742	505.474	46.143
rh_isthmuscingulate_Vol_QC	121	3345.736	550.916	50.083
lh_lateraloccipital_Vol_QC	105	15711.686	2249.888	219.567
rh_lateraloccipital_Vol_QC	111	16551.153	2284.791	216.863
lh_lateralorbitofrontal_Vol_QC	113	9583.398	1033.189	97.194
rh_lateralorbitofrontal_Vol_QC	119	9279.655	1200.301	110.031
lh_lingual_Vol_QC	100	8204.380	1273.410	127.341
rh_lingual_Vol_QC	93	8741.032	1268.423	131.529
lh_medialorbitofrontal_Vol_QC	95	6502.632	871.610	89.425
rh_medialorbitofrontal_Vol_QC	92	7031.478	811.574	84.612
lh_middletemporal_Vol_QC	77	13877.052	1583.112	180.412
rh_middletemporal_Vol_QC	74	14863.919	1932.786	224.682
lh_parahippocampal_Vol_QC	120	2286.042	318.770	29.100
rh_parahippocampal_Vol_QC	121	2085.174	298.371	27.125
lh_paracentral_Vol_QC	116	4689.845	585.965	54.405
rh_paracentral_Vol_QC	113	5108.752	636.060	59.835
lh_parsopercularis_Vol_QC	120	6089.042	921.696	84.139
rh_parsopercularis_Vol_QC	117	5126.470	736.725	68.110
lh_parsorbitalis_Vol_QC	120	3469.308	482.269	44.025
rh_parsorbitalis_Vol_QC	120	4093.150	506.679	46.253
lh_parstriangularis_Vol_QC	120	5224.300	825.919	75.396
rh_parstriangularis_Vol_QC	119	6132.647	1019.614	93.468
lh_pericalcarine_Vol_QC	90	2433.133	407.979	43.005
rh_pericalcarine_Vol_QC	87	2595.713	397.739	42.642
lh_postcentral_Vol_QC	95	12649.316	1350.964	138.606
rh_postcentral_Vol_QC	91	12051.374	1543.455	161.798
lh_posteriorcingulate_Vol_QC	119	4090.765	612.381	56.137
rh_posteriorcingulate_Vol_QC	119	4252.025	604.002	55.369
lh_precentral_Vol_QC	105	15936.276	1550.388	151.302
rh_precentral_Vol_QC	99	15745.465	1535.302	154.304
lh_precuneus_Vol_QC	110	14106.827	1830.885	174.568
rh_precuneus_Vol_QC	118	14436.229	1818.102	167.370
lh_rostralanteriorcingulate_Vol_QC	106	2845.557	567.096	55.081
rh_rostralanteriorcingulate_Vol_QC	119	2227.815	480.681	44.064
lh_rostralmiddlefrontal_Vol_QC	119	22641.395	2700.587	247.562
rh_rostralmiddlefrontal_Vol_QC	116	23422.819	3052.386	283.407
lh_superiorfrontal_Vol_QC	81	30113.815	2957.528	328.614
rh_superiorfrontal_Vol_QC	97	28877.557	3097.105	314.463
lh_superiorparietal_Vol_QC	93	19318.452	2884.288	299.087
rh_superiorparietal_Vol_QC	88	18523.875	2294.024	244.544
lh_superiortemporal_Vol_QC	77	15830.195	1621.926	184.836
rh_superiortemporal_Vol_QC	83	14997.759	1615.350	177.308
lh_supramarginal_Vol_QC	86	15501.791	2731.960	294.595
rh_supramarginal_Vol_QC	88	13781.568	2000.392	213.243
lh_frontalpole_Vol_QC	119	1502.252	264.343	24.232
rh_frontalpole_Vol_QC	120	1792.525	266.125	24.294
lh_temporalpole_Vol_QC	117	2541.393	410.071	37.911
rh_temporalpole_Vol_QC	118	2586.178	417.286	38.414
lh_transversetemporal_Vol_QC	121	1518.769	261.108	23.737
rh_transversetemporal_Vol_QC	121	1236.612	194.188	17.653
lh_insula_Vol_QC	100	7710.370	852.446	85.245
rh_insula_Vol_QC	93	7455.172	775.593	80.425

Structural Asymmetry - Volume - No Quality Control

Paired Samples T-Test

Measure 1		Measure 2	t	df	p	Mean Difference	SE Difference	95% CI for Mean Difference		Cohen's d
								Lower	Upper	
lh_bankssts_Vol_no_QC	-	rh_bankssts_Vol_no_QC	8.147	120	< .001	489.017	60.026	370.169	607.864	0.741
lh_caudalanteriorcingulate_Vol_no_QC	-	rh_caudalanteriorcingulate_Vol_no_QC	−6.215	120	< .001	−408.628	65.744	−538.797	−278.460	−0.565
lh_caudalmiddlefrontal_Vol_no_QC	-	rh_caudalmiddlefrontal_Vol_no_QC	3.355	120	0.001	454.628	135.517	186.315	722.941	0.305
lh_cuneus_Vol_no_QC	-	rh_cuneus_Vol_no_QC	−11.409	120	< .001	−550.479	48.250	−646.010	−454.948	−1.037
lh_entorhinal_Vol_no_QC	-	rh_entorhinal_Vol_no_QC	2.787	120	0.006	105.066	37.696	30.431	179.701	0.253
lh_fusiform_Vol_no_QC	-	rh_fusiform_Vol_no_QC	3.911	120	< .001	487.322	124.590	240.643	734.001	0.356
lh_inferiorparietal_Vol_no_QC	-	rh_inferiorparietal_Vol_no_QC	−16.554	120	< .001	−3700.868	223.565	−4143.511	−3258.224	−1.505
lh_inferiortemporal_Vol_no_QC	-	rh_inferiortemporal_Vol_no_QC	2.897	120	0.004	465.149	160.557	147.257	783.041	0.263
lh_isthmuscingulate_Vol_no_QC	-	rh_isthmuscingulate_Vol_no_QC	2.334	120	0.021	112.116	48.038	17.004	207.227	0.212
lh_lateraloccipital_Vol_no_QC	-	rh_lateraloccipital_Vol_no_QC	−5.487	120	< .001	−813.488	148.246	−1107.005	−519.971	−0.499
lh_lateralorbitofrontal_Vol_no_QC	-	rh_lateralorbitofrontal_Vol_no_QC	3.722	120	< .001	218.207	58.624	102.135	334.278	0.338
lh_lingual_Vol_no_QC	-	rh_lingual_Vol_no_QC	−7.725	120	< .001	−677.645	87.716	−851.317	−503.972	−0.702
lh_medialorbitofrontal_Vol_no_QC	-	rh_medialorbitofrontal_Vol_no_QC	−7.495	120	< .001	−568.281	75.822	−718.403	−418.159	−0.681
lh_middletemporal_Vol_no_QC	-	rh_middletemporal_Vol_no_QC	−5.919	120	< .001	−1057.050	178.580	−1410.625	−703.474	−0.538
lh_parahippocampal_Vol_no_QC	-	rh_parahippocampal_Vol_no_QC	7.259	120	< .001	225.694	31.093	164.132	287.256	0.660
lh_paracentral_Vol_no_QC	-	rh_paracentral_Vol_no_QC	−6.623	120	< .001	−420.909	63.555	−546.743	−295.075	−0.602
lh_parsopercularis_Vol_no_QC	-	rh_parsopercularis_Vol_no_QC	10.650	120	< .001	923.554	86.715	751.864	1095.243	0.968
lh_parsorbitalis_Vol_no_QC	-	rh_parsorbitalis_Vol_no_QC	−14.662	120	< .001	−631.926	43.099	−717.258	−546.593	−1.333
lh_parstriangularis_Vol_no_QC	-	rh_parstriangularis_Vol_no_QC	−10.566	120	< .001	−895.529	84.755	−1063.338	−727.720	−0.961
lh_pericalcarine_Vol_no_QC	-	rh_pericalcarine_Vol_no_QC	−8.795	120	< .001	−270.818	30.792	−331.783	−209.853	−0.800
lh_postcentral_Vol_no_QC	-	rh_postcentral_Vol_no_QC	5.243	120	< .001	581.603	110.929	361.972	801.235	0.477
lh_posteriorcingulate_Vol_no_QC	-	rh_posteriorcingulate_Vol_no_QC	−2.451	120	0.016	−156.421	63.816	−282.772	−30.071	−0.223
lh_precentral_Vol_no_QC	-	rh_precentral_Vol_no_QC	1.885	120	0.062	256.331	135.977	−12.894	525.555	0.171
lh_precuneus_Vol_no_QC	-	rh_precuneus_Vol_no_QC	−3.167	120	0.002	−331.413	104.653	−538.619	−124.208	−0.288
lh_rostralanteriorcingulate_Vol_no_QC	-	rh_rostralanteriorcingulate_Vol_no_QC	12.108	120	< .001	670.405	55.368	560.779	780.031	1.101
lh_rostralmiddlefrontal_Vol_no_QC	-	rh_rostralmiddlefrontal_Vol_no_QC	−4.005	120	< .001	−800.240	199.825	−1195.880	−404.599	−0.364
lh_superiorfrontal_Vol_no_QC	-	rh_superiorfrontal_Vol_no_QC	6.560	120	< .001	1336.777	203.764	933.339	1740.215	0.596
lh_superiorparietal_Vol_no_QC	-	rh_superiorparietal_Vol_no_QC	3.823	120	< .001	642.091	167.939	309.583	974.599	0.348
lh_superiortemporal_Vol_no_QC	-	rh_superiortemporal_Vol_no_QC	6.362	120	< .001	881.876	138.625	607.408	1156.344	0.578
lh_supramarginal_Vol_no_QC	-	rh_supramarginal_Vol_no_QC	8.592	120	< .001	1727.446	201.054	1329.373	2125.519	0.781
lh_frontalpole_Vol_no_QC	-	rh_frontalpole_Vol_no_QC	−10.089	120	< .001	−293.884	29.129	−351.558	−236.211	−0.917
lh_temporalpole_Vol_no_QC	-	rh_temporalpole_Vol_no_QC	−0.259	120	0.796	−10.008	38.626	−86.485	66.468	−0.024
lh_transversetemporal_Vol_no_QC	-	rh_transversetemporal_Vol_no_QC	11.897	120	< .001	267.942	22.521	223.352	312.532	1.082
lh_insula_Vol_no_QC	-	rh_insula_Vol_no_QC	3.893	120	< .001	215.364	55.323	105.828	324.899	0.354

Note. Student's t-test.

Descriptives

Descriptives

	N	Mean	SD	SE
lh_bankssts_Vol_no_QC	121	3228.496	706.938	64.267
rh_bankssts_Vol_no_QC	121	2739.479	438.717	39.883
lh_caudalanteriorcingulate_Vol_no_QC	121	1985.124	496.031	45.094
rh_caudalanteriorcingulate_Vol_no_QC	121	2393.752	601.668	54.697
lh_caudalmiddlefrontal_Vol_no_QC	121	8118.851	1331.821	121.075
rh_caudalmiddlefrontal_Vol_no_QC	121	7664.223	1392.905	126.628
lh_cuneus_Vol_no_QC	121	4073.661	628.072	57.097
rh_cuneus_Vol_no_QC	121	4624.140	784.602	71.327
lh_entorhinal_Vol_no_QC	121	2005.058	426.707	38.792
rh_entorhinal_Vol_no_QC	121	1899.992	400.968	36.452
lh_fusiform_Vol_no_QC	121	11908.231	1583.857	143.987
rh_fusiform_Vol_no_QC	121	11420.909	1536.585	139.690
lh_inferiorparietal_Vol_no_QC	121	17280.983	2371.321	215.575
rh_inferiorparietal_Vol_no_QC	121	20981.851	2850.373	259.125
lh_inferiortemporal_Vol_no_QC	121	13779.496	1907.853	173.441
rh_inferiortemporal_Vol_no_QC	121	13314.347	2057.699	187.064
lh_isthmuscingulate_Vol_no_QC	121	3481.298	501.806	45.619
rh_isthmuscingulate_Vol_no_QC	121	3369.182	575.917	52.356
lh_lateraloccipital_Vol_no_QC	121	15765.835	2154.493	195.863
rh_lateraloccipital_Vol_no_QC	121	16579.322	2317.027	210.639
lh_lateralorbitofrontal_Vol_no_QC	121	9512.612	1038.729	94.430
rh_lateralorbitofrontal_Vol_no_QC	121	9294.405	1140.906	103.719
lh_lingual_Vol_no_QC	121	8336.479	1214.873	110.443
rh_lingual_Vol_no_QC	121	9014.124	1396.483	126.953
lh_medialorbitofrontal_Vol_no_QC	121	6404.760	845.048	76.823
rh_medialorbitofrontal_Vol_no_QC	121	6973.041	837.610	76.146
lh_middletemporal_Vol_no_QC	121	13787.967	1678.754	152.614
rh_middletemporal_Vol_no_QC	121	14845.017	1972.669	179.334
lh_parahippocampal_Vol_no_QC	121	2319.851	346.431	31.494
rh_parahippocampal_Vol_no_QC	121	2094.157	329.999	30.000
lh_paracentral_Vol_no_QC	121	4712.884	654.521	59.502
rh_paracentral_Vol_no_QC	121	5133.793	664.810	60.437
lh_parsopercularis_Vol_no_QC	121	6131.711	1005.544	91.413
rh_parsopercularis_Vol_no_QC	121	5208.157	765.107	69.555
lh_parsorbitalis_Vol_no_QC	121	3470.264	500.348	45.486
rh_parsorbitalis_Vol_no_QC	121	4102.190	505.629	45.966
lh_parstriangularis_Vol_no_QC	121	5234.570	810.414	73.674
rh_parstriangularis_Vol_no_QC	121	6130.099	1004.458	91.314
lh_pericalcarine_Vol_no_QC	121	2506.438	464.091	42.190
rh_pericalcarine_Vol_no_QC	121	2777.256	532.060	48.369
lh_postcentral_Vol_no_QC	121	12599.190	1349.496	122.681
rh_postcentral_Vol_no_QC	121	12017.587	1548.988	140.817
lh_posteriorcingulate_Vol_no_QC	121	4105.190	619.289	56.299
rh_posteriorcingulate_Vol_no_QC	121	4261.612	615.051	55.914
lh_precentral_Vol_no_QC	121	15860.074	1555.450	141.405
rh_precentral_Vol_no_QC	121	15603.744	1626.004	147.819
lh_precuneus_Vol_no_QC	121	14197.099	1928.251	175.296
rh_precuneus_Vol_no_QC	121	14528.512	1829.470	166.315
lh_rostralanteriorcingulate_Vol_no_QC	121	2915.397	620.069	56.370
rh_rostralanteriorcingulate_Vol_no_QC	121	2244.992	490.806	44.619
lh_rostralmiddlefrontal_Vol_no_QC	121	22748.686	2728.573	248.052
rh_rostralmiddlefrontal_Vol_no_QC	121	23548.926	3128.706	284.428
lh_superiorfrontal_Vol_no_QC	121	29994.760	2898.356	263.487
rh_superiorfrontal_Vol_no_QC	121	28657.983	2898.100	263.464
lh_superiorparietal_Vol_no_QC	121	19111.372	2821.367	256.488
rh_superiorparietal_Vol_no_QC	121	18469.281	2361.868	214.715
lh_superiortemporal_Vol_no_QC	121	16012.686	1683.399	153.036
rh_superiortemporal_Vol_no_QC	121	15130.810	1536.319	139.665
lh_supramarginal_Vol_no_QC	121	15720.215	2633.429	239.403
rh_supramarginal_Vol_no_QC	121	13992.769	2127.752	193.432
lh_frontalpole_Vol_no_QC	121	1488.884	274.310	24.937
rh_frontalpole_Vol_no_QC	121	1782.769	270.570	24.597
lh_temporalpole_Vol_no_QC	121	2566.959	442.136	40.194
rh_temporalpole_Vol_no_QC	121	2576.967	436.276	39.661
lh_transversetemporal_Vol_no_QC	121	1507.661	263.615	23.965
rh_transversetemporal_Vol_no_QC	121	1239.719	200.661	18.242
lh_insula_Vol_no_QC	121	7914.934	922.295	83.845
rh_insula_Vol_no_QC	121	7699.570	874.909	79.537
