# Supplementary material for: Feasibility of FreeSurfer Processing for T1-Weighted Brain Images of 5-Year-Olds: Semiautomated Protocol of FinnBrain Neuroimaging Lab
Source: Front Neurosci. 2022 May 2;16:874062. doi: 10.3389/fnins.2022.874062 (PMC9108497; doi:10.3389/fnins.2022.874062)
Supplement: Supplementary file 4 [file Table_1.docx]

Feasibility of FreeSurfer processing for T1-weighted brain images of 5-year-olds: semiautomated protocol of FinnBrain Neuroimaging Lab

Supplementary Tables

**Supplementary Table 1**

Participant demographics and maternal medical history variables (number of participants = n = 146)

| Continuous variables | Mean | SD | Min | Max |
| --- | --- | --- | --- | --- |
| Age from birth at scan (years) | 5.35 | 0.06 | 5.08 | 5.52 |
| Gestational age at scan (years) | 6.11 | 0.07 | 5.85 | 6.33 |
| Gestational age at birth (days) | 278 | 12 | 237 | 296 |
| Birth weight (grams) | 3517 | 522 | 1790 | 4980 |
| Maternal age at term (years) | 31.2 | 4.7 | 18.3 | 42.0 |
| Maternal BMI before pregnancy | 24.2 | 4.3 | 17.5 | 42.0 |

| Categorical variables | Number | Percent |
| --- | --- | --- |
| **Sex** | | |
| Male | 79 | 54.1 |
| Female | 67 | 45.9 |
| **Maternal education level** | | |
| Upper secondary school or vocational school or lower | 38 | 26.0 |
| University of applied sciences | 37 | 25.3 |
| University | 66 | 45.2 |
| Missing | 5 | 3.4 |
| **Maternal monthly income, estimated after taxes (euros)** | | |
| ≤ 1 500 | 44 | 30.1 |
| 1 501 – 2 500 | 83 | 56.8 |
| 2 501 – 3 500 | 13 | 8.9 |
| ≥ 3 501 | 1 | 0.7 |
| Missing | 5 | 3.4 |
| **Maternal background** | | |
| Finnish | 141 | 96.6 |
| Other | 5 | 3.4 |
| **Alcohol use during pregnancy** | | |
| Yes, continued to some degree after learning about pregnancy | 13 | 8.9 |
| Yes, stopped after learning about the pregnancy | 27 | 18.5 |
| No | 99 | 67.8 |
| Missing | 7 | 4.8 |
| **Tobacco smoking during pregnancy** | | |
| Yes, continued to some degree after learning about pregnancy | 3 | 2.1 |
| Yes, stopped after learning about the pregnancy | 7 | 4.8 |
| No | 129 | 88.4 |
| Missing | 7 | 4.8 |
| **Illicit drug use during pregnancy** | | |
| No | 139 | 95.2 |
| Missing | 7 | 4.8 |
| **Maternal history of disease, yes (n = 141)** | | |
| Allergies | 59 | 41.8 |
| Depression | 22 | 15.6 |
| Asthma | 13 | 9.2 |
| Anxiety disorder | 13 | 9.2 |
| Eating disorder | 12 | 8.5 |
| Chronic urinary tract infection | 10 | 7.1 |
| Autoimmune disorder | 9 | 6.4 |
| Hypercholesterolemia | 4 | 2.8 |
| Hypertension | 4 | 2.8 |
| Celiac disease | 2 | 1.4 |
| Hypothyroidism | 2 | 1.4 |
| Emphysema | 1 | 0.7 |
| Chronic bacterial or viral infection | 1 | 0.7 |
| Psychosis | 1 | 0.7 |
| Epilepsy | 1 | 0.7 |
| Type 2 diabetes | 1 | 0.7 |
| Drug dependency | 1 | 0.7 |
| Migraine | 1 | 0.7 |
| Other chronic disease | 6 | 4.3 |
| **Maternal medication at gestational week 14, yes (n = 135)** | | |
| Thyroxin | 11 | 8.1 |
| SSRI/SNRI | 6 | 4.4 |
| Corticosteroid | 5 | 3.7 |
| Hypertension medication | 2 | 1.5 |
| Other mood medication | 2 | 1.5 |
| Other medication affecting the CNS | 2 | 1.5 |
| Other medication | 9 | 6.7 |
| **Maternal medication at gestational week 34, yes (n = 140)** | | |
| Thyroxin | 9 | 6.4 |
| SSRI/SNRI | 7 | 5.0 |
| Corticosteroid | 6 | 4.3 |
| Blood pressure medication | 6 | 4.3 |
| NSAID | 1 | 0.7 |
| Other mood medication | 2 | 1.4 |
| Other medication affecting the CNS | 3 | 2.1 |
| Other medication | 27 | 19.3 |

Abbreviations: SD = standard deviation, BMI = body mass index, NSAID = non-steroidal anti-inflammatory drug, SSRI = selective serotonin reuptake inhibitor, SNRI = selective noradrenalin reuptake inhibitor, CNS = central nervous system, GA = gestational age

GA at birth was calculated using the difference between due date and actual date of birth. GA at scan was calculated as age from birth at scan + GA at birth. Maternal age at term was calculated as follows: the age as days at due date divided by 365.25.

On the question about alcohol usage, four subjects answered that they did not use alcohol during pregnancy, but also answered that they stopped using alcohol when they learned about the pregnancy. These were classified as “yes, stopped when learning about pregnancy”.

The data for maternal education level, monthly income estimate, alcohol use, tobacco use, drug use, and diagnostic information are from questionnaires at gestational week 14.

In addition to the diseases in the table, we asked for the following disorders, and none of the mothers suffered from them: myocardial infarction, cardiac dysfunction, angina pectoris, stroke, type 1 diabetes, intellectual disability, alcohol dependency disorder, musculoskeletal disorder, cancer, and attention deficit hyperactivity disorder.

Sex, birth weight, and maternal BMI before pregnancy were retrieved from the National Institute for Health and Welfare (www.thl.fi).

**Supplementary Table 2**

The number of included subjects by quality control protocol. Number of participants = 121.

| Region of interest (ROI) | ENIGMA | Freeview |
| --- | --- | --- |
| Left banks of the superior temporal sulcus | 95 | 121 |
| Right banks of the superior temporal sulcus | 110 | 121 |
| Left caudal anterior cingulate | 96 | 121 |
| Right caudal anterior cingulate | 106 | 121 |
| Left caudal middle frontal | 116 | 116 |
| Right caudal middle frontal | 114 | 109 |
| Left cuneus | 93 | 119 |
| Right cuneus | 92 | 120 |
| Left entorhinal | 121 | 119 |
| Right entorhinal | 120 | 117 |
| Left fusiform | 120 | 120 |
| Right fusiform | 121 | 115 |
| Left inferior parietal | 102 | 120 |
| Right inferior parietal | 102 | 113 |
| Left inferior temporal | 105 | 115 |
| Right inferior temporal | 94 | 104 |
| Left isthmus cingulate | 120 | 119 |
| Right isthmus cingulate | 121 | 119 |
| Left lateral occipital | 105 | 116 |
| Right lateral occipital | 111 | 117 |
| Left lateral orbitofrontal | 113 | 113 |
| Right lateral orbitofrontal | 119 | 114 |
| Left lingual | 100 | 92 |
| Right lingual | 93 | 99 |
| Left medial orbitofrontal | 95 | 113 |
| Right medial orbitofrontal | 92 | 113 |
| Left middle temporal | 77 | 119 |
| Right middle temporal | 74 | 114 |
| Left parahippocampal | 120 | 121 |
| Right parahippocampal | 121 | 120 |
| Left paracentral | 116 | 120 |
| Right paracentral | 113 | 119 |
| Left pars opercularis | 120 | 117 |
| Right pars opercularis | 117 | 118 |
| Left pars orbitalis | 120 | 121 |
| Right pars orbitalis | 120 | 120 |
| Left pars triangularis | 120 | 121 |
| Right pars triangularis | 119 | 119 |
| Left pericalcarine | 90 | 113 |
| Right pericalcarine | 87 | 119 |
| Left postcentral | 95 | 101 |
| Right postcentral | 91 | 96 |
| Left posterior cingulate | 119 | 121 |
| Right posterior cingulate | 119 | 121 |
| Left precentral | 105 | 110 |
| Right precentral | 99 | 110 |
| Left precuneus | 110 | 91 |
| Right precuneus | 118 | 104 |
| Left rostral anterior cingulate | 106 | 121 |
| Right rostral anterior cingulate | 119 | 121 |
| Left rostral middle frontal | 119 | 117 |
| Right rostral middle frontal | 116 | 116 |
| Left superior frontal | 81 | 110 |
| Right superior frontal | 97 | 97 |
| Left superior parietal | 93 | 110 |
| Right superior parietal | 88 | 105 |
| Left superior temporal | 77 | 116 |
| Right superior temporal | 83 | 116 |
| Left supramarginal | 86 | 114 |
| Right supramarginal | 88 | 117 |
| Left frontal pole | 119 | 121 |
| Right frontal pole | 120 | 121 |
| Left temporal pole | 117 | 121 |
| Right temporal pole | 118 | 121 |
| Left transverse temporal | 121 | 121 |
| Right transverse temporal | 121 | 121 |
| Left insula | 100 | 119 |
| Right insula | 93 | 118 |

**Supplementary Table 3**

Effects of manual edits on cortical thickness measurements.

| ROI | N | P-value | Mean difference (mm) between FreeSurfer edited and unedited |
| --- | --- | --- | --- |
| Left banks of the superior temporal sulcus | 95 | 0.0484 | -0.0078 |
| Right banks of the superior temporal sulcus | 110 | 0.0067 | -0.0111 |
| Left caudal anterior cingulate | 96 | 0.0054 | -0.0232 |
| Right caudal anterior cingulate | 106 | 0.0467 | -0.0170 |
| Left caudal middle frontal | 116 | 8.5E-7* | 0.0397 |
| Right caudal middle frontal | 114 | 9.9E-6* | 0.0526 |
| Left cuneus | 93 | 0.0898 | -0.0085 |
| Right cuneus | 92 | 0.2097 | -0.0057 |
| Left entorhinal | 121 | 0.3554 | -0.0137 |
| Right entorhinal | 120 | 0.2751 | 0.0167 |
| Left fusiform | 120 | 0.0502 | -0.0067 |
| Right fusiform | 121 | 0.0187 | 0.0102 |
| Left inferior parietal | 102 | 0.1688 | 0.0062 |
| Right inferior parietal | 102 | 0.0009 | 0.0181 |
| Left inferior temporal | 105 | 1.1E-7* | 0.0318 |
| Right inferior temporal | 94 | 5.1E-10* | 0.0476 |
| Left isthmus cingulate | 120 | 0.6259 | -0.0030 |
| Right isthmus cingulate | 121 | 0.0072 | -0.0160 |
| Left lateral occipital | 105 | 0.0824 | 0.0043 |
| Right lateral occipital | 111 | 0.8098 | 0.0005 |
| Left lateral orbitofrontal | 113 | 0.3145 | 0.0070 |
| Right lateral orbitofrontal | 119 | 0.1183 | -0.0142 |
| Left lingual | 100 | 0.7649 | -0.0012 |
| Right lingual | 93 | 0.2775 | -0.0042 |
| Left medial orbitofrontal | 95 | 0.4053 | -0.0089 |
| Right medial orbitofrontal | 92 | 0.3400 | -0.0089 |
| Left middle temporal | 77 | 0.3906 | -0.0032 |
| Right middle temporal | 74 | 0.0434 | -0.0081 |
| Left parahippocampal | 120 | 0.1335 | 0.0138 |
| Right parahippocampal | 121 | 0.6406 | 0.0041 |
| Left paracentral | 116 | 0.0684 | 0.0087 |
| Right paracentral | 113 | 0.1956 | 0.0053 |
| Left pars opercularis | 120 | 0.3156 | -0.0042 |
| Right pars opercularis | 117 | 0.0045 | -0.0116 |
| Left pars orbitalis | 120 | 0.6716 | 0.0034 |
| Right pars orbitalis | 120 | 0.1788 | -0.0086 |
| Left pars triangularis | 120 | 0.7204 | -0.0018 |
| Right pars triangularis | 119 | 0.0536 | -0.0073 |
| Left pericalcarine | 90 | 0.7953 | 0.0012 |
| Right pericalcarine | 87 | 0.7017 | 0.0017 |
| Left postcentral | 95 | 0.0368 | 0.0080 |
| Right postcentral | 91 | 0.0108 | 0.0120 |
| Left posterior cingulate | 119 | 0.0670 | -0.0085 |
| Right posterior cingulate | 119 | 0.1200 | -0.0063 |
| Left precentral | 105 | 0.0050 | 0.0136 |
| Right precentral | 99 | 7.1E-6* | 0.0306 |
| Left precuneus | 110 | 0.8234 | 0.0007 |
| Right precuneus | 118 | 0.1537 | -0.0043 |
| Left rostral anterior cingulate | 106 | 0.0950 | -0.0215 |
| Right rostral anterior cingulate | 119 | 2.7E-6* | -0.0450 |
| Left rostral middle frontal | 119 | 0.9397 | -0.0003 |
| Right rostral middle frontal | 116 | 0.7498 | -0.0012 |
| Left superior frontal | 81 | 0.0048 | 0.0197 |
| Right superior frontal | 97 | 0.0001* | 0.0309 |
| Left superior parietal | 93 | 2.1E-6* | 0.0273 |
| Right superior parietal | 88 | 4.4E-8* | 0.0367 |
| Left superior temporal | 77 | 0.0940 | -0.0076 |
| Right superior temporal | 83 | 0.0003* | -0.0135 |
| Left supramarginal | 86 | 0.2724 | -0.0043 |
| Right supramarginal | 88 | 0.0003* | 0.0297 |
| Left frontal pole | 119 | 0.4355 | 0.0110 |
| Right frontal pole | 120 | 0.8574 | -0.0029 |
| Left temporal pole | 117 | 0.5564 | -0.0099 |
| Right temporal pole | 118 | 0.2801 | -0.0185 |
| Left transverse temporal | 121 | 0.7485 | 0.0026 |
| Right transverse temporal | 121 | 0.3153 | -0.0105 |
| Left insula | 100 | 0.2719 | 0.0130 |
| Right insula | 93 | 0.0399 | 0.0195 |

Abbreviations: ROI = region of interest, N = number of participants, * = statistically significant after Bonferroni correction (p < 0.0007). Positive mean difference indicates edited being thicker than unedited.

**Supplementary Table 4**

Pearson’s correlation between edited and unedited FreeSurfer cortical thickness measurements.

| ROI | N | P-value | Correlation |
| --- | --- | --- | --- |
| Left banks of the superior temporal sulcus | 95 | 4.1E-71* | 0.984 |
| Right banks of the superior temporal sulcus | 110 | 8.8E-76* | 0.978 |
| Left caudal anterior cingulate | 96 | 1.1E-55* | 0.964 |
| Right caudal anterior cingulate | 106 | 2.0E-49* | 0.937 |
| Left caudal middle frontal | 116 | 1.8E-38* | 0.879 |
| Right caudal middle frontal | 114 | 9.5E-22* | 0.749 |
| Left cuneus | 93 | 5.0E-48* | 0.951 |
| Right cuneus | 92 | 7.6E-52* | 0.961 |
| Left entorhinal | 121 | 8.4E-38* | 0.867 |
| Right entorhinal | 120 | 6.1E-41* | 0.885 |
| Left fusiform | 120 | 4.5E-59* | 0.945 |
| Right fusiform | 121 | 3.8E-47* | 0.909 |
| Left inferior parietal | 102 | 1.8E-44* | 0.927 |
| Right inferior parietal | 102 | 5.7E-45* | 0.929 |
| Left inferior temporal | 105 | 3.8E-44* | 0.922 |
| Right inferior temporal | 94 | 3.5E-37* | 0.911 |
| Left isthmus cingulate | 120 | 2.4E-55* | 0.936 |
| Right isthmus cingulate | 121 | 3.9E-60* | 0.946 |
| Left lateral occipital | 105 | 5.0E-72* | 0.978 |
| Right lateral occipital | 111 | 1.3E-80* | 0.982 |
| Left lateral orbitofrontal | 113 | 2.9E-39* | 0.888 |
| Right lateral orbitofrontal | 119 | 5.2E-29* | 0.811 |
| Left lingual | 100 | 1.0E-58* | 0.965 |
| Right lingual | 93 | 6.5E-54* | 0.964 |
| Left medial orbitofrontal | 95 | 1.2E-25* | 0.833 |
| Right medial orbitofrontal | 92 | 3.2E-28* | 0.861 |
| Left middle temporal | 77 | 2.2E-47* | 0.969 |
| Right middle temporal | 74 | 1.4E-46* | 0.971 |
| Left parahippocampal | 120 | 3.1E-56* | 0.938 |
| Right parahippocampal | 121 | 2.2E-50* | 0.920 |
| Left paracentral | 116 | 8.5E-55* | 0.939 |
| Right paracentral | 113 | 6.7E-56* | 0.945 |
| Left pars opercularis | 120 | 7.4E-55* | 0.935 |
| Right pars opercularis | 117 | 2.5E-56* | 0.942 |
| Left pars orbitalis | 120 | 1.3E-50* | 0.922 |
| Right pars orbitalis | 120 | 2.0E-65* | 0.957 |
| Left pars triangularis | 120 | 1.1E-59* | 0.946 |
| Right pars triangularis | 119 | 1.0E-69* | 0.965 |
| Left pericalcarine | 90 | 1.9E-49* | 0.958 |
| Right pericalcarine | 87 | 9.4E-47* | 0.955 |
| Left postcentral | 95 | 4.7E-53* | 0.960 |
| Right postcentral | 91 | 6.7E-45* | 0.945 |
| Left posterior cingulate | 119 | 1.2E-61* | 0.951 |
| Right posterior cingulate | 119 | 1.6E-66* | 0.960 |
| Left precentral | 105 | 1.7E-42* | 0.915 |
| Right precentral | 99 | 9.9E-32* | 0.871 |
| Left precuneus | 110 | 7.5E-62* | 0.961 |
| Right precuneus | 118 | 1.2E-62* | 0.954 |
| Left rostral anterior cingulate | 106 | 1.5E-31* | 0.856 |
| Right rostral anterior cingulate | 119 | 7.6E-48* | 0.915 |
| Left rostral middle frontal | 119 | 1.4E-64* | 0.957 |
| Right rostral middle frontal | 116 | 1.2E-58* | 0.948 |
| Left superior frontal | 81 | 2.0E-33* | 0.918 |
| Right superior frontal | 97 | 6.2E-32* | 0.877 |
| Left superior parietal | 93 | 5.5E-38* | 0.917 |
| Right superior parietal | 88 | 1.6E-28* | 0.873 |
| Left superior temporal | 77 | 1.9E-43* | 0.961 |
| Right superior temporal | 83 | 1.2E-55* | 0.976 |
| Left supramarginal | 86 | 2.4E-50* | 0.964 |
| Right supramarginal | 88 | 2.2E-25* | 0.848 |
| Left frontal pole | 119 | 3.7E-44* | 0.901 |
| Right frontal pole | 120 | 1.0E-33* | 0.844 |
| Left temporal pole | 117 | 5.5E-31* | 0.830 |
| Right temporal pole | 118 | 3.5E-34* | 0.851 |
| Left transverse temporal | 121 | 6.9E-57* | 0.939 |
| Right transverse temporal | 121 | 4.5E-47* | 0.909 |
| Left insula | 100 | 1.4E-17* | 0.725 |
| Right insula | 93 | 1.2E-26* | 0.846 |

Abbreviations: ROI = region of interest, N = number of participants, * = statistically significant after Bonferroni correction (p < 0.0007).

**Supplementary Table 5**

Effects of manual edits on surface area measurements.

| ROI | N | P-value | Mean difference (mm^2^) between FreeSurfer edited and unedited |
| --- | --- | --- | --- |
| Left banks of the superior temporal sulcus | 95 | 0.4028 | -3.20 |
| Right banks of the superior temporal sulcus | 110 | 0.9031 | 0.39 |
| Left caudal anterior cingulate | 96 | 0.0303 | 4.65 |
| Right caudal anterior cingulate | 106 | 0.8422 | 0.84 |
| Left caudal middle frontal | 116 | 0.0001* | -33.00 |
| Right caudal middle frontal | 114 | 0.0135 | -46.06 |
| Left cuneus | 93 | 0.2357 | 7.94 |
| Right cuneus | 92 | 0.0174 | -12.41 |
| Left entorhinal | 121 | 0.1926 | 5.48 |
| Right entorhinal | 120 | 0.0576 | 7.43 |
| Left fusiform | 120 | 0.6990 | -2.87 |
| Right fusiform | 121 | 0.1002 | -12.39 |
| Left inferior parietal | 102 | 0.1313 | -18.07 |
| Right inferior parietal | 102 | 0.0020 | -74.66 |
| Left inferior temporal | 105 | 0.3506 | -7.39 |
| Right inferior temporal | 94 | 4.3E-6* | -49.38 |
| Left isthmus cingulate | 120 | 0.5314 | -2.17 |
| Right isthmus cingulate | 121 | 0.1555 | -5.45 |
| Left lateral occipital | 105 | 0.2990 | -11.40 |
| Right lateral occipital | 111 | 0.0084 | -28.43 |
| Left lateral orbitofrontal | 113 | 0.6321 | 5.10 |
| Right lateral orbitofrontal | 119 | 0.9777 | -0.52 |
| Left lingual | 100 | 0.0599 | -12.21 |
| Right lingual | 93 | 0.0756 | 12.25 |
| Left medial orbitofrontal | 95 | 0.0372 | 32.60 |
| Right medial orbitofrontal | 92 | 0.6437 | 5.30 |
| Left middle temporal | 77 | 0.7893 | -2.01 |
| Right middle temporal | 74 | 0.9365 | 0.53 |
| Left parahippocampal | 120 | 0.0272 | -7.86 |
| Right parahippocampal | 121 | 0.0902 | -6.15 |
| Left paracentral | 116 | 0.0740 | -6.89 |
| Right paracentral | 113 | 0.0189 | -13.11 |
| Left pars opercularis | 120 | 0.1965 | -5.98 |
| Right pars opercularis | 117 | 0.1218 | -8.39 |
| Left pars orbitalis | 120 | 0.3589 | -2.75 |
| Right pars orbitalis | 120 | 0.9201 | -0.26 |
| Left pars triangularis | 120 | 0.2036 | -5.19 |
| Right pars triangularis | 119 | 0.4430 | 4.39 |
| Left pericalcarine | 90 | 0.2238 | 9.26 |
| Right pericalcarine | 87 | 0.7803 | -1.38 |
| Left postcentral | 95 | 0.0005* | -35.75 |
| Right postcentral | 91 | 0.0001* | -43.48 |
| Left posterior cingulate | 119 | 0.3669 | -2.61 |
| Right posterior cingulate | 119 | 0.3572 | 3.19 |
| Left precentral | 105 | 0.0007* | -41.08 |
| Right precentral | 99 | 5.8E-6* | -90.58 |
| Left precuneus | 110 | 0.0022 | -18.33 |
| Right precuneus | 118 | 0.1058 | -20.37 |
| Left rostral anterior cingulate | 106 | 0.4704 | 3.83 |
| Right rostral anterior cingulate | 119 | 0.0238 | 8.37 |
| Left rostral middle frontal | 119 | 0.0125 | -34.45 |
| Right rostral middle frontal | 116 | 0.6149 | -10.66 |
| Left superior frontal | 81 | 0.0002* | -93.93 |
| Right superior frontal | 97 | 0.0040 | -100.02 |
| Left superior parietal | 93 | 5.8E-6* | -80.89 |
| Right superior parietal | 88 | 2.4E-5* | -161.05 |
| Left superior temporal | 77 | 0.9437 | 0.62 |
| Right superior temporal | 83 | 0.3865 | -5.29 |
| Left supramarginal | 86 | 0.5994 | -4.57 |
| Right supramarginal | 88 | 0.0072 | -64.55 |
| Left frontal pole | 119 | 0.6358 | 1.03 |
| Right frontal pole | 120 | 0.4781 | 1.79 |
| Left temporal pole | 117 | 0.6238 | -1.82 |
| Right temporal pole | 118 | 0.2335 | 4.33 |
| Left transverse temporal | 121 | 0.0049 | 5.59 |
| Right transverse temporal | 121 | 0.6921 | 0.70 |
| Left insula | 100 | 0.0001* | -61.57 |
| Right insula | 93 | 4.6E-5* | -66.41 |

Abbreviations: ROI = region of interest, N = number of participants, * = statistically significant after Bonferroni correction (p < 0.0007). Positive mean difference indicates edited having larger area than unedited.

**Supplementary Table 6**

Pearson’s correlation between edited and unedited FreeSurfer surface area measurements.

| ROI | N | P-value | Correlation |
| --- | --- | --- | --- |
| Left banks of the superior temporal sulcus | 95 | 1.6E-66* | 0.980 |
| Right banks of the superior temporal sulcus | 110 | 6.8E-68* | 0.970 |
| Left caudal anterior cingulate | 96 | 7.9E-68* | 0.980 |
| Right caudal anterior cingulate | 106 | 1.8E-55* | 0.952 |
| Left caudal middle frontal | 116 | 3.6E-77* | 0.976 |
| Right caudal middle frontal | 114 | 1.0E-34* | 0.861 |
| Left cuneus | 93 | 8.2E-51* | 0.957 |
| Right cuneus | 92 | 1.3E-58* | 0.972 |
| Left entorhinal | 121 | 2.2E-41* | 0.885 |
| Right entorhinal | 120 | 1.6E-38* | 0.873 |
| Left fusiform | 120 | 7.5E-82* | 0.978 |
| Right fusiform | 121 | 5.9E-85* | 0.980 |
| Left inferior parietal | 102 | 8.0E-82* | 0.987 |
| Right inferior parietal | 102 | 7.0E-60* | 0.965 |
| Left inferior temporal | 105 | 4.2E-81* | 0.985 |
| Right inferior temporal | 94 | 2.5E-67* | 0.981 |
| Left isthmus cingulate | 120 | 6.3E-75* | 0.971 |
| Right isthmus cingulate | 121 | 5.1E-76* | 0.971 |
| Left lateral occipital | 105 | 2.6E-86* | 0.988 |
| Right lateral occipital | 111 | 1.3E-92* | 0.989 |
| Left lateral orbitofrontal | 113 | 4.0E-49* | 0.927 |
| Right lateral orbitofrontal | 119 | 1.4E-38* | 0.875 |
| Left lingual | 100 | 5.7E-85* | 0.990 |
| Right lingual | 93 | 1.4E-75* | 0.988 |
| Left medial orbitofrontal | 95 | 1.0E-24* | 0.824 |
| Right medial orbitofrontal | 92 | 1.1E-38* | 0.921 |
| Left middle temporal | 77 | 6.7E-60* | 0.986 |
| Right middle temporal | 74 | 9.5E-67* | 0.992 |
| Left parahippocampal | 120 | 4.4E-44* | 0.899 |
| Right parahippocampal | 121 | 9.5E-41* | 0.882 |
| Left paracentral | 116 | 3.3E-75* | 0.974 |
| Right paracentral | 113 | 5.1E-64* | 0.961 |
| Left pars opercularis | 120 | 2.3E-90* | 0.984 |
| Right pars opercularis | 117 | 5.0E-68* | 0.964 |
| Left pars orbitalis | 120 | 2.3E-57* | 0.941 |
| Right pars orbitalis | 120 | 9.8E-76* | 0.972 |
| Left pars triangularis | 120 | 8.2E-86* | 0.981 |
| Right pars triangularis | 119 | 3.8E-80* | 0.977 |
| Left pericalcarine | 90 | 1.3E-45* | 0.948 |
| Right pericalcarine | 87 | 6.8E-57* | 0.974 |
| Left postcentral | 95 | 1.2E-63* | 0.976 |
| Right postcentral | 91 | 5.6E-67* | 0.983 |
| Left posterior cingulate | 119 | 4.4E-92* | 0.986 |
| Right posterior cingulate | 119 | 8.6E-84* | 0.980 |
| Left precentral | 105 | 2.1E-64* | 0.969 |
| Right precentral | 99 | 1.3E-45* | 0.935 |
| Left precuneus | 110 | 3.5E-106* | 0.994 |
| Right precuneus | 118 | 1.5E-75* | 0.973 |
| Left rostral anterior cingulate | 106 | 5.8E-51* | 0.942 |
| Right rostral anterior cingulate | 119 | 3.1E-59* | 0.946 |
| Left rostral middle frontal | 119 | 2.5E-92* | 0.986 |
| Right rostral middle frontal | 116 | 6.5E-71* | 0.969 |
| Left superior frontal | 81 | 9.5E-55* | 0.977 |
| Right superior frontal | 97 | 6.1E-45* | 0.936 |
| Left superior parietal | 93 | 5.0E-72* | 0.986 |
| Right superior parietal | 88 | 1.9E-34* | 0.909 |
| Left superior temporal | 77 | 2.6E-57* | 0.983 |
| Right superior temporal | 83 | 1.1E-71* | 0.991 |
| Left supramarginal | 86 | 4.2E-86* | 0.995 |
| Right supramarginal | 88 | 2.5E-39* | 0.931 |
| Left frontal pole | 119 | 8.7E-17* | 0.669 |
| Right frontal pole | 120 | 4.2E-27* | 0.793 |
| Left temporal pole | 117 | 6.2E-23* | 0.756 |
| Right temporal pole | 118 | 7.8E-27* | 0.794 |
| Left transverse temporal | 121 | 1.9E-67* | 0.960 |
| Right transverse temporal | 121 | 2.2E-54* | 0.932 |
| Left insula | 100 | 9.4E-27* | 0.831 |
| Right insula | 93 | 2.0E-28* | 0.861 |

Abbreviations: ROI = region of interest, N = number of participants, * = statistically significant after Bonferroni correction (p < 0.0007).

**Supplementary Table 7**

Effects of manual edits on volume measurements.

| ROI | N | P-value | Mean difference (mm^3^) between FreeSurfer edited and unedited |
| --- | --- | --- | --- |
| Left banks of the superior temporal sulcus | 95 | 0.5223 | -8.82 |
| Right banks of the superior temporal sulcus | 110 | 0.5777 | -6.81 |
| Left caudal anterior cingulate | 96 | 0.2857 | -8.23 |
| Right caudal anterior cingulate | 106 | 0.1700 | -21.55 |
| Left caudal middle frontal | 116 | 0.0014 | 81.13 |
| Right caudal middle frontal | 114 | 0.0474 | 101.74 |
| Left cuneus | 93 | 0.9535 | -0.83 |
| Right cuneus | 92 | 0.0330 | -33.63 |
| Left entorhinal | 121 | 0.9512 | 1.29 |
| Right entorhinal | 120 | 0.0028 | 55.19 |
| Left fusiform | 120 | 0.1526 | -44.55 |
| Right fusiform | 121 | 0.4466 | 20.64 |
| Left inferior parietal | 102 | 0.7900 | 8.37 |
| Right inferior parietal | 102 | 0.6782 | -22.06 |
| Left inferior temporal | 105 | 0.0285 | 76.12 |
| Right inferior temporal | 94 | 0.6288 | 18.28 |
| Left isthmus cingulate | 120 | 0.3079 | -9.99 |
| Right isthmus cingulate | 121 | 0.0506 | -23.45 |
| Left lateral occipital | 105 | 0.7107 | 10.90 |
| Right lateral occipital | 111 | 0.0342 | -65.35 |
| Left lateral orbitofrontal | 113 | 0.0639 | 51.43 |
| Right lateral orbitofrontal | 119 | 0.5362 | -24.98 |
| Left lingual | 100 | 0.1148 | -26.68 |
| Right lingual | 93 | 0.8133 | 3.97 |
| Left medial orbitofrontal | 95 | 0.1062 | 63.17 |
| Right medial orbitofrontal | 92 | 0.8951 | -4.77 |
| Left middle temporal | 77 | 0.4013 | -33.21 |
| Right middle temporal | 74 | 0.3223 | -34.41 |
| Left parahippocampal | 120 | 0.0662 | -23.48 |
| Right parahippocampal | 121 | 0.5231 | -8.98 |
| Left paracentral | 116 | 0.7836 | 3.63 |
| Right paracentral | 113 | 0.3481 | -17.29 |
| Left pars opercularis | 120 | 0.3797 | -13.86 |
| Right pars opercularis | 117 | 0.0127 | -42.57 |
| Left pars orbitalis | 120 | 0.5329 | -8.43 |
| Right pars orbitalis | 120 | 0.4693 | -9.09 |
| Left pars triangularis | 120 | 0.2758 | -17.81 |
| Right pars triangularis | 119 | 0.6833 | 9.66 |
| Left pericalcarine | 90 | 0.2022 | 14.33 |
| Right pericalcarine | 87 | 0.8935 | -1.55 |
| Left postcentral | 95 | 0.1537 | -35.34 |
| Right postcentral | 91 | 0.8917 | 2.95 |
| Left posterior cingulate | 119 | 0.2047 | -12.74 |
| Right posterior cingulate | 119 | 0.8743 | -1.66 |
| Left precentral | 105 | 0.4756 | 13.23 |
| Right precentral | 99 | 0.0709 | 72.69 |
| Left precuneus | 110 | 0.1762 | -28.38 |
| Right precuneus | 118 | 0.1794 | -46.51 |
| Left rostral anterior cingulate | 106 | 0.3607 | -13.73 |
| Right rostral anterior cingulate | 119 | 0.8798 | -1.91 |
| Left rostral middle frontal | 119 | 0.1054 | -65.94 |
| Right rostral middle frontal | 116 | 0.8935 | -9.53 |
| Left superior frontal | 81 | 0.8039 | -15.07 |
| Right superior frontal | 97 | 0.4825 | 68.34 |
| Left superior parietal | 93 | 0.0146 | 92.86 |
| Right superior parietal | 88 | 0.9641 | -3.58 |
| Left superior temporal | 77 | 0.2486 | -54.64 |
| Right superior temporal | 83 | 0.0097 | -81.81 |
| Left supramarginal | 86 | 0.2453 | -36.05 |
| Right supramarginal | 88 | 0.8452 | -14.52 |
| Left frontal pole | 119 | 0.5062 | 8.12 |
| Right frontal pole | 120 | 0.5139 | 11.50 |
| Left temporal pole | 117 | 0.2739 | -30.64 |
| Right temporal pole | 118 | 0.8251 | -5.81 |
| Left transverse temporal | 121 | 0.1025 | 11.11 |
| Right transverse temporal | 121 | 0.6383 | -3.11 |
| Left insula | 100 | 9.0E-6* | -189.56 |
| Right insula | 93 | 7.0E-6* | -180.55 |

Abbreviations: ROI = region of interest, N = number of participants, * = statistically significant after Bonferroni correction (p < 0.0007). Positive mean difference indicates edited having larger volume than unedited.

**Supplementary Table 8**

Pearson’s correlation between edited and unedited FreeSurfer volume measurements.

| ROI | N | P-value | Correlation |
| --- | --- | --- | --- |
| Left banks of the superior temporal sulcus | 95 | 1.8E-61* | 0.974 |
| Right banks of the superior temporal sulcus | 110 | 5.3E-59* | 0.955 |
| Left caudal anterior cingulate | 96 | 1.0E-75* | 0.987 |
| Right caudal anterior cingulate | 106 | 3.2E-58* | 0.958 |
| Left caudal middle frontal | 116 | 4.2E-78* | 0.977 |
| Right caudal middle frontal | 114 | 4.6E-43* | 0.904 |
| Left cuneus | 93 | 4.7E-61* | 0.975 |
| Right cuneus | 92 | 2.6E-61* | 0.976 |
| Left entorhinal | 121 | 1.0E-33* | 0.842 |
| Right entorhinal | 120 | 1.1E-38* | 0.873 |
| Left fusiform | 120 | 1.2E-80* | 0.977 |
| Right fusiform | 121 | 9.6E-87* | 0.981 |
| Left inferior parietal | 102 | 1.5E-90* | 0.992 |
| Right inferior parietal | 102 | 3.1E-76* | 0.984 |
| Left inferior temporal | 105 | 4.7E-78* | 0.983 |
| Right inferior temporal | 94 | 1.0E-70* | 0.984 |
| Left isthmus cingulate | 120 | 1.2E-81* | 0.978 |
| Right isthmus cingulate | 121 | 1.1E-78* | 0.974 |
| Left lateral occipital | 105 | 4.8E-92* | 0.991 |
| Right lateral occipital | 111 | 9.9E-97* | 0.991 |
| Left lateral orbitofrontal | 113 | 2.2E-63* | 0.960 |
| Right lateral orbitofrontal | 119 | 5.0E-53* | 0.931 |
| Left lingual | 100 | 1.4E-88* | 0.991 |
| Right lingual | 93 | 1.1E-83* | 0.992 |
| Left medial orbitofrontal | 95 | 2.7E-36* | 0.905 |
| Right medial orbitofrontal | 92 | 6.7E-37* | 0.913 |
| Left middle temporal | 77 | 4.4E-52* | 0.977 |
| Right middle temporal | 74 | 8.6E-61* | 0.988 |
| Left parahippocampal | 120 | 1.5E-46* | 0.909 |
| Right parahippocampal | 121 | 3.8E-41* | 0.884 |
| Left paracentral | 116 | 1.7E-73* | 0.972 |
| Right paracentral | 113 | 5.8E-61* | 0.956 |
| Left pars opercularis | 120 | 3.2E-90* | 0.984 |
| Right pars opercularis | 117 | 3.7E-72* | 0.970 |
| Left pars orbitalis | 120 | 5.7E-64* | 0.955 |
| Right pars orbitalis | 120 | 2.5E-69* | 0.963 |
| Left pars triangularis | 120 | 1.9E-80* | 0.976 |
| Right pars triangularis | 119 | 5.4E-72* | 0.968 |
| Left pericalcarine | 90 | 2.1E-53* | 0.966 |
| Right pericalcarine | 87 | 4.9E-50* | 0.963 |
| Left postcentral | 95 | 6.3E-72* | 0.984 |
| Right postcentral | 91 | 1.1E-79* | 0.991 |
| Left posterior cingulate | 119 | 2.6E-90* | 0.985 |
| Right posterior cingulate | 119 | 1.0E-86* | 0.982 |
| Left precentral | 105 | 2.0E-96* | 0.993 |
| Right precentral | 99 | 1.4E-59* | 0.967 |
| Left precuneus | 110 | 1.4E-101* | 0.993 |
| Right precuneus | 118 | 7.3E-82* | 0.979 |
| Left rostral anterior cingulate | 106 | 2.3E-61* | 0.964 |
| Right rostral anterior cingulate | 119 | 5.8E-66* | 0.959 |
| Left rostral middle frontal | 119 | 1.5E-94* | 0.987 |
| Right rostral middle frontal | 116 | 5.0E-71* | 0.969 |
| Left superior frontal | 81 | 5.0E-60* | 0.983 |
| Right superior frontal | 97 | 2.4E-50* | 0.951 |
| Left superior parietal | 93 | 2.9E-84* | 0.992 |
| Right superior parietal | 88 | 4.4E-44* | 0.947 |
| Left superior temporal | 77 | 5.3E-47* | 0.968 |
| Right superior temporal | 83 | 2.7E-63* | 0.985 |
| Left supramarginal | 86 | 5.5E-85* | 0.995 |
| Right supramarginal | 88 | 5.7E-42* | 0.940 |
| Left frontal pole | 119 | 2.6E-39* | 0.878 |
| Right frontal pole | 120 | 2.3E-22* | 0.744 |
| Left temporal pole | 117 | 1.8E-22* | 0.751 |
| Right temporal pole | 118 | 2.6E-24* | 0.769 |
| Left transverse temporal | 121 | 1.2E-67* | 0.960 |
| Right transverse temporal | 121 | 1.1E-54* | 0.933 |
| Left insula | 100 | 1.3E-38* | 0.907 |
| Right insula | 93 | 5.4E-35* | 0.902 |

Abbreviations: ROI = region of interest, N = number of participants, * = statistically significant after Bonferroni correction (p < 0.0007).
